# Intramolecular cascade annulation triggered by rhodium(III)-catalyzed sequential C(sp^2^)–H activation and C(sp^3^)–H amination

**DOI:** 10.3762/bjoc.15.52

**Published:** 2019-02-27

**Authors:** Liangliang Song, Guilong Tian, Johan Van der Eycken, Erik V Van der Eycken

**Affiliations:** 1 Laboratory for Organic & Microwave-Assisted Chemistry (LOMAC), Department of Chemistry, KU Leuven, Celestijnenlaan 200F, 3001, Leuven, Belgium; 2 Laboratory for Organic and Bio-Organic Synthesis, Department of Organic and Macromolecular Chemistry, Ghent University, Krijgslaan 281 (S.4), B-9000 Ghent, Belgium; 3 Peoples’ Friendship University of Russia (RUDN University), Miklukho-Maklaya Street 6, Moscow, Russia

**Keywords:** annulation, C–H activation, rhodium, acrylamide, heterocycles

## Abstract

A rhodium(III)-catalyzed intramolecular oxidative annulation of *O*-substituted *N*-hydroxyacrylamides for the construction of indolizinones via sequential C(sp^2^)–H activation and C(sp^3^)–H amination has been developed. This approach shows excellent functional-group tolerance. The synthesized scaffold forms the core of many natural products with pharmacological relevance.

## Introduction

Over the last decade, transition metal-catalyzed C(sp^2^)–H activation has emerged as an efficient strategy to access complex molecules [1–6]. Among the methodologies, Rh^III^-catalyzed oxidative annulation of a C(sp^2^)–H bond with 2π components (such as olefins, alkynes) stands out for the construction of carbo(hetero)cycles from easily available starting materials [7–10]. Compared to aromatic C(sp^2^)–H bonds, studies on activation of vinylic C(sp^2^)–H bonds have been less explored, due to an intrinsic inactivity, tended to undergo polymerization, prone to go through conjugate additions [11–12]. Moreover, the cyclometalation intermediates are unstable, and the β-substitution or α,β-disubstitution of acrylate sterically prevents the cyclometalation [13–14]. Despite these, several approaches have been developed to synthesize pyridones and highly substituted olefins using acrylamides. However, most of them are limited to one-step coupling or annulation and just a single ring is formed [15–22]. Therefore, it is necessary to explore a new cascade annulation of acrylamides to construct a polyfused-heteroarene skeleton in one operational step.

**Figure 1:**
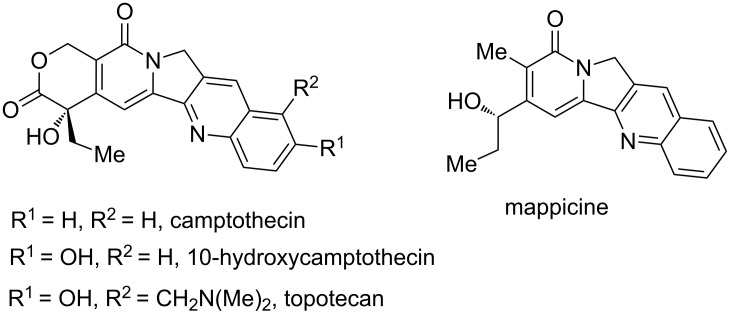
Selected examples of natural products with a tricyclic indolizinone scaffold.

**Scheme 1:**
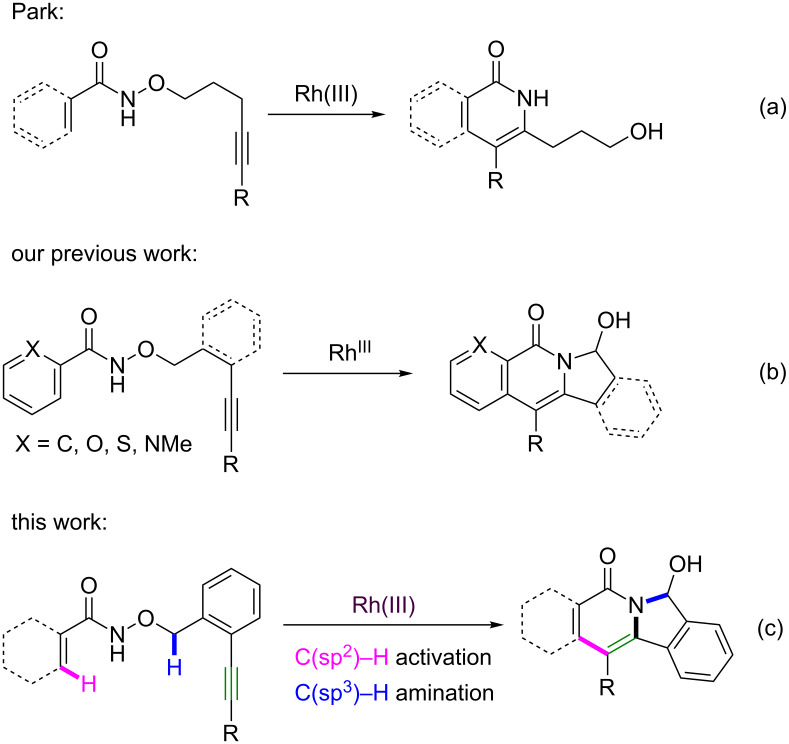
Previous work and this approach.

The tricyclic indolizinone scaffold is abundantly present in natural products, as, e.g., in the pharmacologically relevant mappicine [23–24], camptothecin [25–26], 10-hydroxycamptothecin and topotecan [27–28] (Figure 1). In 2012, Park and co-workers reported a Rh^III^-catalyzed intramolecular annulation of alkyne-tethered hydroxamic esters for the synthesis of isoquinolones and pyridines without using external oxidants (Scheme 1a) [22]. Recently, we reported an intramolecular annulation of benzamides to synthesize indolizinones through Rh^III^-catalyzed C(sp^2^)–H activation (Scheme 1b) [29–31]. Inspired by this work, we envisaged that tricyclic indolizinones could be built through rhodium(III)-catalyzed sequential C(sp^2^)–H activation and C(sp^3^)–H amination of *O*-substituted *N*-hydroxyacrylamides (Scheme 1c).

## Results and Discussion

We selected *N*-hydroxyacrylamide **1a** as our model substrate under standard conditions. In the presence of [RhCp*Cl_2_]_2_ (5 mol %) and CsOAc (2 equiv) in 1,4-dioxane (0.1 M) at 60 °C under air, the desired product **3a** was obtained in 40% yield, together with **2a** in 34% yield (Table 1, entry 1). Other solvents could not improve the yield of **3a** (Table 1, entries 2 and 3). [Ru(*p*-cymene)Cl_2_]_2_ resulted in a very poor yield of **3a** (Table 1, entry 4). Alternative rhodium catalyst [RhCp*(CH_3_CN)_3_](SbF_6_)_2_ gave 17% **2a** and 35% **3a** (Table 1, entry 5). Without CsOAc under [RhCp*(CH_3_CN)_3_](SbF_6_)_2_ catalysis, just 29% **2a** was isolated (Table 1, entry 6). With CsOPiv instead of CsOAc, the products **3a** and **2a** were obtained in 32% and 21% yields, respectively (Table 1, entry 7). Without adding CsOAc, no products were formed (Table 1, entry 8). Also, when the reaction was treated under standard conditions for 0.5 h, the products **3a** and **2a** were isolated in 14% and 12% yields, respectively (Table 1, entry 9), which suggested that **3a** was formed as soon as the reaction was performed.

**Table 1:** Optimization of the reaction conditions.^a^

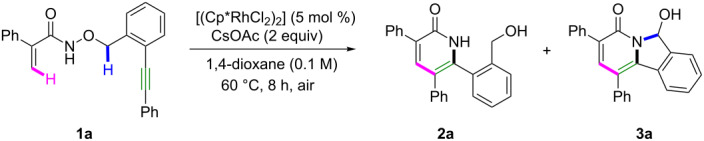		

Entry	Changes to standard conditions	Yields (**2a**, **3a**)^b^

**1**	**none**	**(34%, 40%)**
2	MeOH	(72%, 11%)
3	DCE	(49%, 30%)
4	[Ru(*p*-cymene)Cl_2_]_2_	(52%, 10%)
5	[RhCp*(CH_3_CN)_3_](SbF_6_)_2_	(17%, 35%)
6^c^	[RhCp*(CH_3_CN)_3_](SbF_6_)_2_	(29%, 0%)
7	CsOPiv instead of CsOAc	(21%, 32%)
8	without CsOAc	(0%, 0%)
9	0.5 h instead of 8 h	(12%, 14%)

^a^Conditions: **1a** (0.3 mmol), catalyst (0.015 mmol), CsOAc (0.6 mmol), solvent (3.0 mL). ^b^Isolated yield. ^c^Without CsOAc.

**Scheme 2:**
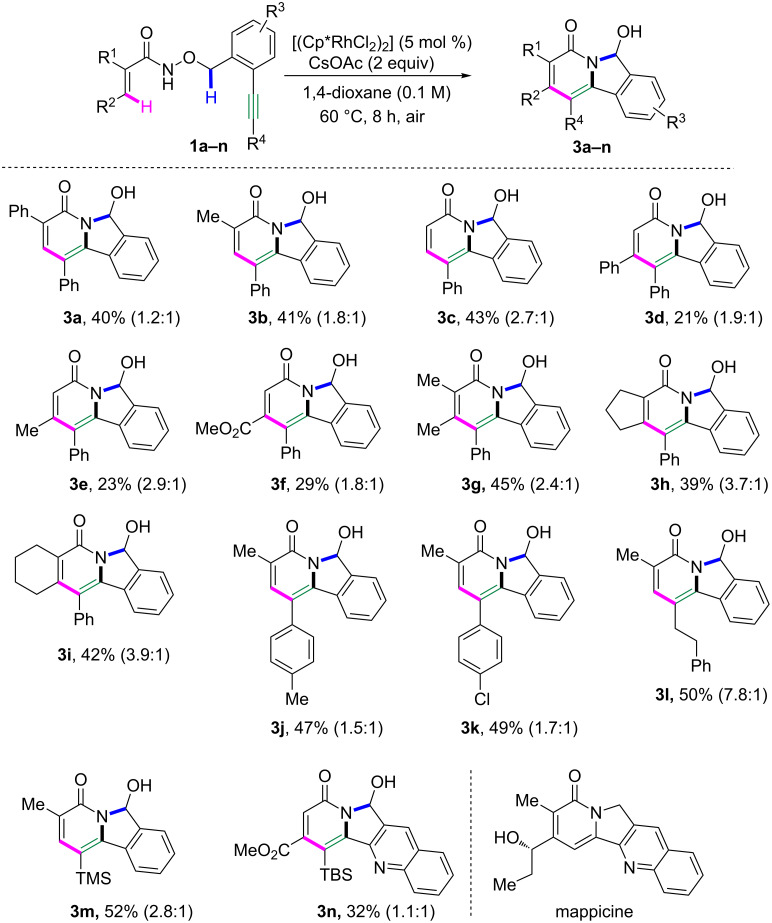
Reaction scope. Reaction conditions: **1** (0.3 mmol), [RhCp*Cl_2_]_2_ (0.015 mmol), CsOAc (0.6 mmol), 1,4-dioxane (3.0 mL), the ratio of isolated **3**:**2** was shown in parenthesis.

Next, diverse substrates were explored to evaluate the scope of this approach under the optimal reaction conditions (Scheme 2). α-Methylacrylamide smoothly proceeded to give the corresponding indolizinone **3b** in 41% yield. Acrylamide afforded the corresponding indolizinone **3c** in 43% yield. Compared to α-substituted acrylamides, β-substituted acrylamides performed the reaction with lower yields under the same conditions (**3d**–**f**). It should be pointed out that α,β-disubstituted acrylamides were also suitable substrates for this transformation, and the corresponding indolizinones **3g**–**i** were obtained in 39–45% yield. Substrates with different substituents on the alkyne, including 4-methylphenyl, 4-chlorophenyl, phenethyl and a TMS group, could deliver the corresponding indolizinones **3j**–**m** in 47–52% yield. Interestingly, 2-ethynylquinoline as substrate worked well, yielding the corresponding indolizinone **3n** in 32%, which has the same skeleton as mappicine.

**Scheme 3:**
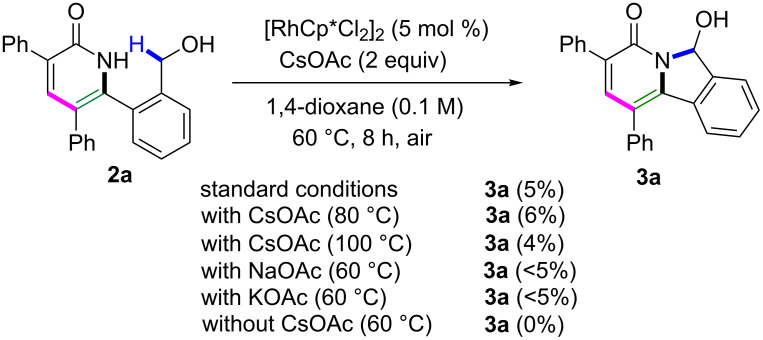
Control experiments.

To investigate the mechanism of this method, control experiments were carried out (Scheme 3). When **2a** was performed under standard conditions, **3a** could be obtained in 5% yield. Increasing the temperature to 80 °C or 100 °C has no dramatic effect on the yield of **3a**. Other bases, like NaOAc or KOAc, could not improve the yield of **3a** from **2a**. On the contrary, **2a** did not give **3a** in the absence of CsOAc. These results indicate that **3a** could be formed through two pathways, and the one from **2a** is the minor pathway. The main pathway is directly from **1a**.

**Scheme 4:**
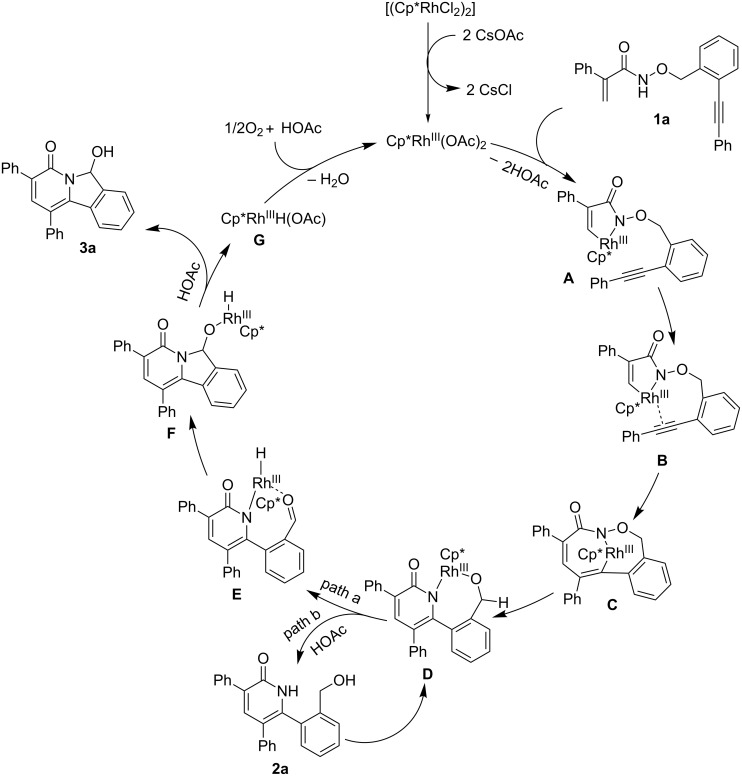
Proposed mechanism.

Based on the above results, a plausible mechanism is proposed in Scheme 4 [31]. C(sp^2^)–H activation of acrylamide **1a**, followed by subsequent intramolecular coordination of the alkyne gives intermediate **B**. Subsequent intramolecular migratory insertion affords intermediate **C**. Reductive elimination and subsequent oxidative addition give intermediate **D**. Then two pathways are involved in the following steps. In the main pathway (path a), intermediate **D** undergoes β-H elimination and tandem cyclization to give product **3a** and Rh–H intermediate **G**, which could be oxidized by O_2_ to regenerate the catalyst. In the minor pathway (path b), intermediate **D** undergoes protonation by acetic acid to give product **2a**, which undergoes deprotonation to form intermediate **D** again, then following the main pathway to give product **3a**.

## Conclusion

In summary, we have developed a rhodium(III)-catalyzed sequential C(sp^2^)–H activation and C(sp^3^)–H amination of *O*-substituted *N*-hydroxyacrylamides for the synthesis of indolizinones. This method shows excellent functional-group tolerance. The family of indolizinone products represents potential bioactive molecules for further studies.

## Supporting Information

File 1 Experimental details and characterization data.
