# Supplementary material for: Intramolecular cascade annulation triggered by rhodium(III)-catalyzed sequential C(sp2)–H activation and C(sp3)–H amination
Source: Beilstein J Org Chem. 2019 Feb 27;15:571–6. doi: 10.3762/bjoc.15.52 (PMC6404421; doi:10.3762/bjoc.15.52)
Supplement: File 1 — Experimental details and characterization data. [file Beilstein_J_Org_Chem-15-571-s001.pdf]

BEILSTEIN JOURNAL OF ORGANIC CHEMISTRY

Supporting Information

for

Intramolecular cascade annulation triggered by rhodium(III)-catalyzed sequential C(sp^2)–H activation and C(sp^3)–H amination

Liangliang Song, Guilong Tian, Johan Van der Eycken and Erik V. Van der Eycken

Beilstein J. Org. Chem. **2019,** *15,* 571–576. doi:10.3762/bjoc.15.52

Experimental details and characterization data

License and Terms: This is a supporting information file under the terms of the Creative Commons Attribution License (http://creativecommons.org/licenses/by/4.0). Please note that the reuse, redistribution and reproduction in particular requires that the authors and source are credited.
The license is subject to the *Beilstein Journal of Organic Chemistry* terms and conditions: (https://www.beilstein-journals.org/bjoc)

Contents

1. General information S2

2. Synthesis of the substrates S2

3. Rhodium(III)-catalyzed intramolecular annulations S14

4. Control experiments S22

5. References S23

6. NMR spectra S24

1. General information

Commercially available reagents were used without additional purification. Column chromatography was performed with silica gel (70–230 mesh). ^{1}H and ^{13}C NMR spectra were recorded on a Bruker AM (300 or 400 or 600 MHz) spectrometer at ambient temperature using $CDCl_3$ or DMSO-d_6 as solvent. HRMS (ESI) spectrometry data were acquired on a quadrupole orthogonal acceleration time-of-flight mass spectrometer [Synapt G2 high definition mass spectrometer (HDMS), Waters, Milford, MA]. Samples were infused at 3 μL min^{-1}, and spectra were obtained in the positive ionization mode with a resolution of 15000 [full width at half maximum (FWHM)] with leucine encephalin as lock mass. Melting points were recorded on a Reichert Thermovar apparatus and are uncorrected.

2. Synthesis of the substrates

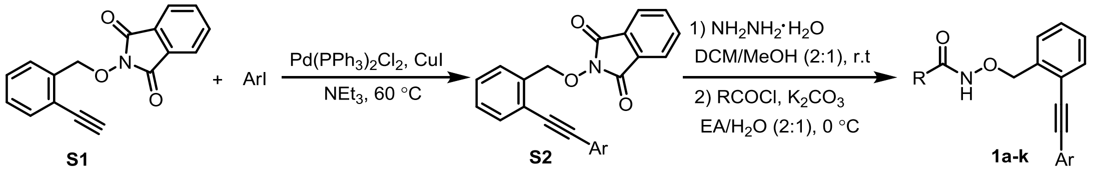

General Procedure A (as described in the literatures[1])

To a 100 mL round-bottom flask,[1] under N_2, was added **S1** (5.0 mmol), $PdCl_2(PPh_3)_2$ (0.25 mmol), CuI (0.25 mmol) and anhydrous TEA (20 mL). The mixture was stirred at room temperature for 1 min and then ArI (6.0 mmol) was added. The flask was placed in a preheated oil bath (60 °C). The reaction was stirred overnight and then cooled to room temperature and checked by TLC. The reaction was filtered over celite, washing with dichloromethane. The solvent was removed and the residue purified by flash column chromatography on silica gel (*n*-heptane/ethyl acetate) to afford **S2**.

In a 100 mL round-bottom flask was charged **S2** (3.0 mmol),[1] solvent (15 mL, MeOH/DCM (1:2)), and then slowly added hydrazine monohydrate (3.3 mmol), then stirred at room temperature for 4 h. Upon completion (indicated by TLC), the solvent

was then removed under reduce pressure. The residue was washed with DCM and filtered, collected the DCM part and removed the solvent to give the crude *O*-alkoxylamine, which was used in the next step without further purification.

The crude *O*-alkoxylamine which was obtained in the previous step was added to a biphasic mixture of K_2CO_3 (3.6 mmol) in solvent (15 mL, EA/H_2O (2:1)).[1] The resulting solution was cooled to 0 °C followed by dropwise addition of the acid chloride (3.3 mmol) dissolved in a minimum amount of EtOAc. The reaction was allowed to stir at the same temperature for 6 h. Upon completion (indicated by TLC), the phases were separated and the aqueous phase was extracted twice with EtOAc. The combined organic layers were dried over Na_2SO_4, filtered, and evaporated under reduced pressure. The residue was purified by flash column chromatography on silica gel (*n*-heptane/ethyl acetate) to give the product **1a**–**k**.

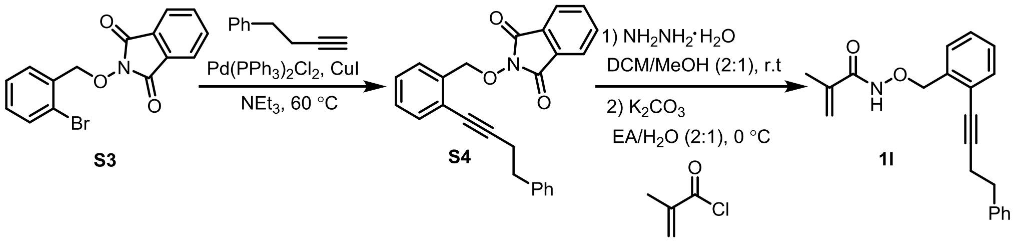

General Procedure B (as described in the literatures[1])

To a 100 mL round-bottom flask, under N_2,[1] was added **S3** (5.0 mmol), $PdCl_2(PPh_3)_2$ (0.25 mmol), CuI (0.25 mmol) and anhydrous TEA (20 mL). The mixture was stirred at room temperature for 1 min and then alkyne (6.0 mmol) was added. The flask was placed in a pre-heated oil bath (60 °C). The reaction was stirred overnight and then cooled to room temperature and checked by TLC. The reaction was filtered over celite, washing with dichloromethane. The solvent was removed and the residue purified by flash column chromatography on silica gel (*n*-heptane/ethyl acetate) to afford **S4**.

In a 100 mL round-bottom flask was charged **S4** (3.0 mmol), solvent 15 mL [MeOH/DCM (1:2)],[1] and then slowly added hydrazine monohydrate (3.3 mmol), then stirred at room temperature for 4 h. Upon completion (indicated by TLC), the solvent was then removed under reduce pressure. The residue was washed with DCM and

filtered, collected the DCM part and removed the solvent to give the crude *O*-alkoxylamine, which was used in the next step without further purification.

The crude *O*-alkoxylamine which was obtained in the previous step was added to a biphasic mixture of K_2CO_3 (3.6 mmol) in solvent (15 mL, EA/H_2O (2:1)).[1] The resulting solution was cooled to 0 °C followed by dropwise addition of the acid chloride (3.3 mmol) dissolved in a minimum amount of EtOAc. The reaction was allowed to stir at the same temperature for 6 h. Upon completion (indicated by TLC), the phases were separated and the aqueous phase was extracted twice with EtOAc. The combined organic layers were dried over Na_2SO_4, filtered, and evaporated under reduced pressure. The residue was purified by flash column chromatography on silica gel (*n*-heptane/ethyl acetate) to give the product **1l**.

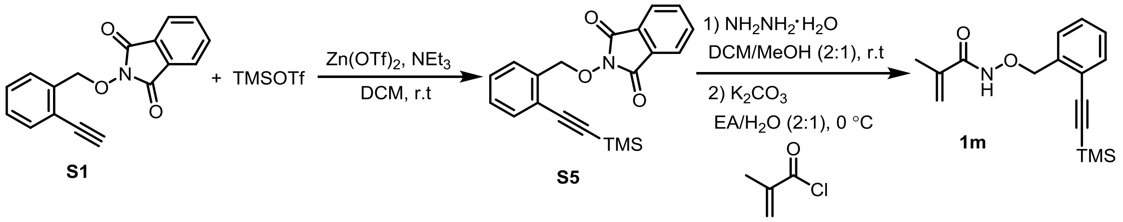

General Procedure C (as described in the literatures[1])

A 50 mL round-bottom flask was charged with zinc triflate (0.25 mmol) and sealed with a septum under an atmosphere of argon.[1] An argon inlet was attached followed by the sequential addition of dry DCM (20 mL), dry triethylamine (7.5 mmol), **S1** (5.0 mmol), and TMSOTf (8.0 mmol). The reaction was stirred until complete as judged by TLC then quenched with saturated NH_4Cl. The mixture was extracted with ether; the aqueous layer was back extracted with ether, and the combined organics were dried, filtered, and concentrated. The crude material was purified by flash column chromatography on silica gel (*n*-heptane/ethyl acetate) to give **S5**.

In a 100 mL round-bottom flask was charged **S5** (3.0 mmol),[1] solvent (15 mL, MeOH/DCM (1:2)), and then slowly added hydrazine monohydrate (3.3 mmol), then stirred at room temperature for 4 h. Upon completion (indicated by TLC), the solvent was then removed under reduce pressure. The residue was washed with DCM and

filtered, collected the DCM part and removed the solvent to give the crude *O*-alkoxylamine, which was used in the next step without further purification.

The crude *O*-alkoxylamine which was obtained in the previous step was added to a biphasic mixture of K_2CO_3 (3.6 mmol) in solvent (15 mL, EA/H_2O (2:1)).[1] The resulting solution was cooled to 0 °C followed by dropwise addition of the acid chloride (3.3 mmol) dissolved in a minimum amount of EtOAc. The reaction was allowed to stir at the same temperature for 6 h. Upon completion (indicated by TLC), the phases were separated and the aqueous phase was extracted twice with EtOAc. The combined organic layers were dried over Na_2SO_4, filtered, and evaporated under reduced pressure. The residue was purified by flash column chromatography on silica gel (*n*-heptane/ethyl acetate) to give the product **1m**.

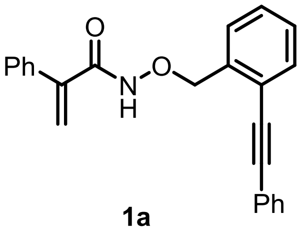

1a

Following **General Procedure A**, **1a** was obtained as a yellow solid (41%, three steps). Melting point 88-90 °C.

^{1}H NMR (400 MHz, $CDCl_3$) δ 8.22 (1 H, d, J = 10.5 Hz, NH), 7.59-7.49 (4 H, m), 7.35-7.26 (10 H, m), 5.98 (1 H, s), 5.59 (1 H, s), 5.27 (2 H, s, CH_2).

^{13}C NMR (101 MHz, $CDCl_3$) δ 165.5, 142.1, 136.7, 135.8, 132.3, 131.6, 130.0, 128.7, 128.6, 128.6, 128.5, 128.3, 127.6, 123.7, 122.8, 122.2, 94.1, 86.8, 76.2.

HRMS (ESI, m/z) calcd for $C_{24}H_{20}NO_2$ $(M+H)^+$: 354.1489, found: 354.1490.

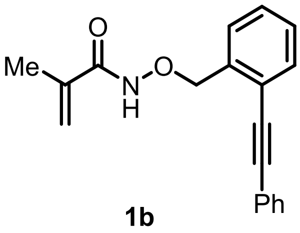

1b

Following **General Procedure A**, **1b** was obtained as a yellow oil (35%, three steps).

^{1}H NMR (400 MHz, $CDCl_3$) δ 8.45 (1 H, s, NH), 7.58-7.51 (4 H, m), 7.34 (5 H, d, J = 2.2 Hz), 5.56 (1 H, s), 5.26 (1 H, s), 5.23 (2 H, s, CH_2), 1.86 (3 H, s, CH_3).

^{13}C NMR (75 MHz, $CDCl_3$) δ 167.1, 138.0, 137.1, 132.3, 131.7, 129.9, 128.5, 128.4, 123.8, 123.0, 120.1, 94.1, 86.9, 76.3, 18.3.

HRMS (ESI, m/z) calcd for $C_{19}H_{18}NO_2$ $(M+H)^+$: 292.1332, found: 292.1327.

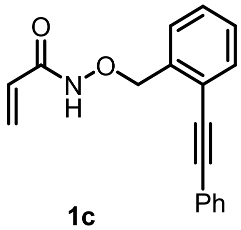

1c

Following **General Procedure A**, **1c** was obtained as a yellow solid (37%, three steps). Melting point 81-83 °C.

^{1}H NMR (400 MHz, $CDCl_3$) δ 8.95 (1 H, s, NH), 7.51 (4 H, dd, J = 12.7, 9.3 Hz), 7.32 (5 H, s), 6.31 (1 H, d, J = 16.8 Hz), 6.15-5.82 (1 H, m), 5.58 (1 H, d, J = 10.0 Hz), 5.19 (2 H, s, CH_2).

^{13}C NMR (101 MHz, $CDCl_3$) δ 163.9, 136.8, 132.3, 131.6, 129.8, 128.5, 128.3, 123.5, 122.8, 94.0, 86.7, 76.2.

HRMS (ESI, m/z) calcd for $C_{18}H_{16}NO_2$ $(M+H)^+$: 278.1176, found: 278.1181.

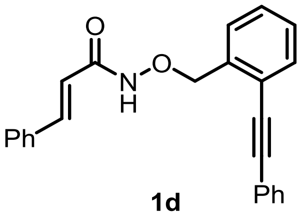

1d

Following **General Procedure A**, **1d** was obtained as a yellow solid (33%, three steps). Melting point 82-84 °C.

^{1}H NMR (400 MHz, $CDCl_3$) δ 7.61 (1 H, d, J = 15.8 Hz), 7.49 (4 H, d, J = 2.9 Hz), 7.36 (2 H, s), 7.24 (9 H, d, J = 2.7 Hz), 5.23 (2 H, s, CH_2).

^{13}C NMR (75 MHz, $CDCl_3$) δ 171.2, 156.5, 134.5, 132.1, 131.5, 129.6, 128.5, 128.4, 128.2, 127.8, 122.6, 94.0, 86.7, 69.7.

HRMS (ESI, m/z) calcd for $C_{24}H_{20}NO_2$ (M+H) $^+$: 354.1489, found: 354.1490.

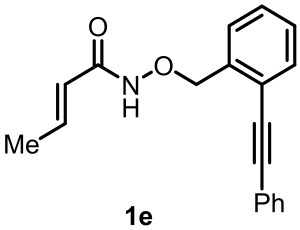

1e

Following **General Procedure A**, **1e** was obtained as a yellow solid (31%, three steps). Melting point 109-111 °C.

^1H NMR (400 MHz, $CDCl_3$) δ 8.20 (1 H, s, NH), 7.60-7.51 (3 H, m), 7.48 (1 H, d, J = 4.4 Hz), 7.40-7.30 (5 H, m), 6.91 (1 H, dd, J = 14.4, 6.9 Hz), 5.18 (2 H, s, CH_2), 1.76 (3 H, dd, J = 6.9, 1.7 Hz, CH_3).

^{13}C NMR (101 MHz, $CDCl_3$) δ 132.4, 131.6, 129.9, 128.7, 128.5, 128.3, 123.7, 122.8, 94.0, 86.8, 77.2, 18.0.

HRMS (ESI, m/z) calcd for $C_{19}H_{18}NO_2$ (M+H) $^+$: 292.1332, found: 292.1326.

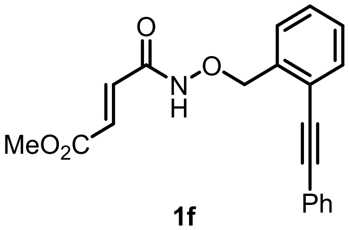

1f

Following **General Procedure A**, **1f** was obtained as a yellow solid (27%, three steps). Melting point 87-89 °C.

^1H NMR (300 MHz, $CDCl_3$) δ 7.53-7.42 (4 H, m), 7.30 (5 H, s), 6.79 (2 H, dd, J = 37.2, 14.2 Hz), 5.24 (2 H, s, CH_2), 3.64 (3 H, s, CH_3).

^{13}C NMR (151 MHz, $CDCl_3$) δ 165.9, 161.6, 136.6, 133.2, 132.1, 131.5, 130.2, 129.8, 128.4, 128.2, 123.4, 122.7, 94.0, 86.7, 76.0, 52.0.

HRMS (ESI, m/z) calcd for $C_{20}H_{18}NO_4$ (M+H) $^+$: 336.1230, found: 336.1236.

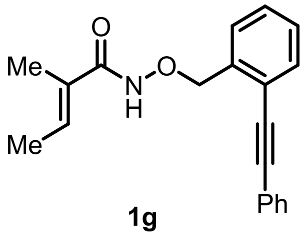

1g

Following **General Procedure A**, **1g** was obtained as a yellow oil (39%, three steps).

^{1}H NMR (400 MHz, $CDCl_3$) δ 7.59-7.49 (4 H, m), 7.39-7.31 (5 H, m), 6.29 (1 H, qd, J = 6.9, 1.3 Hz), 5.22 (2 H, s, CH_2), 1.74 (3 H, s, CH_3), 1.64 (3 H, dd, J = 6.9, 0.7 Hz, CH_3).

^{13}C NMR (101 MHz, $CDCl_3$) δ 137.2, 132.2, 131.6, 129.9, 128.5, 128.5, 128.3, 123.5, 122.8, 93.9, 86.9, 76.0, 13.7, 12.0.

HRMS (ESI, m/z) calcd for $C_{20}H_{20}NO_2$ $(M+H)^+$: 306.1489, found: 306.1489.

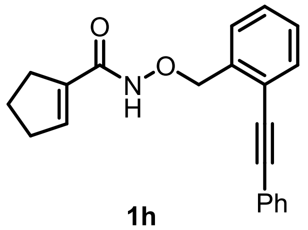

1h

Following **General Procedure A**, **1h** was obtained as a yellow solid (36%, three steps). Melting point 83-85 °C.

^{1}H NMR (600 MHz, $CDCl_3$) δ 8.28 (1 H, s, NH), 7.60 (1 H, dd, J = 7.1, 1.8 Hz), 7.57-7.53 (3 H, m), 7.39-7.35 (5 H, m), 6.50 (1 H, s), 5.25 (2 H, s, OCH_2Ar), 2.48-2.42 (2 H, m, CH_2-alkene), 2.41-2.36 (2 H, m, CH_2-alkene), 1.92-1.86 (2 H, m, CH_2).

^{13}C NMR (151 MHz, $CDCl_3$) δ 164.2, 139.7, 137.0, 136.1, 132.3, 131.6, 130.0, 128.6, 128.5, 128.5, 128.3, 123.6, 122.8, 93.9, 86.9, 76.4, 33.0, 31.2, 23.0.

HRMS (ESI, m/z) calcd for $C_{21}H_{20}NO_2$ $(M+H)^+$: 318.1489, found: 318.1487.

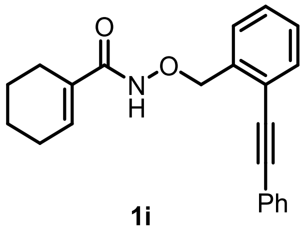

1i

Following **General Procedure A**, **1i** was obtained as a yellow solid (42%, three steps). Melting point 91-93 °C.

^{1}H NMR (400 MHz, $CDCl_3$) δ 8.42 (1 H, s, NH), 7.58-7.48 (4 H, m), 7.37-7.30 (5 H, m), 6.53-6.44 (1 H, m), 5.20 (2 H, s, OCH_2Ar), 2.11 (2 H, d, J = 1.4 Hz, CH_2-alkene), 2.01 (2 H, d, J = 2.8 Hz, CH_2-alkene), 1.52 (4 H, m, CH_2CH_2).

^{13}C NMR (101 MHz, $CDCl_3$) δ 167.3, 137.1, 134.5, 132.1, 131.5, 130.9, 129.7, 128.4, 128.3, 128.2, 123.3, 122.8, 93.8, 86.8, 76.0, 25.1, 23.7, 21.7, 21.2.

HRMS (ESI, m/z) calcd for $C_{22}H_{22}NO_2$ $(M+H)^+$: 332.1645, found: 332.1640.

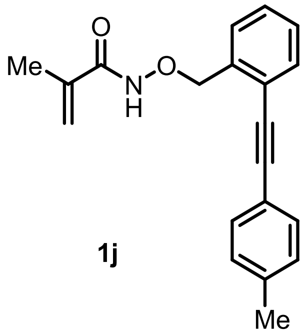

1j

Following **General Procedure A**, **1j** was obtained as a yellow oil (36%, three steps).

^{1}H NMR (300 MHz, $CDCl_3$) δ 9.17 (1 H, s, NH), 7.52-7.47 (2 H, m), 7.40 (2 H, d, J = 8.1 Hz), 7.29-7.24 (2 H, m), 7.09 (2 H, d, J = 7.9 Hz), 5.58 (1 H, s), 5.22-5.19 (1 H, m), 5.18 (2 H, s, CH_2), 2.30 (3 H, s, CH_3Ar), 1.82 (3 H, s, CH_3-alkene).

^{13}C NMR (101 MHz, $CDCl_3$) δ 166.8, 138.3, 137.3, 136.7, 131.8, 131.2, 129.4, 128.8, 128.1, 128.0, 123.1, 120.3, 119.6, 93.9, 86.0, 75.7, 21.2, 18.1.

HRMS (ESI, m/z) calcd for $C_{20}H_{20}NO_2$ $(M+H)^+$: 306.1489, found: 306.1489.

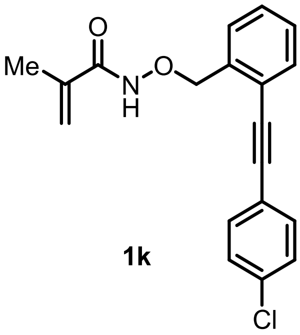

1k

Following **General Procedure A**, **1k** was obtained as a yellow oil (32%, three steps).

^{1}H NMR (300 MHz, $CDCl_3$) δ 8.88 (1 H, s, NH), 7.55-7.48 (2 H, m), 7.46-7.42 (2 H, m), 7.34-7.30 (2 H, m), 7.29-7.26 (2 H, m), 5.57 (1 H, s), 5.24 (1 H, d, J = 1.2 Hz), 5.18 (2 H, s, CH_2), 1.84 (3 H, s, CH_3).

^{13}C NMR (101 MHz, $CDCl_3$) δ 167.0, 137.5, 136.9, 134.3, 132.7, 132.1, 129.8, 128.6, 128.5, 128.4, 123.1, 121.2, 120.4, 92.6, 87.7, 75.7, 18.2.

HRMS (ESI, m/z) calcd for $C_{19}H_{17}ClNO_2$ (M+H) $^+$: 326.0942, found: 326.0943.

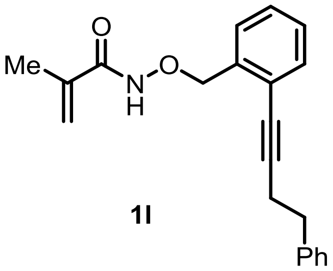

1l

Following **General Procedure B**, **1l** was obtained as a yellow oil (29%, three steps).

^{1}H NMR (600 MHz, $CDCl_3$) δ 8.72 (1 H, s, NH), 7.45-7.43 (1 H, m), 7.37 (1 H, dd, J = 7.4, 1.5 Hz), 7.28 (2 H, t, J = 7.5 Hz), 7.24-7.22 (3 H, m), 7.21-7.17 (2 H, m), 5.56 (1 H, s), 5.25-5.23 (1 H, m), 5.02 (2 H, s, OCH_2Ar), 2.88 (2 H, t, J = 7.4 Hz, CH_2CH_2), 2.69 (2 H, t, J = 7.4 Hz, CH_2CH_2), 1.86 (3 H, s, CH_3).

^{13}C NMR (151 MHz, $CDCl_3$) δ 166.7, 140.4, 137.5, 136.7, 132.0, 129.3, 128.3, 128.2, 128.1, 127.7, 126.1, 123.7, 120.2, 94.1, 78.7, 75.7, 34.7, 21.4, 18.2.

HRMS (ESI, m/z) calcd for $C_{21}H_{22}NO_2$ (M+H) $^+$: 320.1645, found: 320.1651.

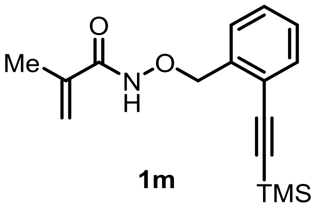

1m

Following **General Procedure C**, **1m** was obtained as a yellow solid (33%, three steps). Melting point 76-78 °C.

^{1}H NMR (600 MHz, $CDCl_3$) δ 7.51 (2 H, dt, J = 14.8, 7.4 Hz), 7.36 (1 H, dd, J = 7.5, 6.6 Hz), 7.33-7.29 (1 H, m), 5.61 (1 H, s), 5.33 (1 H, s), 5.17 (2 H, s, CH_2), 1.93 (3 H, s, CH_3), 0.27 (9 H, d, J = 0.5 Hz, TMS).

^{13}C NMR (151 MHz, $CDCl_3$) δ 137.3, 132.6, 129.7, 128.7, 128.4, 123.4, 120.2, 102.4, 99.3, 76.0, 18.4, -0.1.

HRMS (ESI, m/z) calcd for $C_{16}H_{22}NO_2Si$ $(M+H)^+$: 288.1414, found: 288.1414.

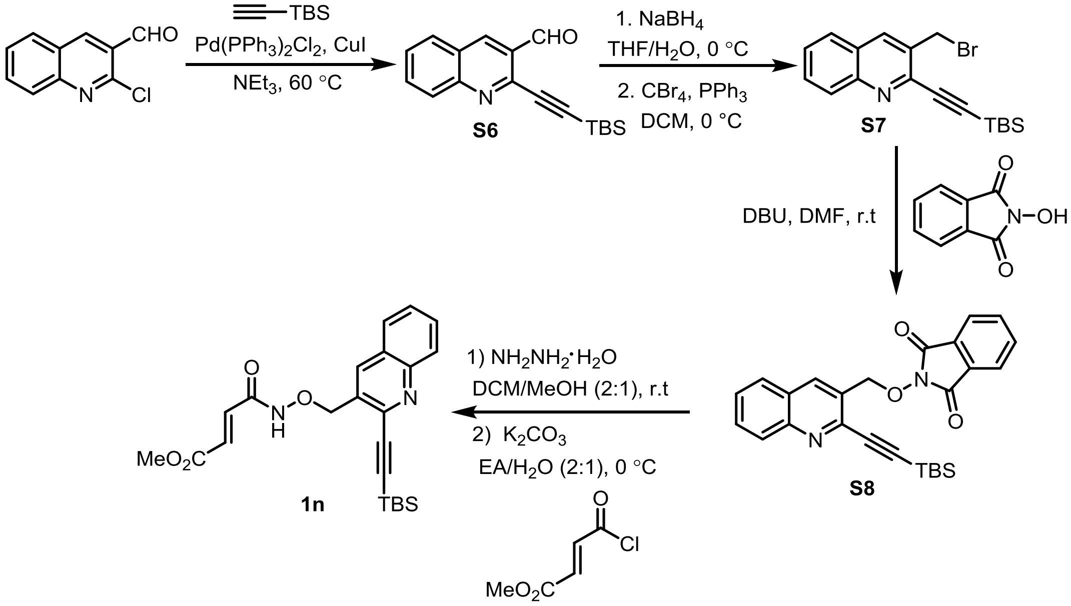

As described in the literatures[1], to a 100 mL round-bottom flask,[1] under N_2, was added 2-chloro-3-quinolinecarboxaldehyde (5.0 mmol), $PdCl_2(PPh_3)_2$ (0.25 mmol), CuI (0.25 mmol) and anhydrous TEA (20 mL). The mixture was stirred at room temperature for 1 min and then (*tert*-butyldimethylsilyl)acetylene (6.0 mmol) was added. The flask was placed in a preheated oil bath (60 °C). The reaction was stirred overnight and then cooled to room temperature and checked by TLC. The reaction was filtered over celite,

and washed with dichloromethane. The solvent was removed and the residue purified by flash column chromatography on silica gel (*n*-heptane/ethyl acetate) to afford **S6**.

A solution of **S6** (4.0 mmol) in THF (10 mL) and water (1 mL) was cooled to 0 °C, $NaBH_4$ (4.8 mmol) was then added.[1] After stirring for a further period of 1 h, the reaction was completed as indicated by TLC. The reaction mixture was extracted twice with DCM. The combined organic layers were dried over Na_2SO_4, filtered, and evaporated under reduced pressure, affording the crude product, which was used in next step without further purification.

To a stirring solution of CBr_4 (7.2 mmol) in DCM (12 mL), a solution of the crude product from the previous step in DCM was added to the resulting mixture at 0 °C.[1,2] After stirring for another 5 minutes, a solution of PPh_3 (8.0 mmol) in DCM was added dropwise at 0 °C. The reaction media was then stirred at 0 °C for 1 h (judging by TLC analysis). The solution was concentrated in vacuo. The obtained crude product was purified by flash column chromatography on silica gel (*n*-heptane/ethyl acetate) to give the desired product **S7**.

To a stirred mixture of *N*-hydroxyphthalimide (3.0 mmol) and **S7** (3.3 mmol) in DMF (6 mL) was added DBU (3.6 mmol) slowly at ambient temperature.[1,3] After completion of the addition, water (20 mL) was added, and the precipitate was filtered off and washed with water to afford **S8**, which was used in next step without further purification.

In a 100 mL round-bottom flask was charged **S8** (3.0 mmol),[1] solvent (15 mL, MeOH/DCM (1:2)), and then slowly added hydrazine monohydrate (3.3 mmol), then stirred at room temperature for 4 h. Upon completion (indicated by TLC), the solvent was then removed under reduce pressure. The residue was washed with DCM and filtered, collected the DCM part and removed the solvent to give the crude *O*-alkoxylamine, which was used in the next step without further purification.

The crude *O*-alkoxylamine which was obtained in the previous step was added to a biphasic mixture of K_2CO_3 (3.6 mmol) in solvent (15 mL, EA/H_2O (2:1)).[1] The resulting solution was cooled to 0 °C followed by dropwise addition of the acid chloride (3.3

mmol) dissolved in a minimum amount of EtOAc. The reaction was allowed to stir at the same temperature for 6 h. Upon completion (indicated by TLC), the phases were separated and the aqueous phase was extracted twice with EtOAc. The combined organic layers were dried over Na_2SO_4, filtered, and evaporated under reduced pressure. The residue was purified by flash column chromatography on silica gel (*n*-heptane/ethyl acetate) to give the product **1n**.

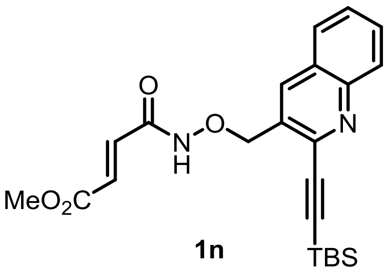

1n

1n was obtained as a yellow oil (79%, last two steps).

^{1}H NMR (300 MHz, $CDCl_3$) δ 8.10 (1 H, s), 8.04 (1 H, d, J = 8.3 Hz), 7.69 (2 H, dd, J = 13.2, 5.0 Hz), 7.51 (1 H, t, J = 7.4 Hz), 6.89 (1 H, d, J = 16.5 Hz), 6.72 (1 H, d, J = 15.5 Hz), 5.36 (2 H, s, CH_2), 3.76 (3 H, s, CH_3), 1.02 (9 H, s, *t*BuSi), 0.23 (6 H, s, CH_3SiCH_3).

^{13}C NMR (101 MHz, $CDCl_3$) δ 165.7, 161.8, 154.8, 147.1, 142.4, 136.6, 133.1, 130.4, 128.5, 127.5, 126.7, 124.4, 101.9, 99.6, 75.0, 52.2, 26.1, 16.6, -4.8.

HRMS (ESI, m/z) calcd for $C_{23}H_{29}N_2O_4Si$ $(M+H)^+$: 425.1891, found: 425.1891.

3. Rhodium(III)-catalyzed intramolecular annulations

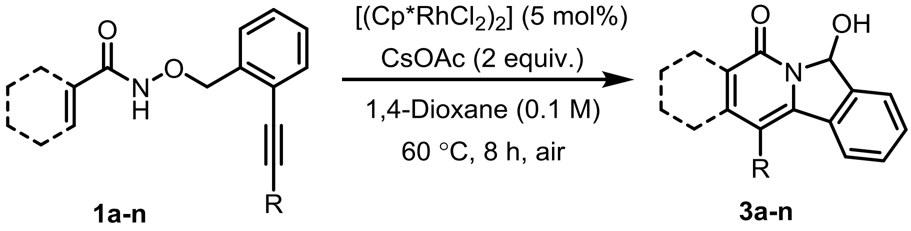

As described in the literatures[1], to a Schlenk flask equipped with a stir bar were added **1a–n** (0.3 mmol), $[Cp^{*}RhCl_2]_2$ (0.015 mmol), CsOAc (0.6 mmol) and 1,4-dioxane (3.0 mL) under air. The reaction was stirred for 8 h at 60 °C, cooled to room temperature. The solvent was removed in vacuo and the remaining residue was purified by a silica gel column chromatography (*n*-heptane/ethyl acetate) to afford the product **3a–n**.

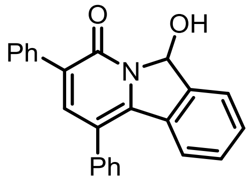

3a

3a was obtained as a yellow solid (40%). Melting point 116-118 °C.

^{1}H NMR (300 MHz, $CDCl_3$) δ 7.84-7.74 (2 H, m), 7.68 (1 H, d, J = 7.6 Hz), 7.61 (1 H, s), 7.53-7.32 (9 H, m), 7.22 (1 H, d, J = 7.2 Hz), 6.99 (1 H, d, J = 7.9 Hz), 6.93 (1 H, s, NCH), 5.67 (1 H, s, OH).

^{13}C NMR (151 MHz, $CDCl_3$) δ 161.1, 142.4, 140.7, 139.3, 136.5, 135.6, 132.5, 130.3, 129.9, 129.6, 129.5, 129.0, 128.6, 128.4, 128.3, 128.0, 124.4, 123.5, 117.8, 85.3.

HRMS (ESI, m/z) calcd for $C_{24}H_{18}NO_2$ $(M+H)^+$: 352.1332, found: 352.1346.

Spectral data was consistent with that previously reported.[4]

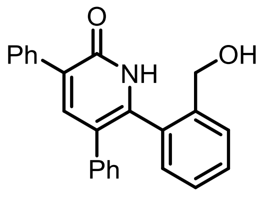

2a

2a was obtained as a yellow solid (34%). Melting point 206-208 °C.

^{1}H NMR (400 MHz, DMSO-d$_6$) δ 11.95 (1 H, s, NH), 7.86 (2 H, d, J = 7.6 Hz), 7.73 (1 H, s), 7.48-7.31 (5 H, m), 7.24-7.09 (7 H, m), 5.21 (1 H, s, OH), 4.31 (2 H, d, J = 71.3 Hz, CH$_2$).

^{13}C NMR (151 MHz, DMSO-d$_6$) δ 141.1, 141.1, 136.9, 130.6, 129.4, 128.8, 128.5, 128.4, 127.9, 127.5, 126.9, 61.1.

HRMS (ESI, m/z) calcd for $C_{24}H_{20}NO_2$ (M+H)$^+$: 354.1489, found: 354.1486.

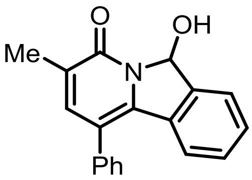

3b

3b was obtained as a dark solid (41%).

^{1}H NMR (400 MHz, CDCl$_3$) δ 7.65 (1 H, d, J = 7.6 Hz), 7.52-7.45 (3 H, m), 7.44-7.36 (3 H, m), 7.29 (1 H, d, J = 1.0 Hz), 7.19 (1 H, t, J = 7.7 Hz), 6.94 (1 H, d, J = 7.9 Hz), 6.87 (1 H, s, NCH), 5.73 (1 H, s, OH), 2.25 (3 H, d, J = 0.7 Hz, CH$_3$).

^{13}C NMR (101 MHz, CDCl$_3$) δ 162.5, 142.0, 139.2, 138.9, 136.7, 132.8, 129.9, 129.7, 129.4, 128.9, 128.2, 128.0, 124.3, 123.2, 117.4, 84.8, 16.0.

Spectral data was consistent with that previously reported.[4]

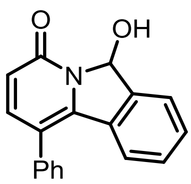

3c

3c was obtained as a yellow solid (43%)

^{1}H NMR (400 MHz, CDCl$_3$) δ 7.67 (1 H, d, J = 7.6 Hz), 7.50 (3 H, tt, J = 8.4, 4.1 Hz), 7.43 (4 H, dd, J = 12.0, 5.0 Hz), 7.21 (1 H, t, J = 7.5 Hz), 6.94 (1 H, d, J = 7.9 Hz), 6.87 (1 H, s), 6.57 (1 H, d, J = 9.2 Hz, NCH), 5.68 (1 H, s, OH).

^{13}C NMR (101 MHz, $CDCl_3$) δ 162.3, 144.6, 141.7, 139.1, 136.4, 132.5, 130.4, 129.8, 129.4, 129.0, 128.4, 124.4, 123.6, 118.6, 117.7, 84.9.

Spectral data was consistent with that previously reported.[4]

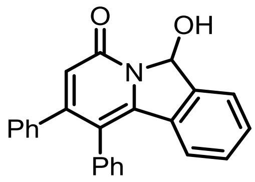

3d

3d was obtained as a yellow solid (21%). Melting point 253-255 °C.

^1H NMR (300 MHz, $CDCl_3$) δ 7.67 (1 H, d, J = 7.5 Hz), 7.41 (1 H, t, J = 7.5 Hz), 7.33 (3 H, s), 7.16 (6 H, dd, J = 12.7, 6.6 Hz), 7.07 (2 H, d, J = 5.8 Hz), 6.91 (1 H, s), 6.59 (1 H, s), 6.44 (1 H, d, J = 7.9 Hz, NCH), 5.68 (1 H, s, OH).

^{13}C NMR (101 MHz, $CDCl_3$) δ 162.0, 156.5, 142.3, 139.4, 138.0, 134.8, 132.9, 131.0, 131.0, 130.3, 129.9, 128.8, 128.7, 128.1, 127.9, 127.8, 124.3, 124.2, 118.7, 84.6.

HRMS (ESI, m/z) calcd for $C_{24}H_{18}NO_2$ $(M+H)^+$: 352.1332, found: 352.1343.

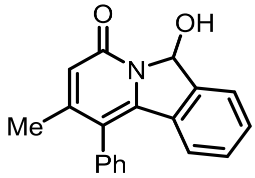

3e

3e was obtained as a yellow solid (23%). Melting point 217-219 °C.

^1H NMR (400 MHz, $CDCl_3$) δ 7.64 (1 H, d, J = 7.6 Hz), 7.56-7.49 (3 H, m), 7.38 (1 H, td, J = 7.6, 0.8 Hz), 7.31-7.25 (2 H, m), 7.12 (1 H, t, J = 7.7 Hz), 6.84 (1 H, s), 6.46 (1 H, s), 6.28 (1 H, d, J = 7.9 Hz, NCH), 5.66 (1 H, s, OH), 1.99 (3 H, d, J = 0.8 Hz, CH_3).

^{13}C NMR (101 MHz, $CDCl_3$) δ 162.2, 153.4, 141.4, 139.4, 135.2, 132.8, 130.1, 130.0, 129.8, 129.4, 128.4, 124.3, 123.8, 118.5, 117.9, 84.4, 20.8.

HRMS (ESI, m/z) calcd for $C_{19}H_{16}NO_2$ $(M+H)^+$: 290.1176, found: 290.1178.

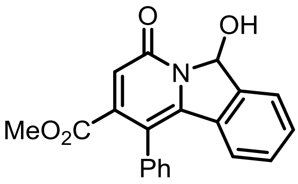

3f

3f was obtained as a yellow solid (29%). Melting point 129-131 °C.

^{1}H NMR (300 MHz, $CDCl_3$) δ 7.66 (1 H, d, J = 7.7 Hz), 7.52-7.47 (3 H, m), 7.43 (1 H, t, J = 7.5 Hz), 7.33 (2 H, dd, J = 9.2, 5.1 Hz), 7.17 (1 H, t, J = 7.7 Hz), 6.87 (1 H, s), 6.85 (1 H, s), 6.39 (1 H, d, J = 7.9 Hz, NCH), 3.60 (3 H, s, CH_3).

^{13}C NMR (101 MHz, $CDCl_3$) δ 166.0, 161.6, 146.1, 143.5, 139.2, 134.3, 132.3, 130.7, 130.1, 130.0, 129.9, 128.9, 128.9, 128.6, 124.3, 119.0, 84.9, 52.5.

HRMS (ESI, m/z) calcd for $C_{20}H_{16}NO_4$ $(M+H)^+$: 334.1074, found: 334.1076.

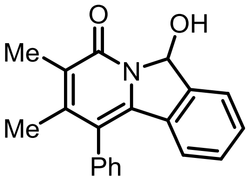

3g

3g was obtained as a yellow solid (45%). Melting point 234-236 °C.

^{1}H NMR (300 MHz, $CDCl_3$) δ 7.62 (1 H, d, J = 7.4 Hz), 7.57-7.47 (3 H, m), 7.37-7.22 (3 H, m), 7.13-7.05 (1 H, m), 6.86 (1 H, d, J = 1.8 Hz), 6.19 (1 H, d, J = 7.9 Hz, NCH), 5.81 (1 H, d, J = 2.7 Hz, OH), 2.23 (3 H, d, J = 0.5 Hz, CH_3), 1.95 (3 H, d, J = 0.5 Hz, CH_3).

^{13}C NMR (101 MHz, $CDCl_3$) δ 162.1, 148.2, 138.9, 138.3, 136.2, 133.2, 130.2, 129.7, 129.5, 129.3, 128.3, 125.1, 124.2, 123.5, 118.7, 84.8, 17.6, 12.4.

HRMS (ESI, m/z) calcd for $C_{20}H_{18}NO_2$ $(M+H)^+$: 304.1332, found: 304.1331.

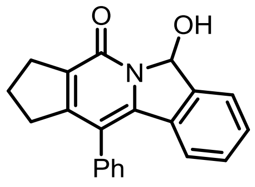

3h

3h was obtained as a yellow solid (39%). Melting point 201-203 °C.

^{1}H NMR (300 MHz, $CDCl_3$) δ 7.63 (1 H, d, J = 7.5 Hz), 7.54-7.45 (3 H, m), 7.39-7.28 (3 H, m), 7.15 (1 H, dd, J = 11.9, 4.2 Hz), 6.85 (1 H, d, J = 2.5 Hz), 6.57 (1 H, d, J = 7.9 Hz, NCH), 5.59 (1 H, d, J = 2.8 Hz, OH), 2.96 (2 H, t, J = 7.5 Hz, CH_2-alkene), 2.63 (2 H, t, J = 7.4 Hz, CH_2-alkene), 2.07 (2 H, p, J = 7.8 Hz, CH_2).

^{13}C NMR (151 MHz, $CDCl_3$) δ 160.5, 157.9, 140.7, 139.3, 135.3, 132.9, 130.9, 129.7, 129.6, 129.1, 129.1, 128.3, 124.3, 123.4, 116.0, 84.4, 33.5, 29.7, 23.5.

HRMS (ESI, m/z) calcd for $C_{21}H_{18}NO_2$ $(M+H)^+$: 316.1332, found: 316.1340.

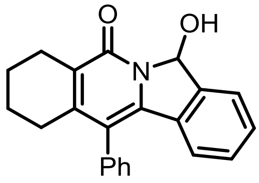

3i

3i was obtained as a yellow solid (42%). Melting point 182-184 °C.

^{1}H NMR (400 MHz, $CDCl_3$) δ 7.61 (1 H, d, J = 7.6 Hz), 7.55-7.48 (3 H, m), 7.33 (1 H, t, J = 7.5 Hz), 7.30-7.27 (1 H, m), 7.24 (1 H, s), 7.09 (1 H, t, J = 7.7 Hz), 6.85 (1 H, s), 6.21 (1 H, d, J = 7.9 Hz, NCH), 5.78 (1 H, s, OH), 2.68 (2 H, t, J = 5.9 Hz, CH_2-alkene), 2.23 (2 H, t, J = 5.3 Hz, CH_2-alkene), 1.75 (2 H, dt, J = 6.1, 4.3 Hz, CH_2CH_2), 1.67 (2 H, dd, J = 12.0, 6.0 Hz, CH_2CH_2).

^{13}C NMR (151 MHz, $CDCl_3$) δ 162.0, 148.9, 138.8, 137.9, 135.4, 133.2, 130.1, 129.6, 129.4, 129.2, 128.2, 126.5, 124.2, 123.4, 118.1, 84.5, 28.3, 23.5, 22.1, 21.7.

HRMS (ESI, m/z) calcd for $C_{22}H_{20}NO_2$ $(M+H)^+$: 330.1489, found: 330.1499.

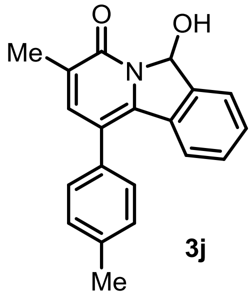

3j

3j was obtained as a white solid (47%). Melting point 223-225 °C.

^{1}H NMR (400 MHz, $CDCl_3$) δ 7.64 (1 H, d, J = 7.6 Hz), 7.39 (1 H, t, J = 7.5 Hz), 7.31-7.26 (5 H, m), 7.20 (1 H, t, J = 7.6 Hz), 6.99 (1 H, d, J = 7.9 Hz), 6.86 (1 H, d, J = 2.6 Hz, NCH), 5.63 (1 H, d, J = 2.9 Hz, OH), 2.46 (3 H, s, CH_3-Ar), 2.24 (3 H, d, J = 0.7 Hz, CH_3-alkene).

^{13}C NMR (101 MHz, $CDCl_3$) δ 162.5, 142.2, 139.1, 138.8, 138.0, 133.7, 132.9, 129.8, 129.7, 129.6, 129.2, 127.9, 124.3, 123.3, 117.4, 84.9, 21.3, 16.0.

HRMS (ESI, m/z) calcd for $C_{20}H_{18}NO_2$ (M+H) $^{+}$: 304.1332, found: 304.1334.

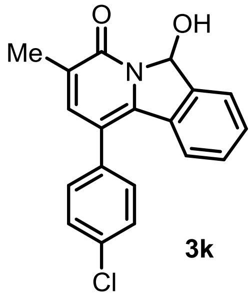

3k

3k was obtained as a yellow solid (49%). Melting point 209-211 °C.

^{1}H NMR (400 MHz, $CDCl_3$) δ 7.66 (1 H, d, J = 7.6 Hz), 7.48 (2 H, d, J = 8.6 Hz), 7.42 (1 H, t, J = 7.5 Hz), 7.35 (2 H, d, J = 7.9 Hz), 7.26 (1 H, s), 7.24 (1 H, s), 6.96 (1 H, d, J = 7.9 Hz), 6.85 (1 H, d, J = 2.5 Hz, NCH), 5.52 (1 H, d, J = 2.8 Hz, OH), 2.24 (3 H, d, J = 0.8 Hz, CH_3).

^{13}C NMR (101 MHz, $CDCl_3$) δ 162.5, 141.6, 139.3, 138.9, 135.2, 134.3, 132.6, 130.8, 130.1, 129.9, 129.2, 128.3, 124.5, 123.1, 116.0, 84.9, 16.0.

HRMS (ESI, m/z) calcd for $C_{19}H_{15}ClNO_2$ (M+H) $^{+}$: 324.0786, found: 324.0801.

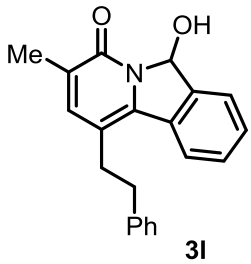

3l

3l was obtained as a yellow solid (50%). Melting point 179-181 °C.

^{1}H NMR (600 MHz, $CDCl_3$) δ 7.78 (1 H, d, J = 7.6 Hz), 7.73 (1 H, d, J = 7.2 Hz), 7.52 (2 H, dt, J = 19.0, 7.4 Hz), 7.35 (2 H, t, J = 7.6 Hz), 7.26 (3 H, t, J = 7.1 Hz), 7.14 (1 H, s), 6.84 (1 H, d, J = 2.3 Hz, NCH), 5.70 (1 H, d, J = 2.8 Hz, OH), 3.13-3.06 (2 H, m, CH_2CH_2), 2.96 (2 H, t, J = 8.1 Hz, CH_2CH_2), 2.20 (3 H, s, CH_3).

^{13}C NMR (151 MHz, $CDCl_3$) δ 162.4, 142.4, 140.6, 139.2, 138.9, 133.1, 130.3, 129.5, 128.6, 128.3, 128.2, 126.4, 124.7, 122.9, 115.8, 84.7, 36.1, 32.4, 15.9.

HRMS (ESI, m/z) calcd for $C_{21}H_{20}NO_2$ $(M+H)^+$: 318.1489, found: 318.1483.

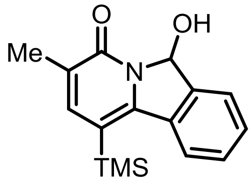

3m

3m was obtained as a yellow solid (52%). Melting point 186-188 °C.

^{1}H NMR (400 MHz, $CDCl_3$) δ 7.86-7.81 (1 H, m), 7.73-7.67 (1 H, m), 7.54-7.46 (2 H, m), 7.44 (1 H, d, J = 0.9 Hz), 6.80 (1 H, d, J = 2.6 Hz, NCH), 5.76 (1 H, d, J = 2.6 Hz, OH), 2.21 (3 H, d, J = 0.8 Hz, CH_3), 0.44 (9 H, s, TMS).

^{13}C NMR (101 MHz, $CDCl_3$) δ 163.0, 148.0, 144.2, 139.5, 133.9, 129.8, 129.6, 127.1, 124.6, 124.0, 110.0, 84.6, 16.0, -0.1.

HRMS (ESI, m/z) calcd for $C_{16}H_{20}NO_2Si$ $(M+H)^+$: 286.1258, found: 286.1252.

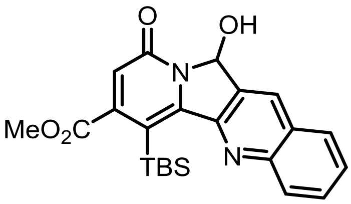

3n

3n was obtained as a yellow solid (32%). Melting point 238-240 °C.

^{1}H NMR (300 MHz, $CDCl_3$) δ 8.46 (1 H, s), 8.29 (1 H, d, J = 8.5 Hz), 7.96 (1 H, d, J = 8.2 Hz), 7.84 (1 H, m), 7.67 (1 H, m), 7.00 (1 H, s), 6.85 (1 H, s, NCH), 5.64 (1 H, d, J = 2.6 Hz, OH), 3.94 (3 H, s, CH_3), 1.26 (9 H, s, *t*BuSi), 0.34 (6 H, d, J = 1.7 Hz, CH_3SiCH_3).

^{13}C NMR (151 MHz, $CDCl_3$) δ 169.0, 161.7, 152.7, 152.3, 150.3, 148.8, 132.2, 130.9, 130.1, 129.3, 128.6, 128.2, 127.7, 121.1, 111.9, 82.6, 52.9, 29.4, 17.9, 0.7, 0.7.

HRMS (ESI, m/z) calcd for $C_{23}H_{27}N_2O_4Si$ $(M+H)^+$: 423.1735, found: 423.1728.

4. Control experiments

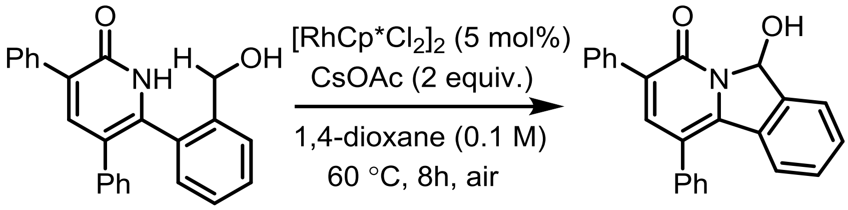

2a 5% **3a**

standard condition	**3a** (5%)
without CsOAc	**3a** (0%)

As described in the literatures[1], to a Schlenk flask equipped with a stir bar were added **2a** (0.3 mmol), [Cp*$RhCl_2$]$_2$ (0.015 mmol), CsOAc (0.6 mmol) and 1,4-dioxane (3.0 mL) under air. The reaction was stirred for 8 h at 60 °C, cooled to room temperature. The solvent was removed in vacuo and the remaining residue was purified by silica gel column chromatography (*n*-heptane/ethyl acetate) to afford the product **3a** (5%).

To a Schlenk flask equipped with a stir bar were added **2a** (0.3 mmol), [Cp*$RhCl_2$]$_2$ (0.015 mmol) and 1,4-dioxane (3.0 mL) under air. The reaction was stirred for 8 h at 60 °C, but no product **3a** was formed.

5. References

1. a) X. Xu, Y. Liu and C. M. Park, *Angew. Chem. Int. Ed.*, 2012, **51**, 9372-9376. b) L. Song, X. Zhang, G. Tian, K. Robeyns, L. Van Meervelt, J. N. Harvey and E. V. Van der Eycken, *Mol. Catal.* **2019**, *463*, 30-36.

2. J. He, Y. Shi, W. Cheng, Z. Man, D. Yang and C.-Y. Li, *Angew. Chem. Int. Ed.*, 2016, **55**, 4557-4561.

3. J. Ozawa, M. Tashiro, J. Ni, K. Oisaki and M. Kanai, *Chem. Sci.*, 2016, **7**, 1904-1909

4. L. Song, G. Tian and E. V. Van der Eycken, *Molecular Catalysis* 2018, **459**, 129-134.

6. NMR spectra

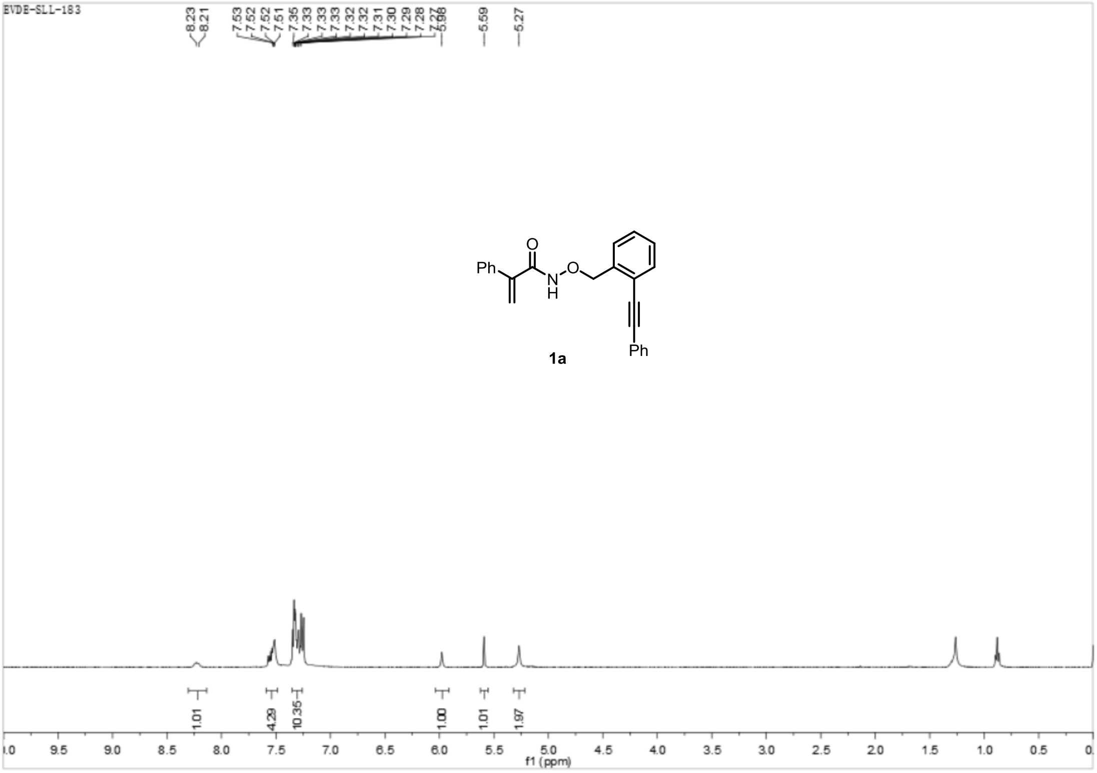

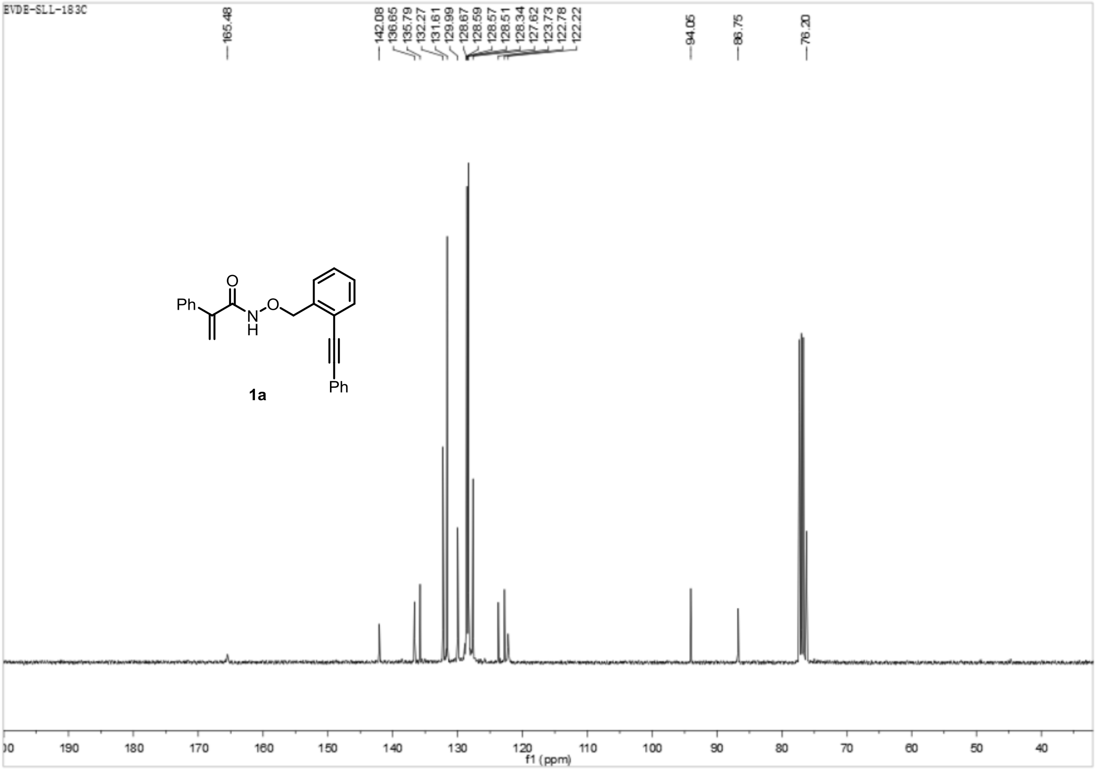

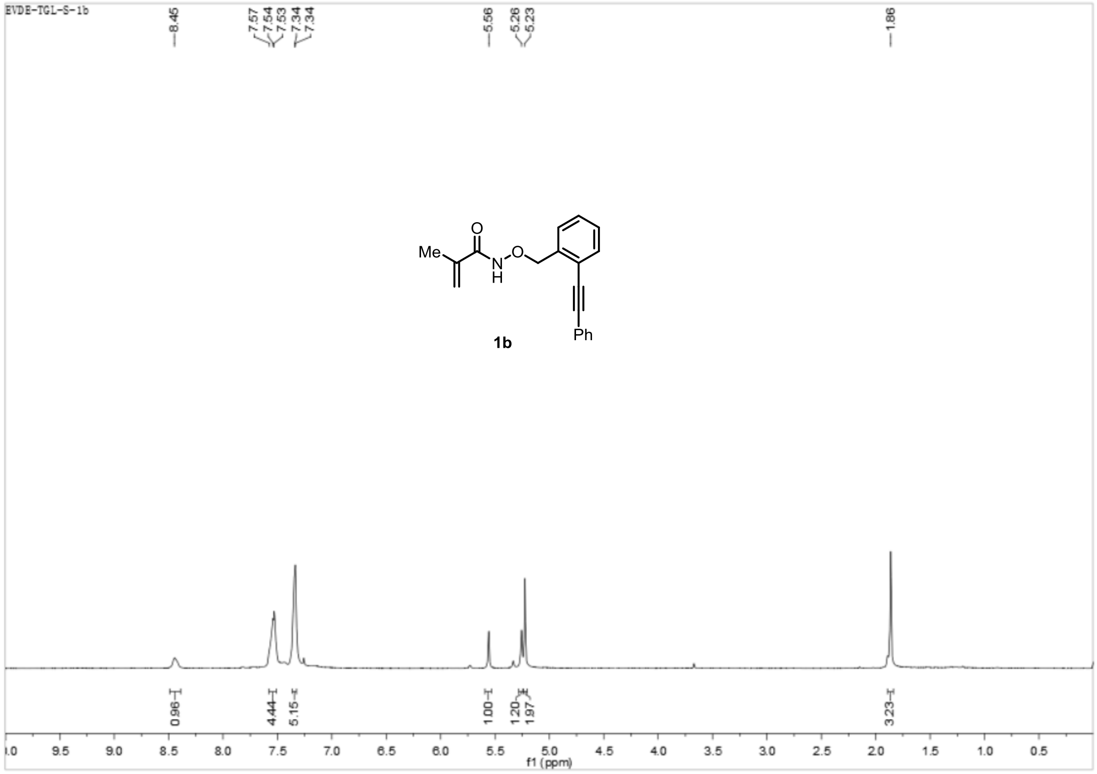
EVDE-TGL-S-1b
8.45
7.57
7.54
7.53
7.34
7.34
5.56
5.26
5.23
1.86
Me
O
N
H
O
Ph
1b
0.96
4.44
5.15
1.00
1.20
1.97
3.23
9.5
9.0
8.5
8.0
7.5
7.0
6.5
6.0
5.5
5.0
4.5
4.0
3.5
3.0
2.5
2.0
1.5
1.0
0.5
f1 (ppm)

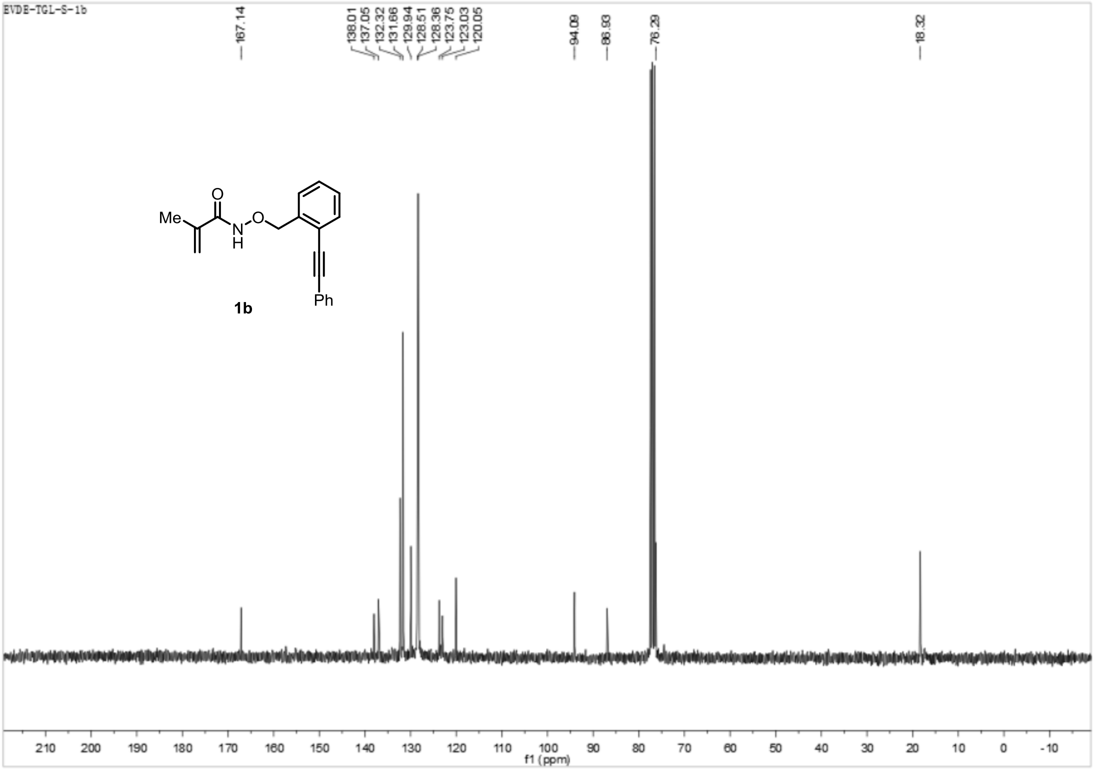
EVDE-TGL-S-1b
167.14
138.01
137.05
132.32
131.66
129.94
128.51
128.36
123.75
123.03
120.05
94.09
86.93
76.29
18.32
Me
O
N
H
O
Ph
1b
210
200
190
180
170
160
150
140
130
120
110
100
90
80
70
60
50
40
30
20
10
0
-10
f1 (ppm)

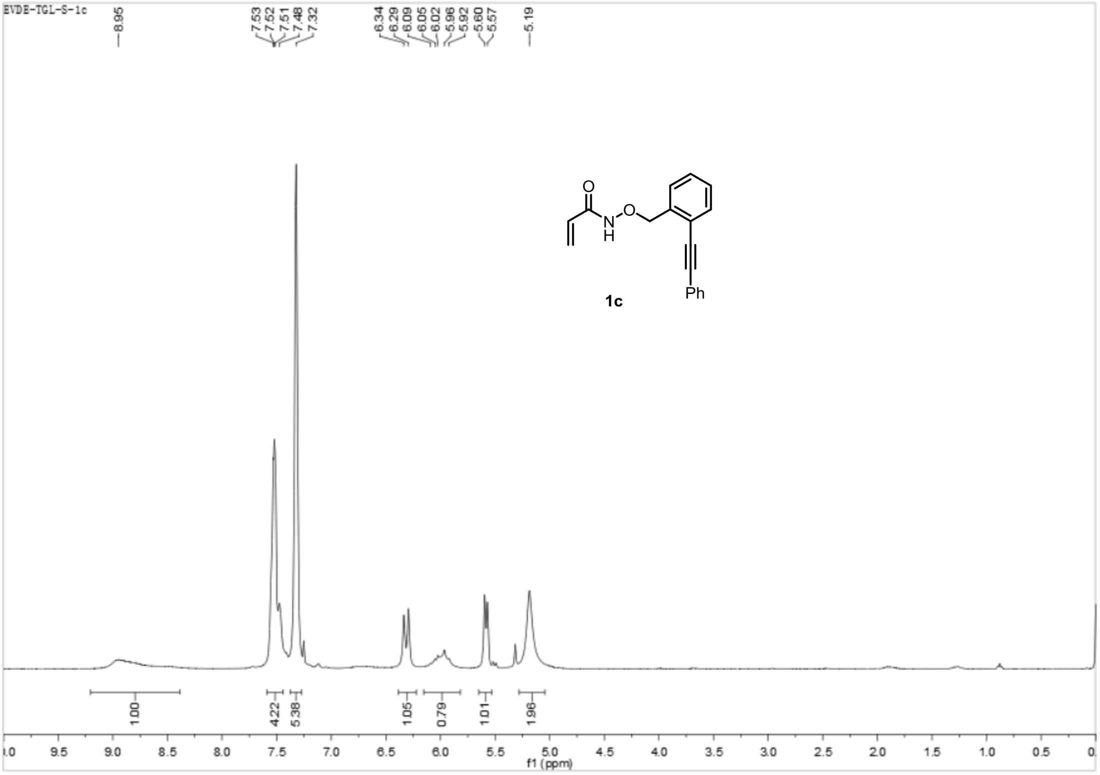
EVDE-TGL-S-1c
8.95
7.53
7.52
7.51
7.48
7.32
6.34
6.29
6.09
6.05
6.02
5.96
5.92
5.60
5.57
5.19
1c
Ph
1.00
4.22
5.38
1.05
0.79
1.01
1.96
f1 (ppm)

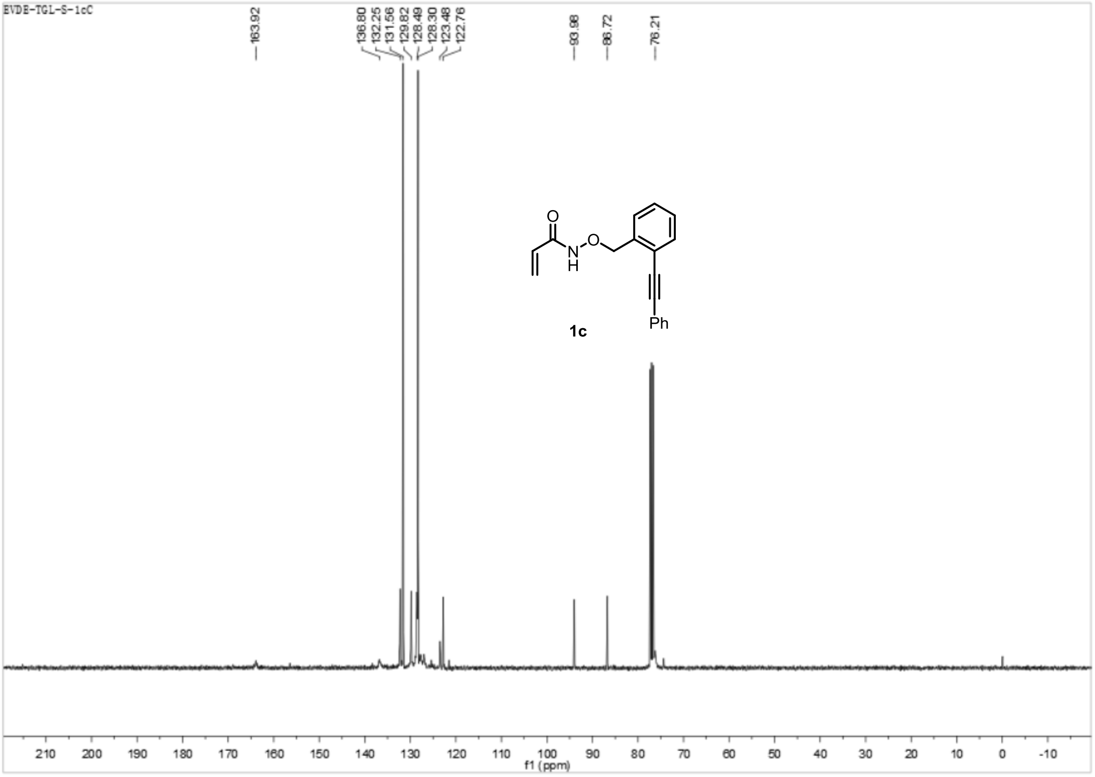
EVDE-TGL-S-1cC
163.92
136.80
132.25
131.56
129.82
128.49
128.30
123.48
122.76
93.98
86.72
76.21
1c
Ph
f1 (ppm)

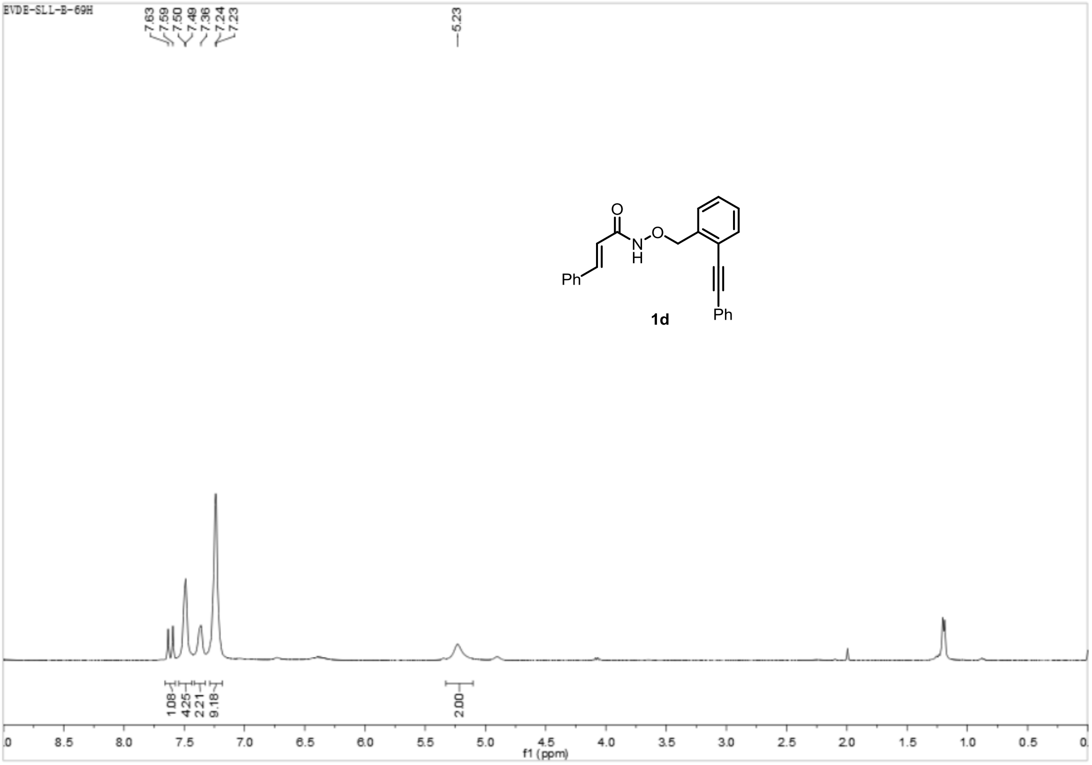
EVDE-SLL-B-69H
7.63
7.59
7.50
7.49
7.36
7.24
7.23
5.23
Ph
1d
1.08
4.25
2.21
9.18
2.00
f1 (ppm)

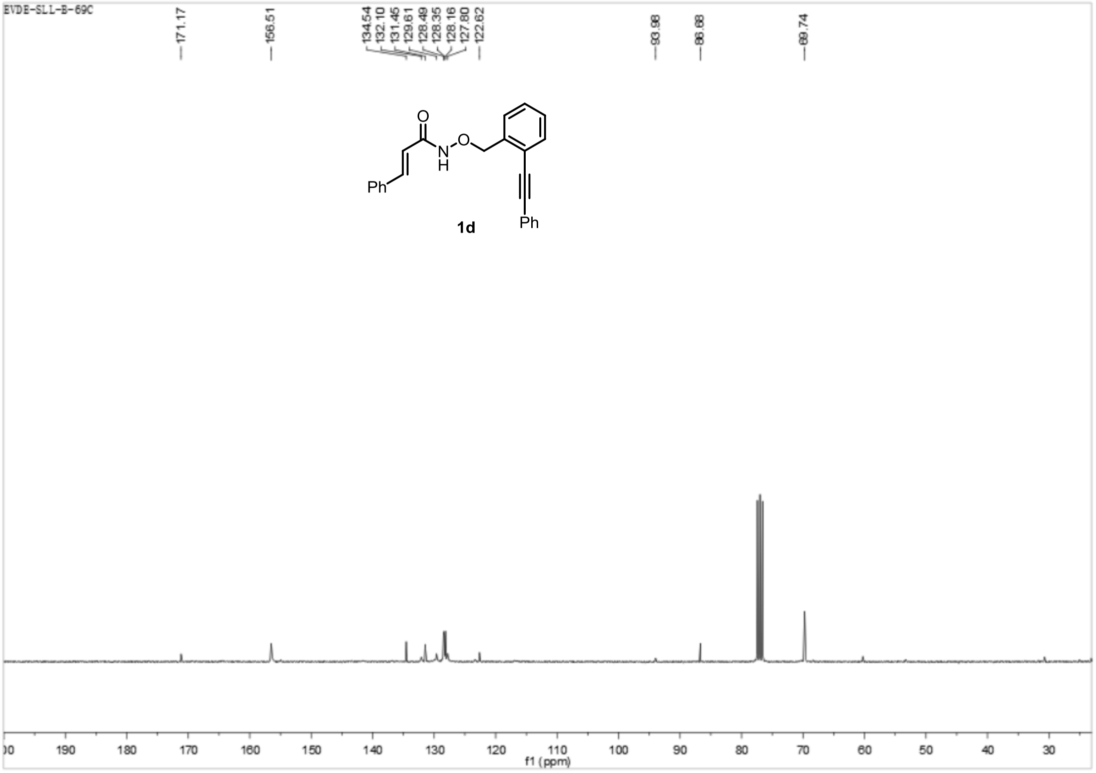
EVDE-SLL-B-69C
171.17
156.51
134.54
132.10
131.45
129.61
128.49
128.35
128.16
127.80
122.62
93.98
86.88
69.74
Ph
1d
f1 (ppm)

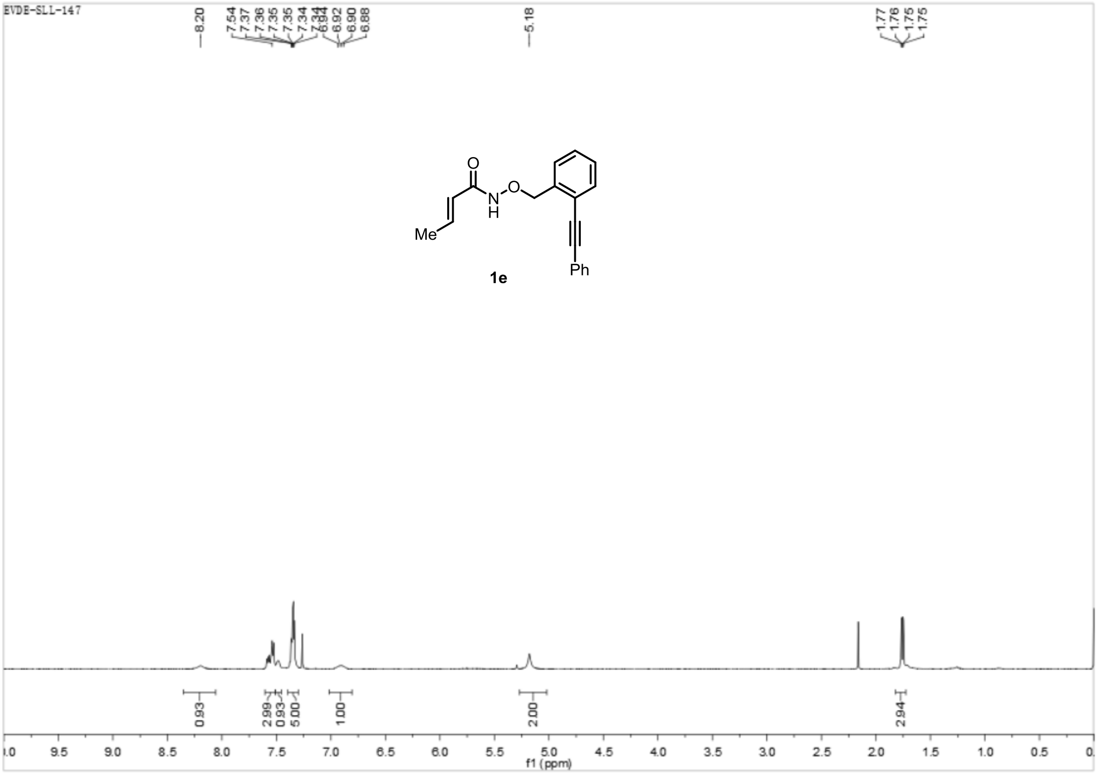
EVDE-SLL-147
8.20
7.54
7.37
7.36
7.35
7.35
7.34
7.34
6.94
6.92
6.90
6.88
5.18
1.77
1.76
1.75
1.75
O
N
H
O
Me
Ph
1e
0.93
2.99
0.93
5.00
1.00
2.00
2.94
f1 (ppm)

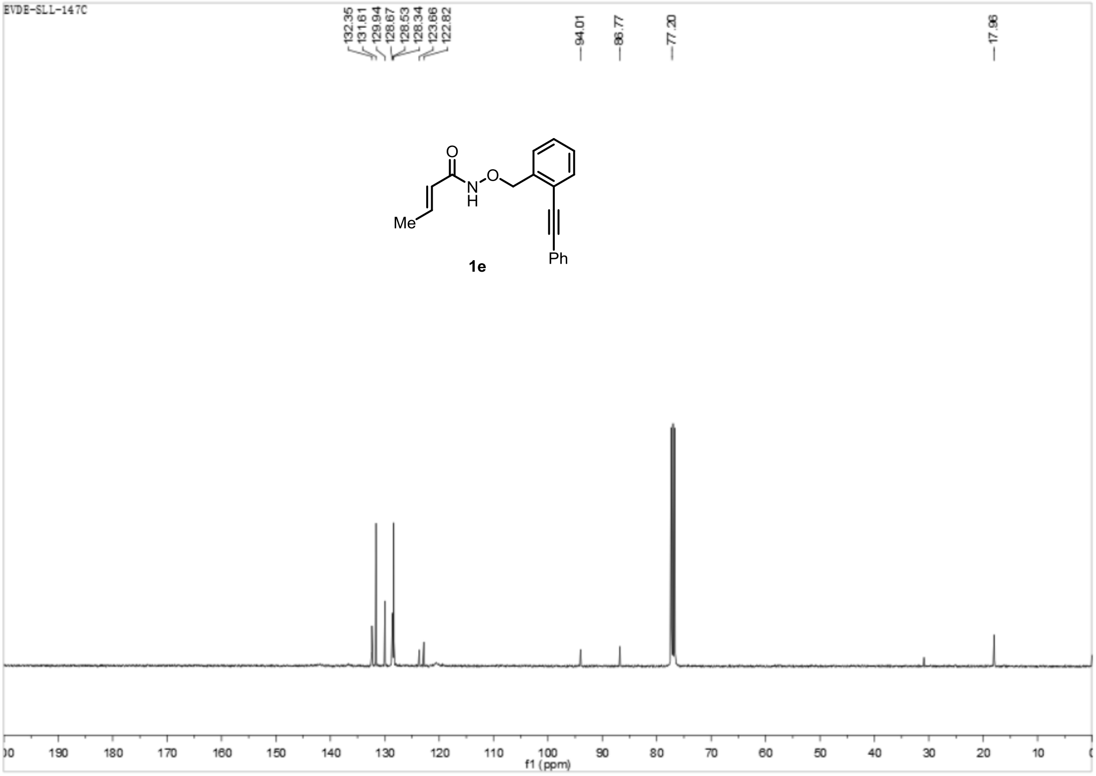
EVDE-SLL-147C
132.35
131.61
129.94
128.67
128.53
128.34
123.66
122.82
94.01
86.77
77.20
17.96
O
N
H
O
Me
Ph
1e
f1 (ppm)

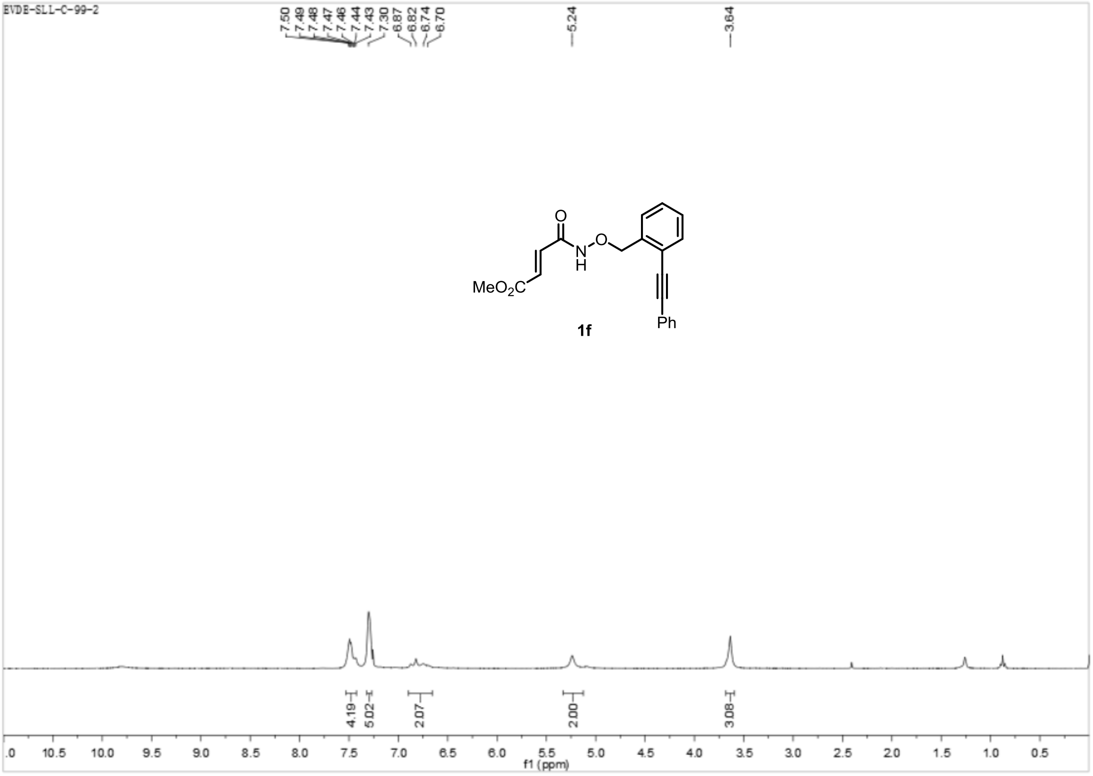
EVDE-SLL-C-99-2
7.50 7.49 7.48 7.47 7.46 7.44 7.43 7.30 6.87 6.82 6.74 6.70
5.24
3.64
1f
4.19 5.02 2.07 2.00 3.08
f1 (ppm)

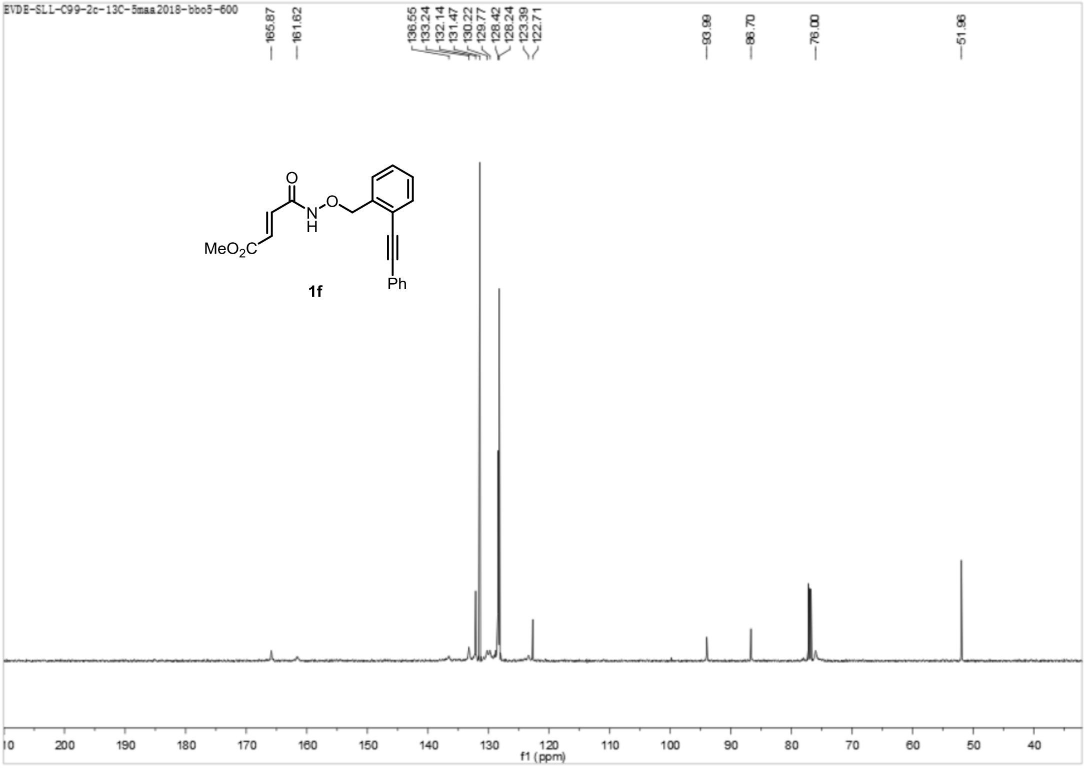
EVDE-SLL-C99-2c-13C-5maa2018-bbo5-600
165.87
161.62
136.55 133.24 132.14 131.47 130.22 129.77 128.42 128.24 123.39 122.71
93.99
86.70
76.00
51.96
1f
f1 (ppm)

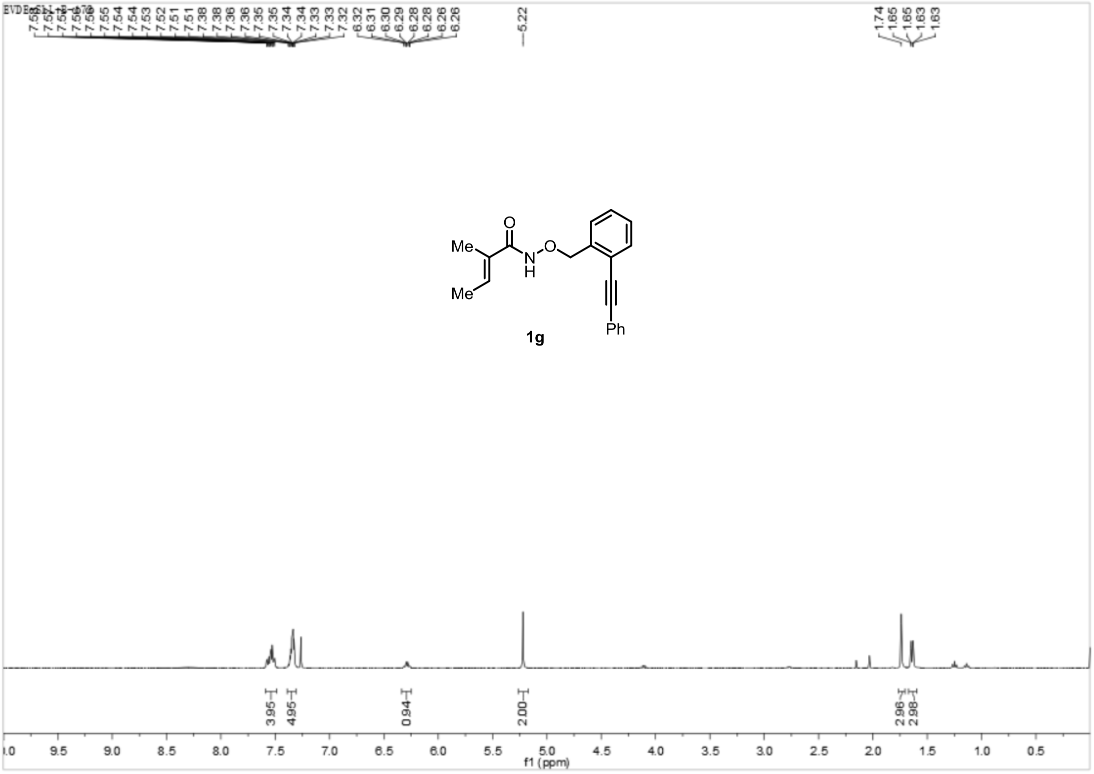

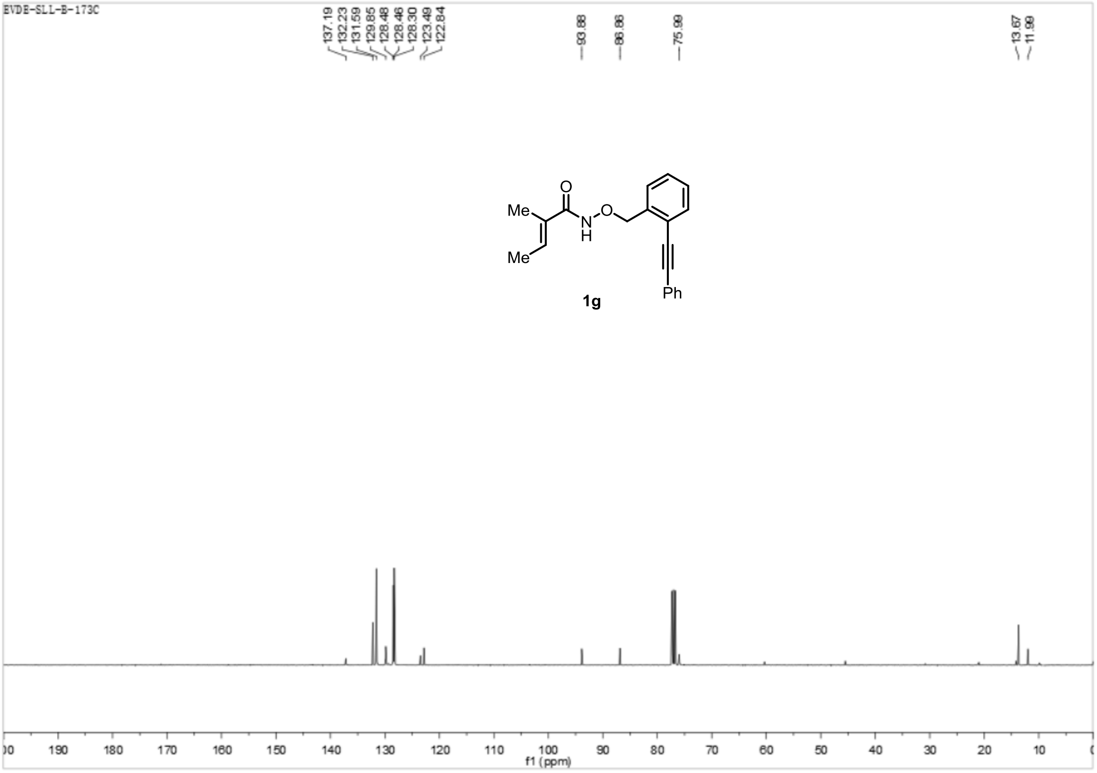

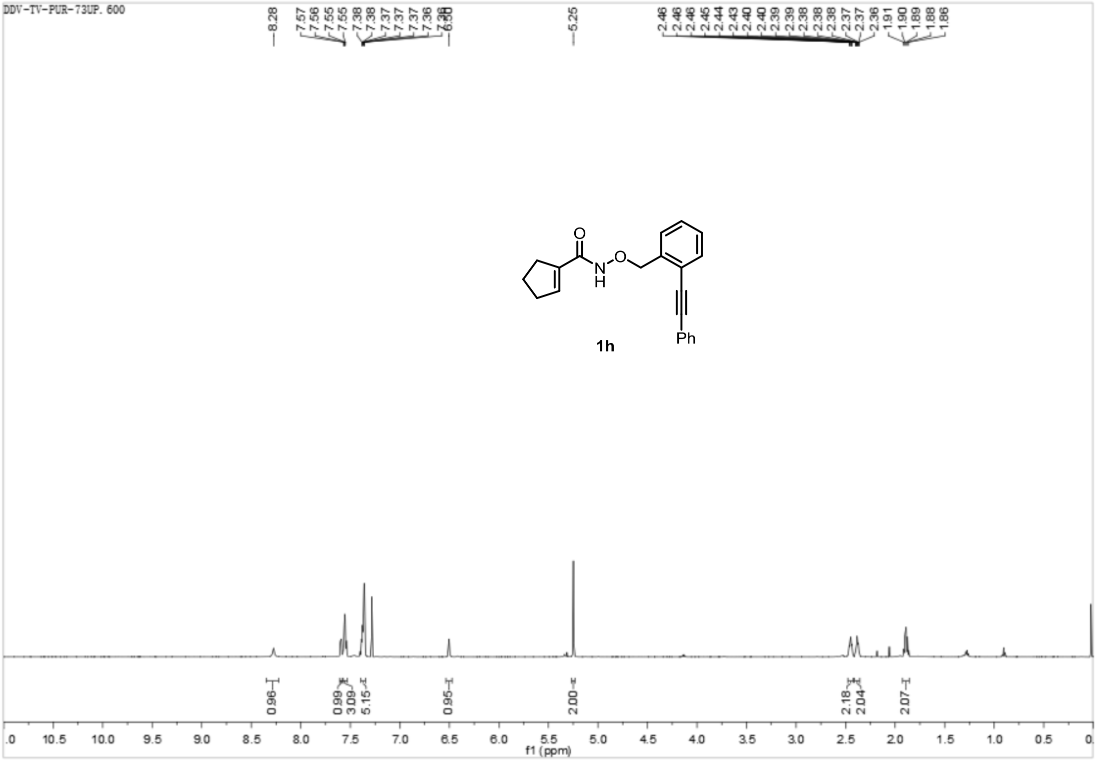
DDV-TV-PUR-73UP.600
1h
Ph
f1 (ppm)

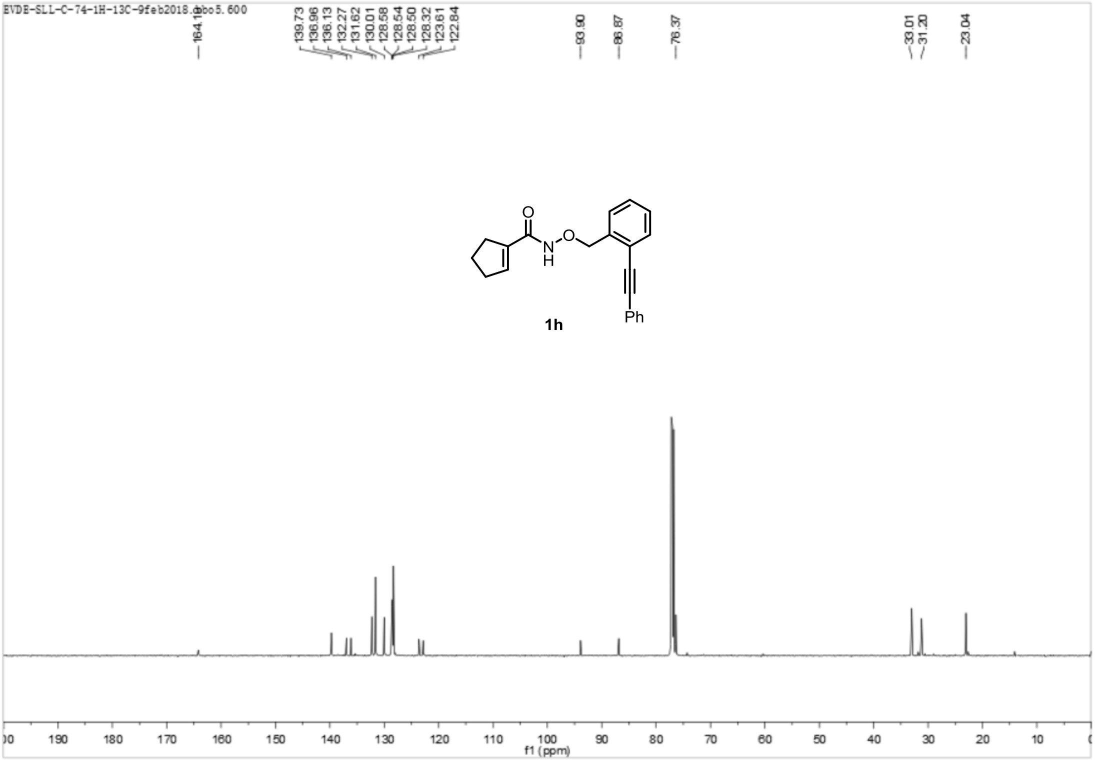
EVDE-SLL-C-74-1H-13C-9feb2018.bbo5.600
1h
Ph
f1 (ppm)

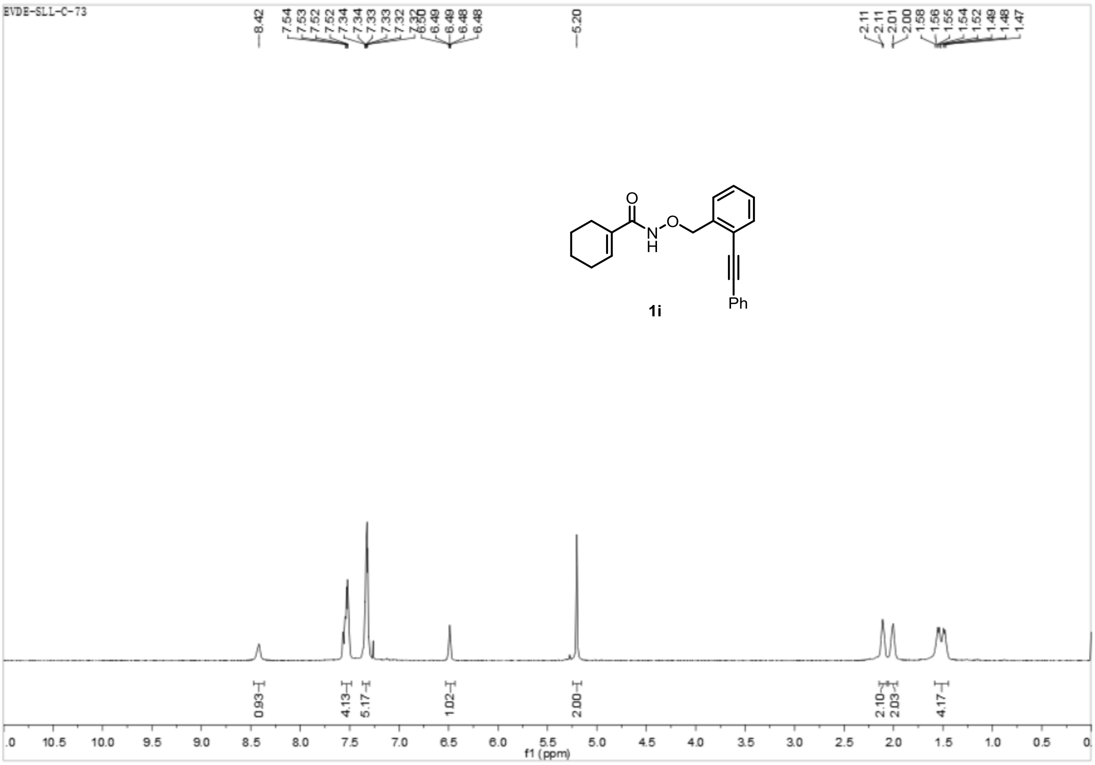
EVDE-SLL-C-73
8.42
7.54
7.53
7.52
7.52
7.34
7.33
7.33
7.32
7.32
6.50
6.49
6.49
6.48
6.48
5.20
2.11
2.11
2.01
2.00
1.58
1.56
1.55
1.54
1.52
1.49
1.48
1.47
O
N
H
O
Ph
1i
0.93
4.13
5.17
1.02
2.00
2.10
2.03
4.17
f1 (ppm)

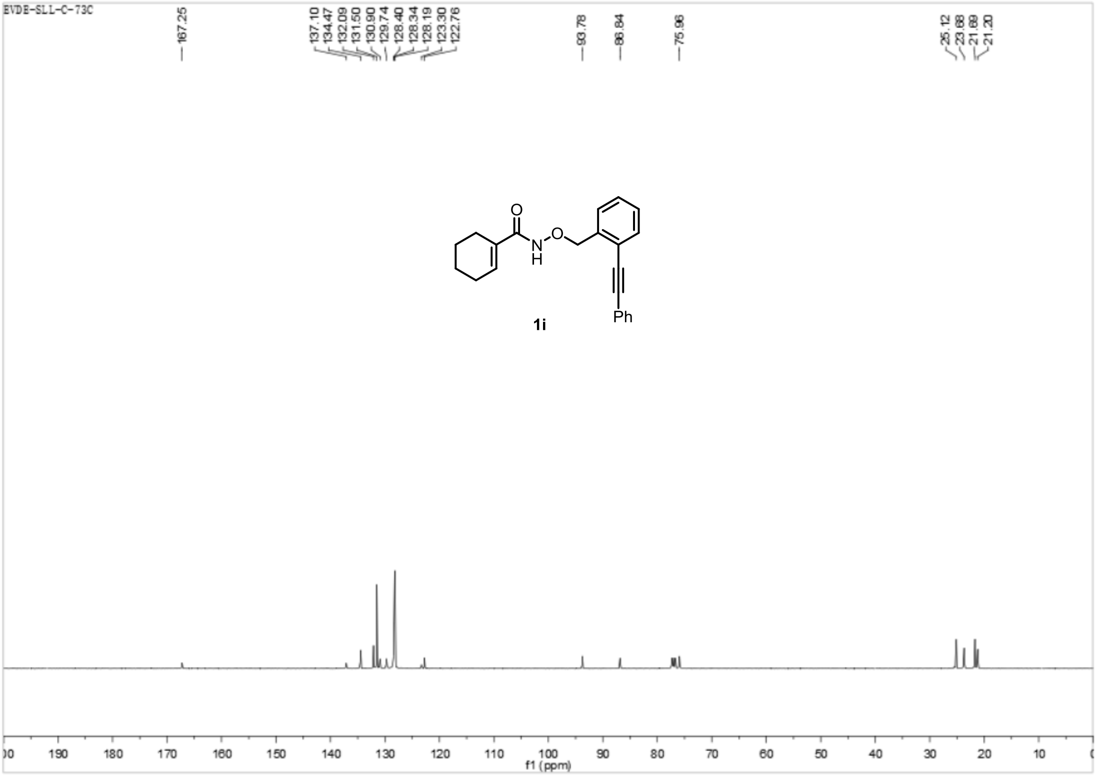
EVDE-SLL-C-73C
167.25
137.10
134.47
132.09
131.50
130.90
129.74
128.40
128.34
128.19
123.30
122.76
93.78
86.84
75.96
25.12
23.88
21.89
21.20
O
N
H
O
Ph
1i
f1 (ppm)

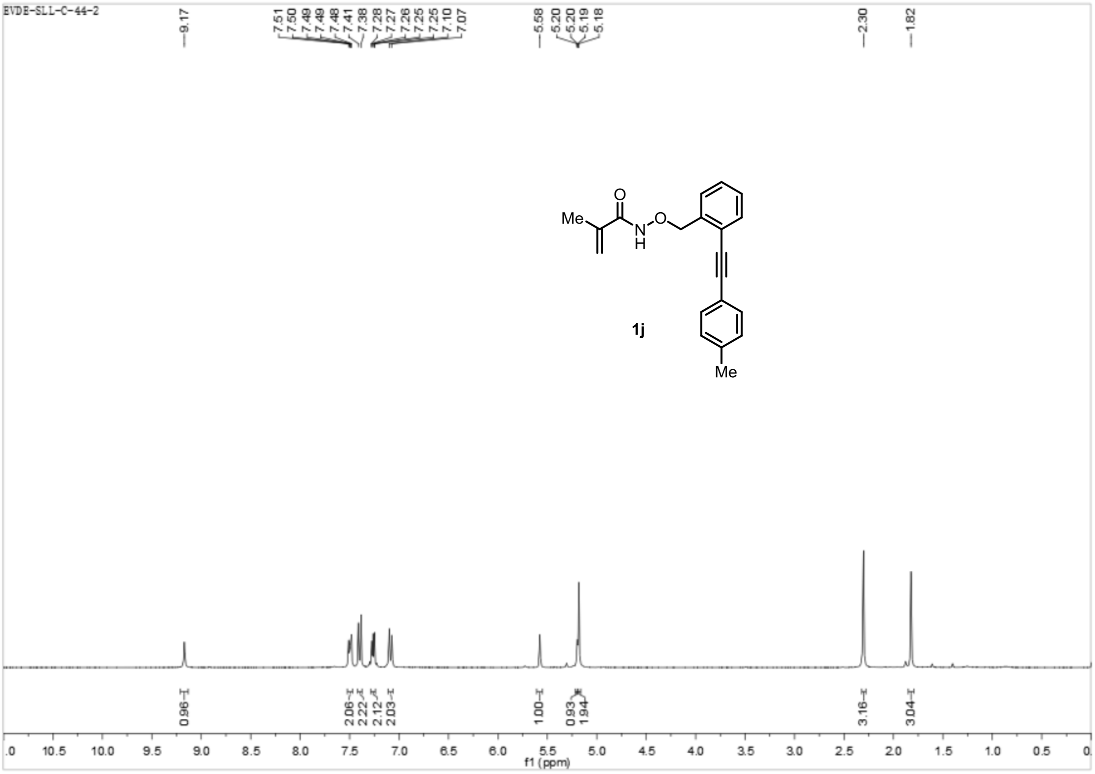
EVDE-SLL-C-44-2
9.17
7.51 7.50 7.49 7.49 7.48 7.41 7.38 7.28 7.27 7.26 7.25 7.25 7.10 7.07
5.58 5.20 5.20 5.19 5.18
2.30
1.82
Me
O
N
H
O
1j
Me
0.96
2.06 2.22 2.12 2.03
1.00
0.93 1.94
3.16
3.04
f1 (ppm)

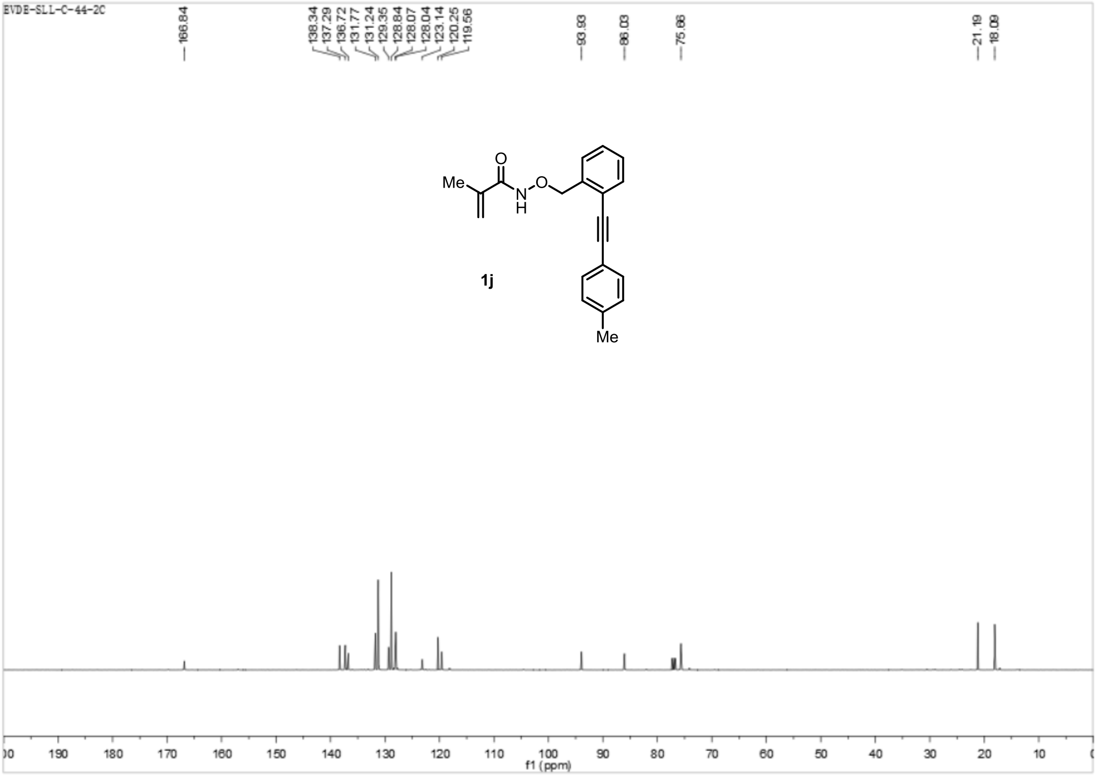
EVDE-SLL-C-44-2C
166.84
138.34 137.29 136.72 131.77 131.24 129.35 128.84 128.07 128.04 123.14 120.25 119.56
93.93
86.03
75.66
21.19 18.09
Me
O
N
H
O
1j
Me
f1 (ppm)

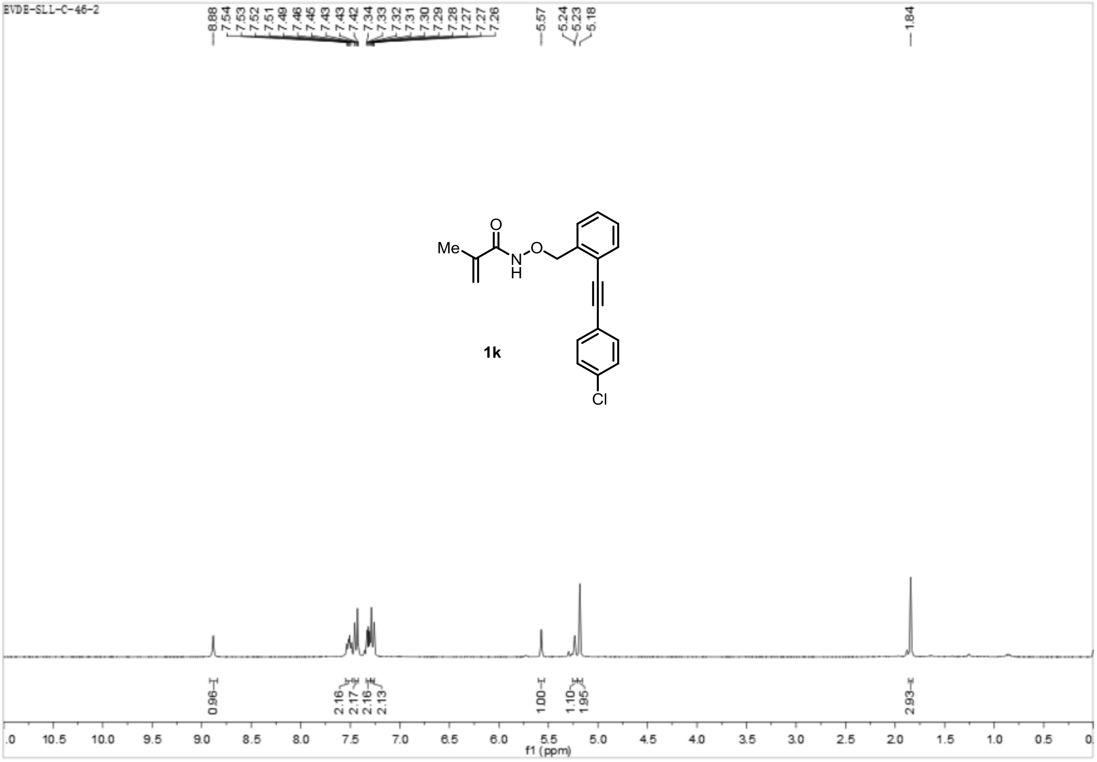
EVDE-SLL-C-46-2
8.88
7.54
7.53
7.52
7.51
7.49
7.46
7.45
7.43
7.43
7.42
7.34
7.33
7.32
7.31
7.30
7.29
7.28
7.27
7.26
5.57
5.24
5.23
5.18
1.84
Me
O
N
H
O
Cl
1k
0.96
2.16
2.17
2.16
2.13
1.00
1.10
1.95
2.93
f1 (ppm)

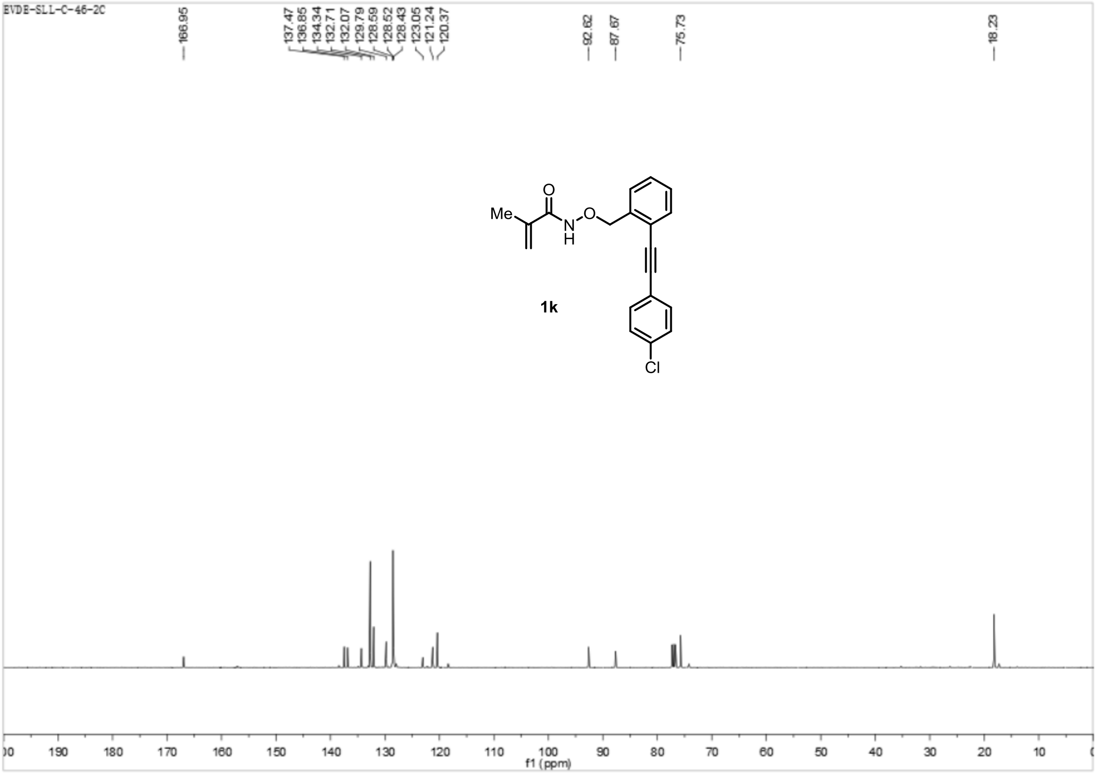
EVDE-SLL-C-46-2C
166.95
137.47
136.85
134.34
132.71
132.07
129.79
128.59
128.52
128.43
123.05
121.24
120.37
92.62
87.67
75.73
18.23
Me
O
N
H
O
Cl
1k
f1 (ppm)

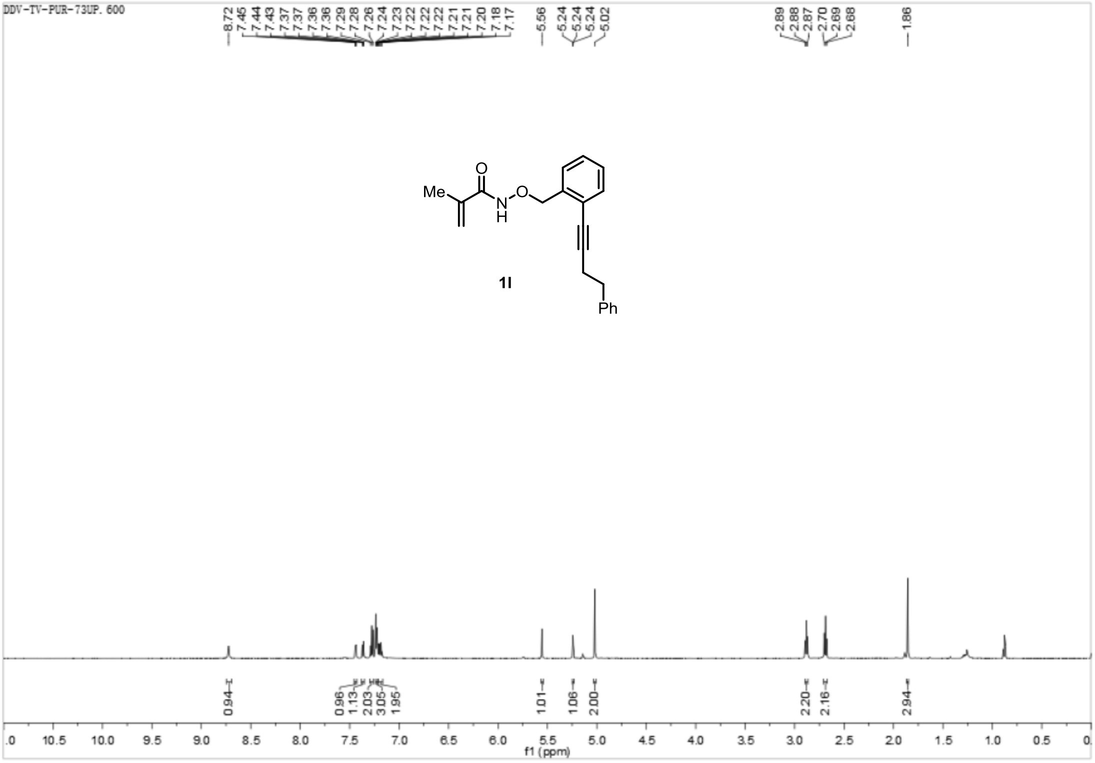
DDV-TV-PUR-73UP.600
Me
O
N
H
O
1l
Ph
f1 (ppm)

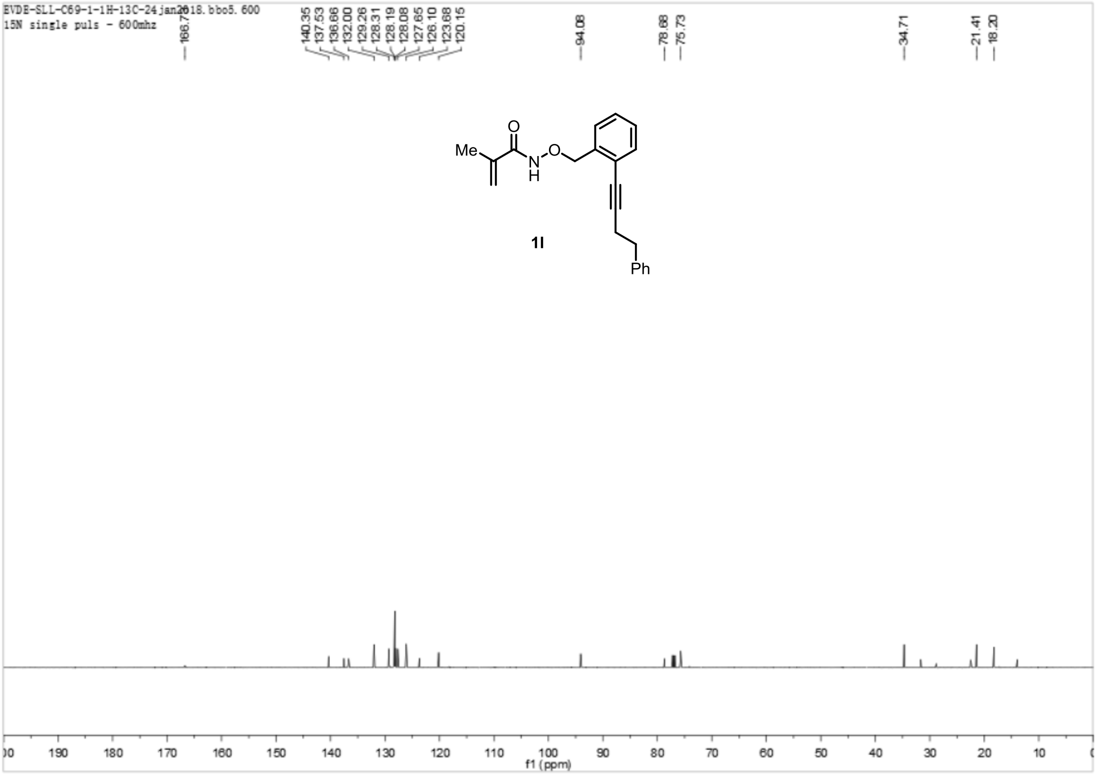
EVDE-SLL-C69-1-1H-13C-24jan2018.bbo5.600
15N single puls - 600mhz
Me
O
N
H
O
1l
Ph
f1 (ppm)

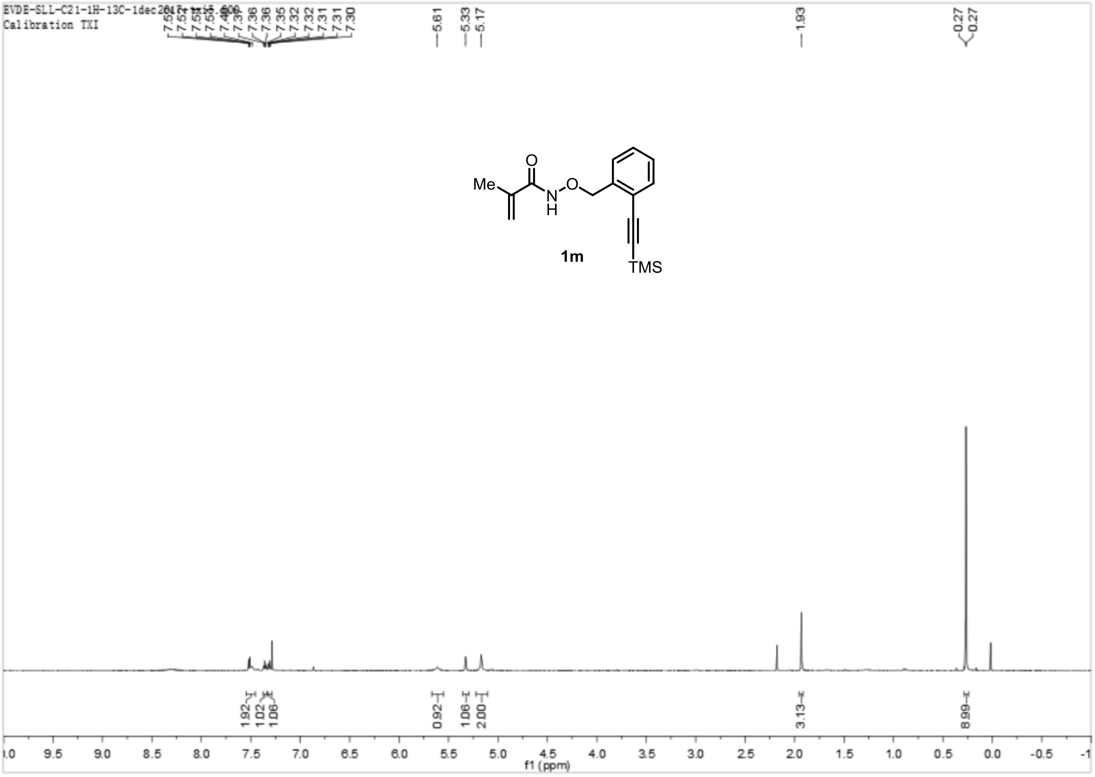
EVDE-SLL-C21-1H-13C-1dec2017-txi5.600
Calibration TXI
5.61
5.33
5.17
1.93
0.27
0.27
Me
O
N
H
O
1m
TMS
1.92
1.02
1.06
0.92
1.06
2.00
3.13
8.99
f1 (ppm)

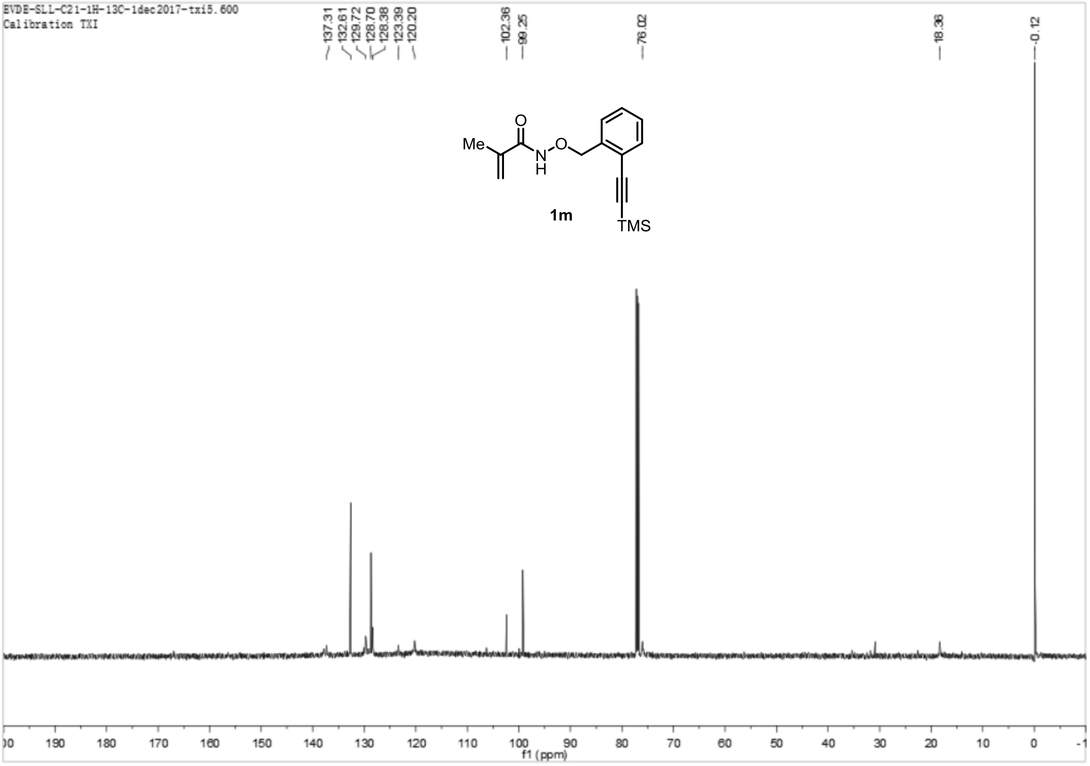
EVDE-SLL-C21-1H-13C-1dec2017-txi5.600
Calibration TXI
137.31
132.61
129.72
128.70
128.38
123.39
120.20
102.36
99.25
76.02
18.36
-0.12
Me
O
N
H
O
1m
TMS
f1 (ppm)

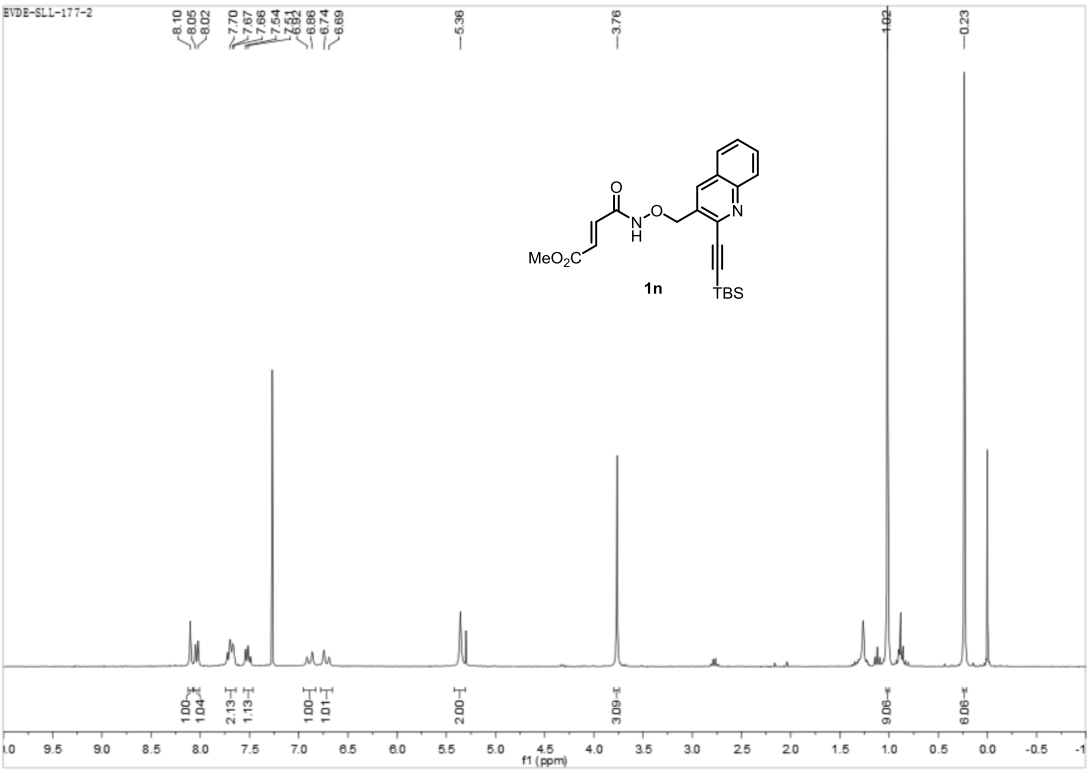
EVDE-SLL-177-2
MeO2C
1n
TBS
f1 (ppm)

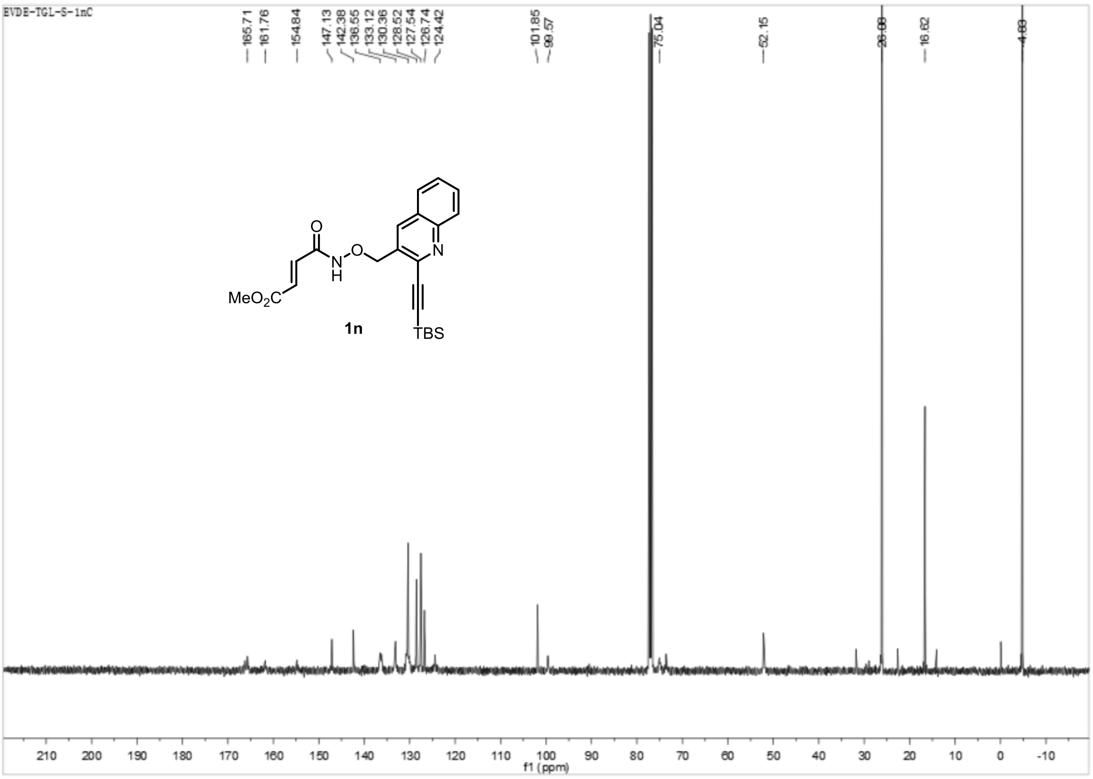
EVDE-TGL-S-1nC
MeO2C
1n
TBS
f1 (ppm)

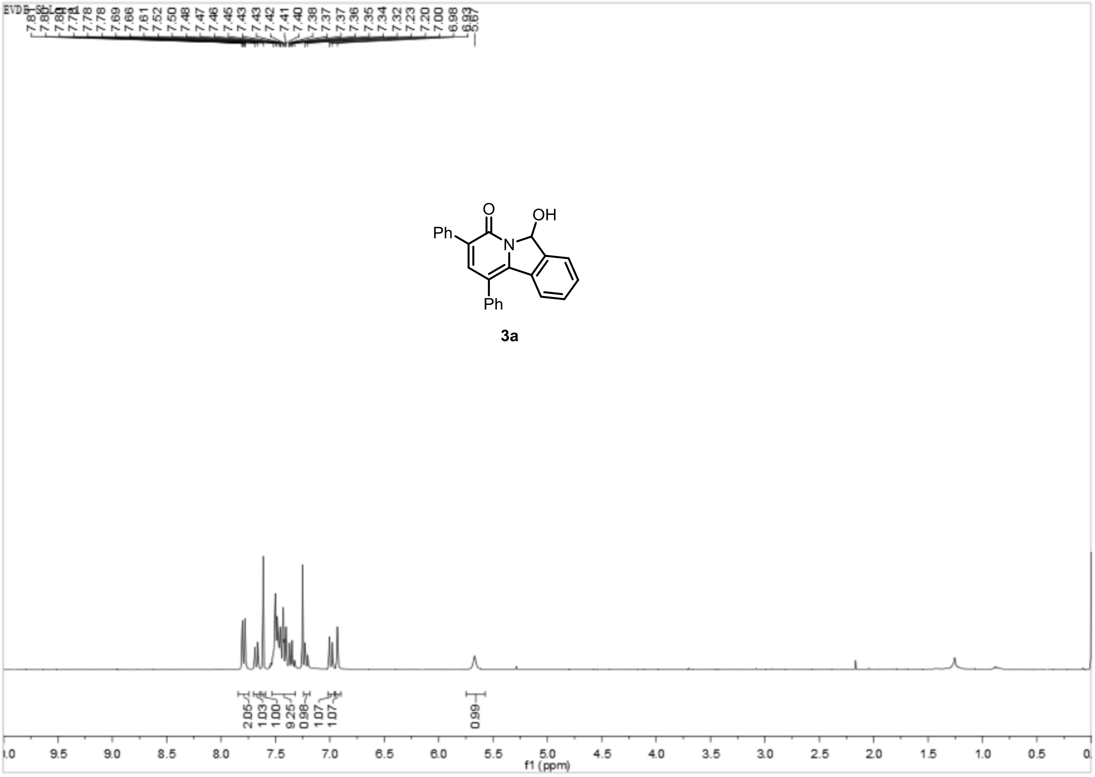

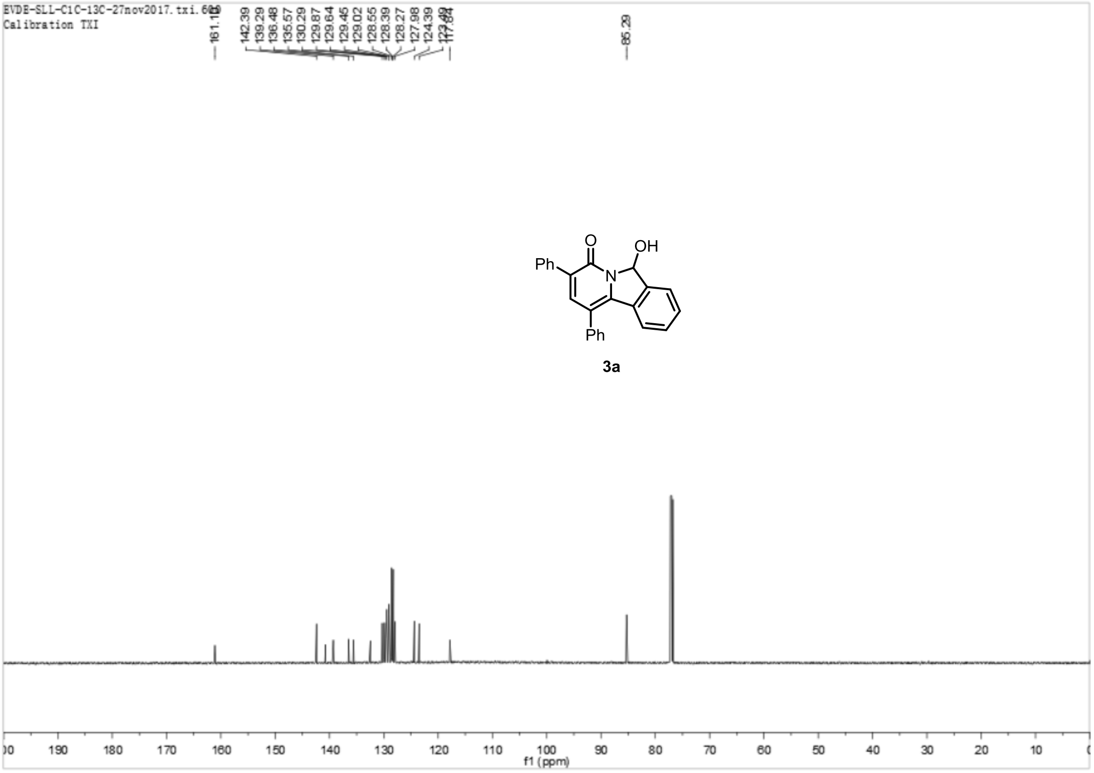

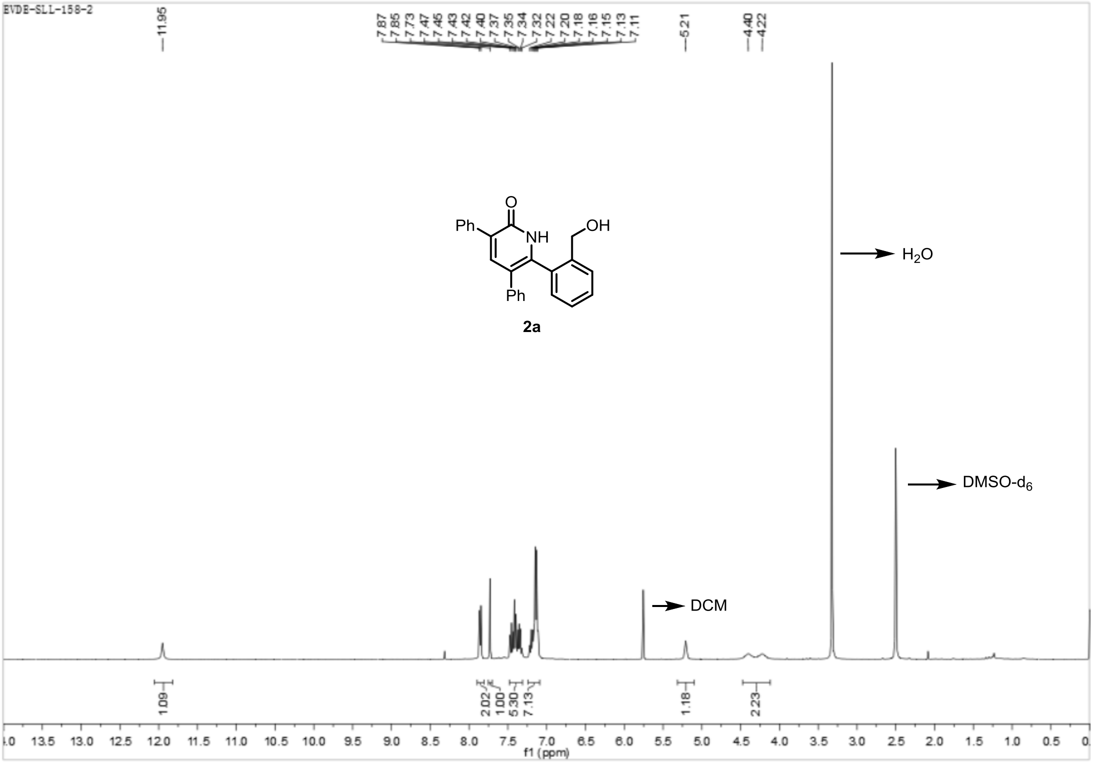
EVDE-SLL-158-2
11.95
7.87
7.85
7.73
7.47
7.45
7.43
7.42
7.40
7.37
7.35
7.34
7.32
7.22
7.20
7.18
7.16
7.15
7.13
7.11
5.21
4.40
4.22
Ph
O
NH
OH
Ph
2a
H2O
DMSO-d6
DCM
1.09
2.02
1.00
5.30
7.13
1.18
2.23
f1 (ppm)

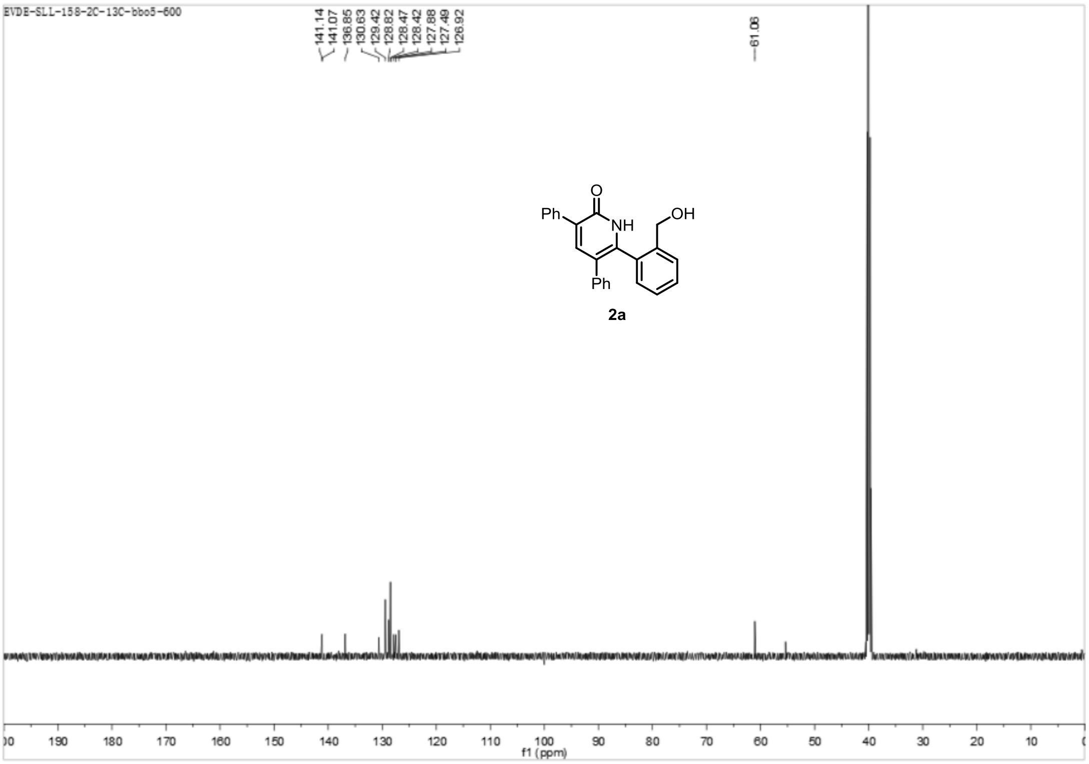
EVDE-SLL-158-2C-13C-bbo5-600
141.14
141.07
136.85
130.63
129.42
128.82
128.47
128.42
127.88
127.49
126.92
61.06
Ph
O
NH
OH
Ph
2a
f1 (ppm)

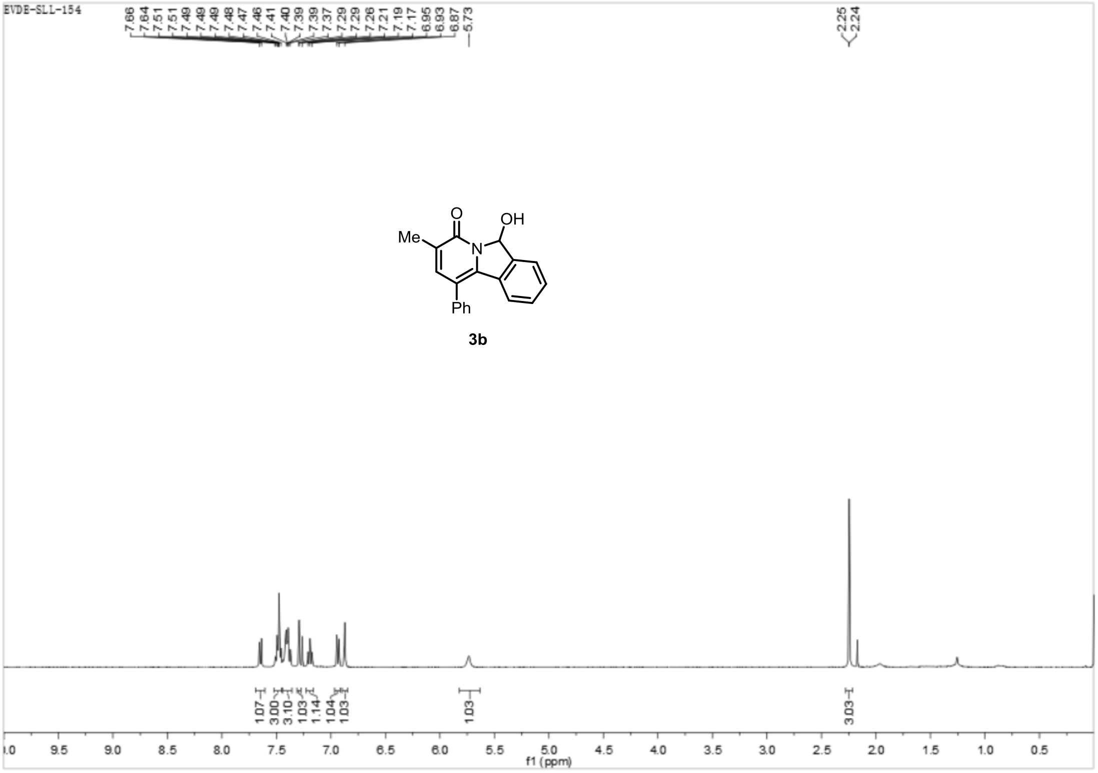
EVDE-SLL-154
7.66 7.64 7.51 7.49 7.49 7.49 7.48 7.47 7.46 7.41 7.40 7.39 7.39 7.37 7.29 7.29 7.26 7.21 7.19 7.17 6.95 6.93 6.87 5.73
2.25 2.24
Me
O
N
OH
Ph
3b
1.07 3.00 3.10 1.03 1.14 1.04 1.03
1.03
3.03
f1 (ppm)

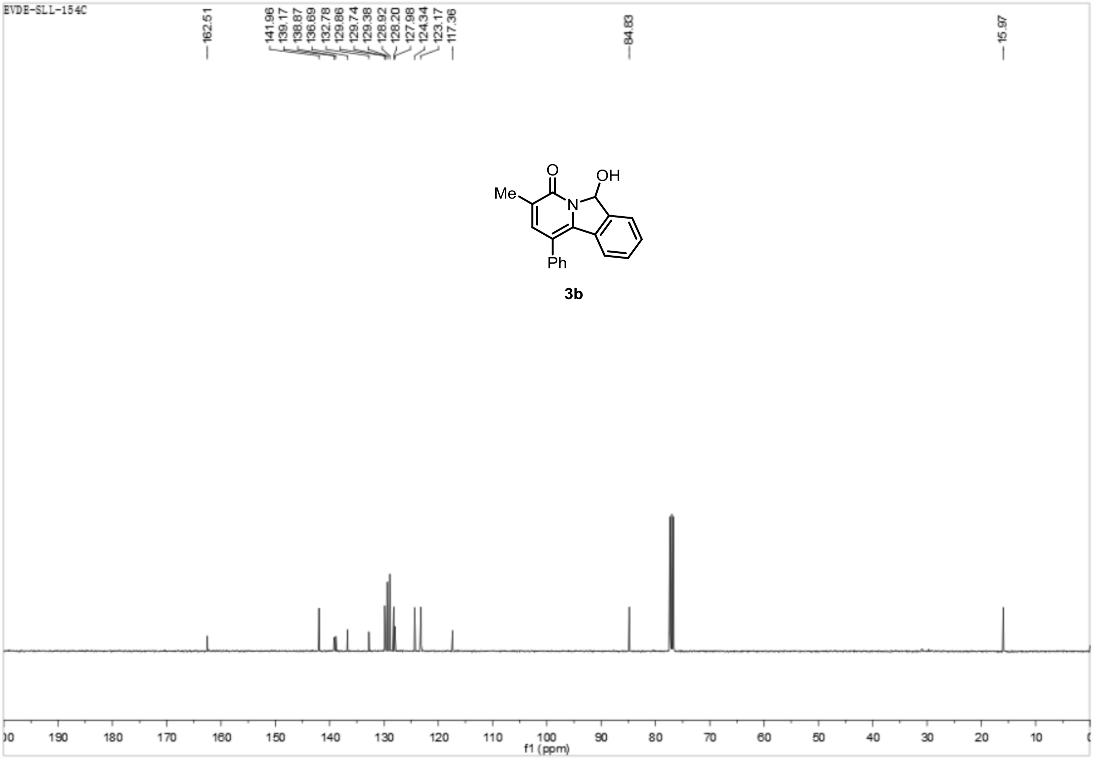
EVDE-SLL-154C
162.51
141.96 139.17 138.87 136.69 132.78 129.86 129.74 129.38 128.92 128.20 127.98 124.34 123.17 117.36
84.83
15.97
Me
O
N
OH
Ph
3b
f1 (ppm)

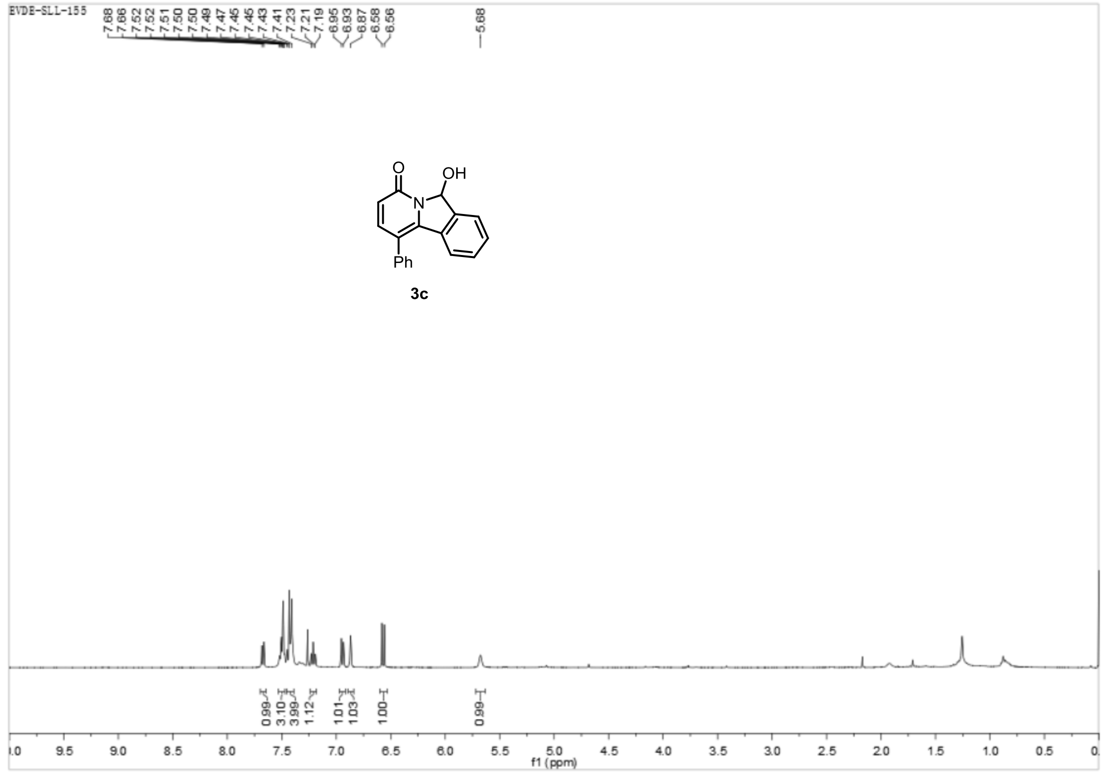

EVDE-SLL-155
7.68
7.66
7.52
7.52
7.51
7.50
7.50
7.49
7.47
7.45
7.45
7.43
7.41
7.23
7.21
7.19
6.95
6.93
6.87
6.58
6.56
5.68
O
OH
N
Ph
3c
0.99
3.10
3.99
1.12
1.01
1.03
1.00
0.99
f1 (ppm)

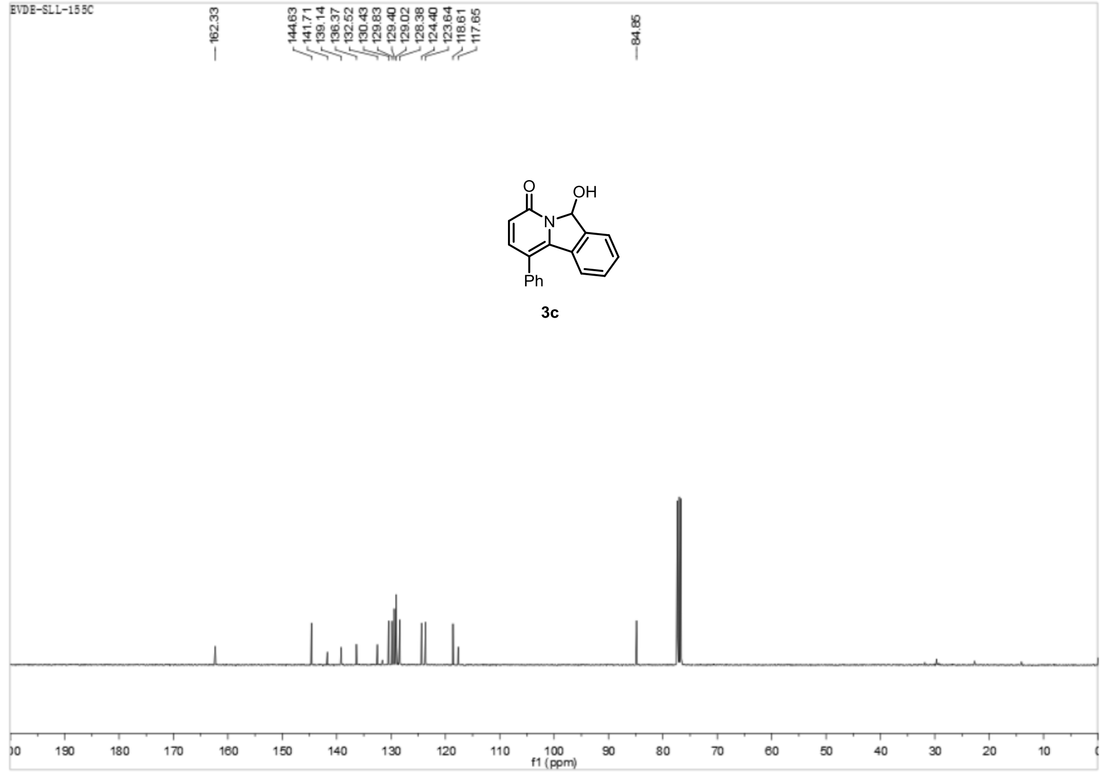

EVDE-SLL-155C
162.33
144.63
141.71
139.14
136.37
132.52
130.43
129.83
129.40
129.02
128.38
124.40
123.64
118.61
117.65
84.85
O
OH
N
Ph
3c
f1 (ppm)

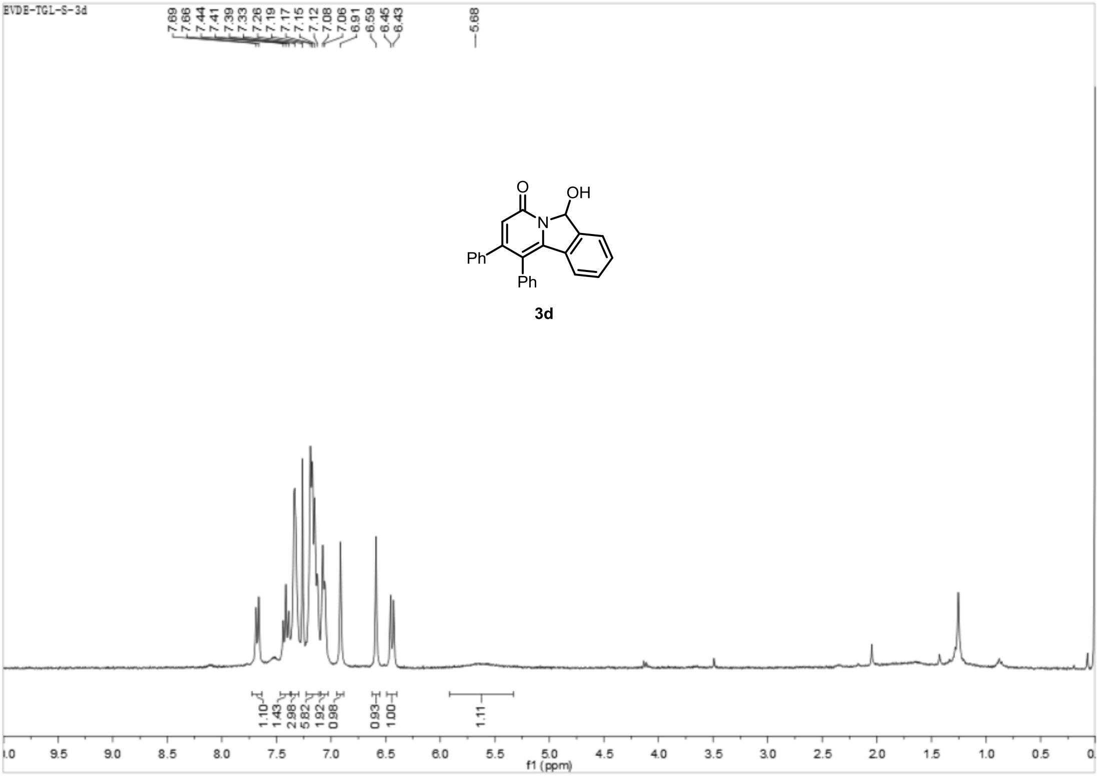
EVDE-TGL-S-3d
OH
Ph
Ph
3d
f1 (ppm)

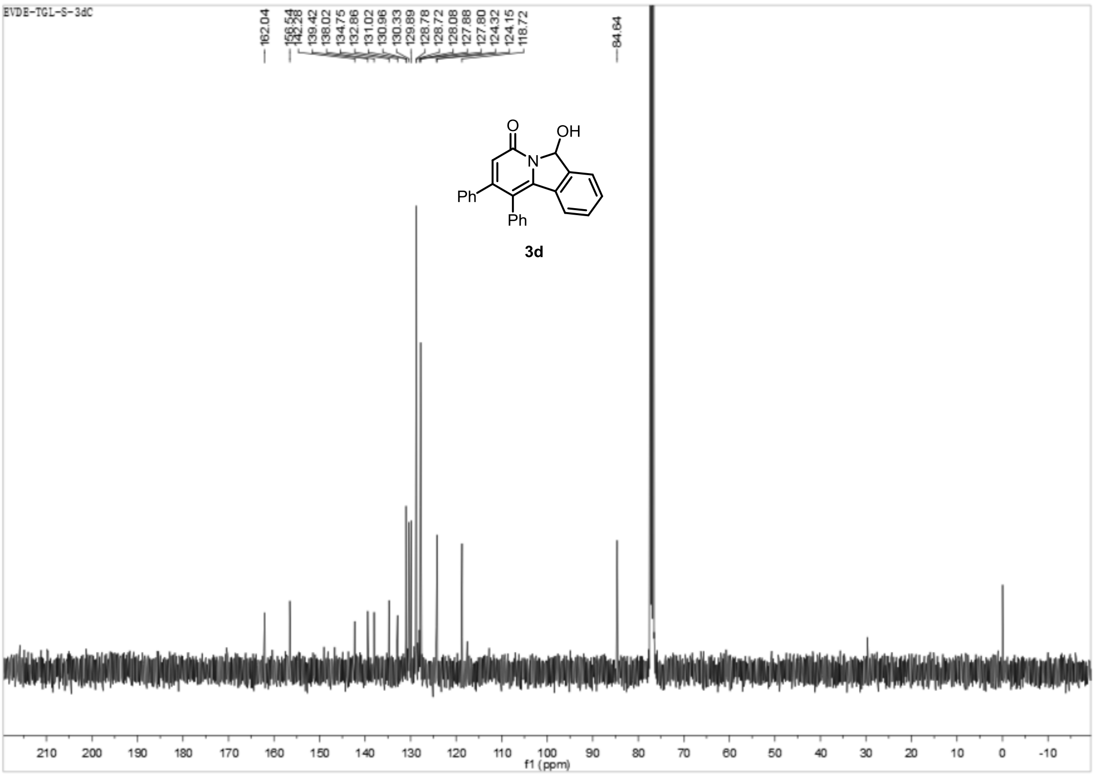
EVDE-TGL-S-3dC
OH
Ph
Ph
3d
f1 (ppm)

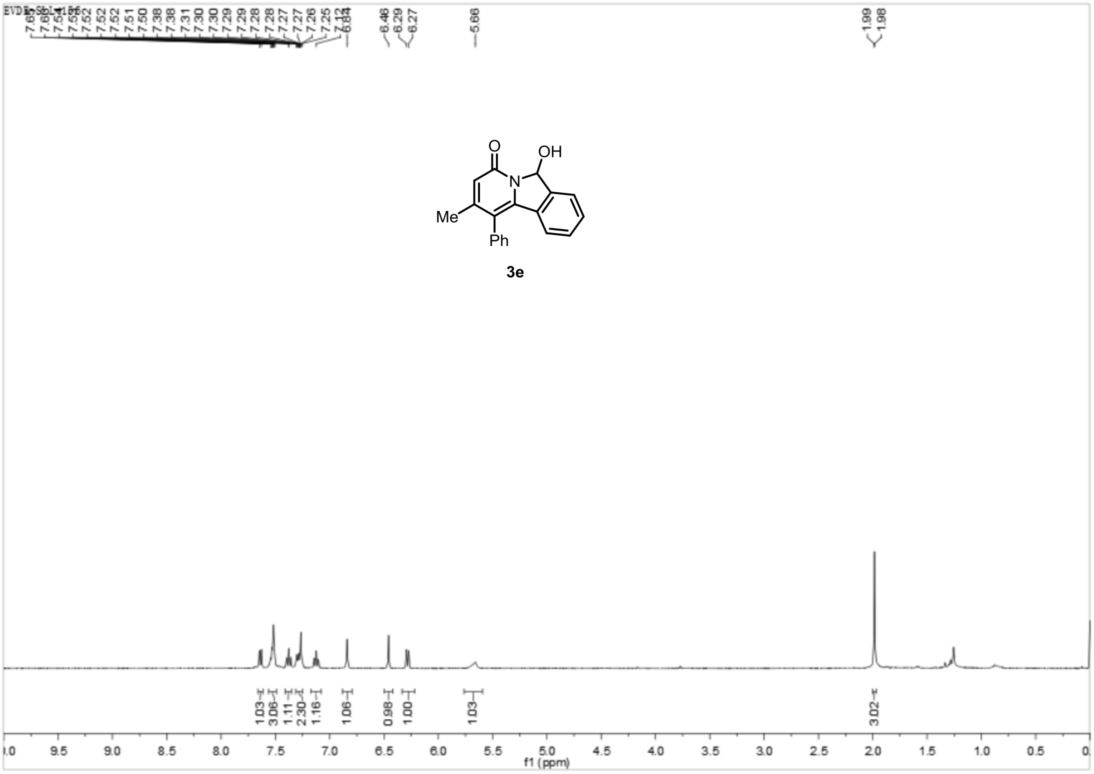

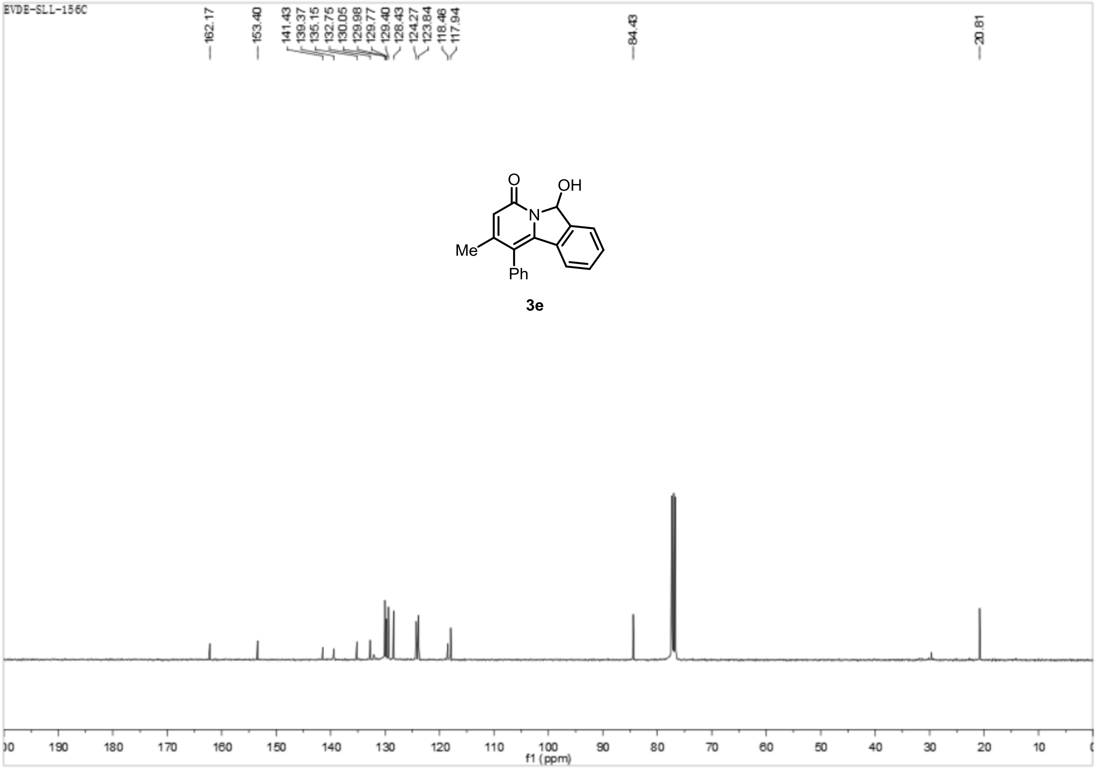

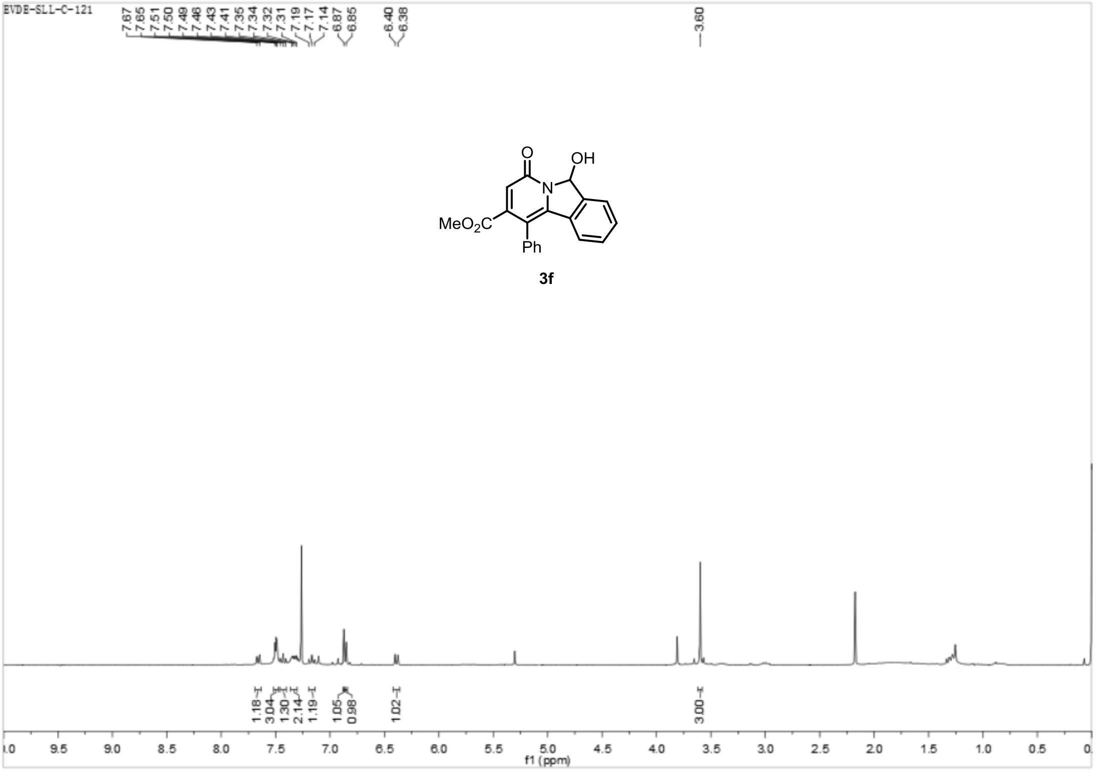
EVDE-SLL-C-121
7.67 7.65 7.51 7.50 7.49 7.46 7.43 7.41 7.35 7.34 7.32 7.31 7.19 7.17 7.14 6.87 6.85 6.40 6.38
3.60
MeO2C
Ph
OH
N
O
3f
1.18 3.04 1.30 2.14 1.19 1.05 0.98 1.02
3.00
f1 (ppm)

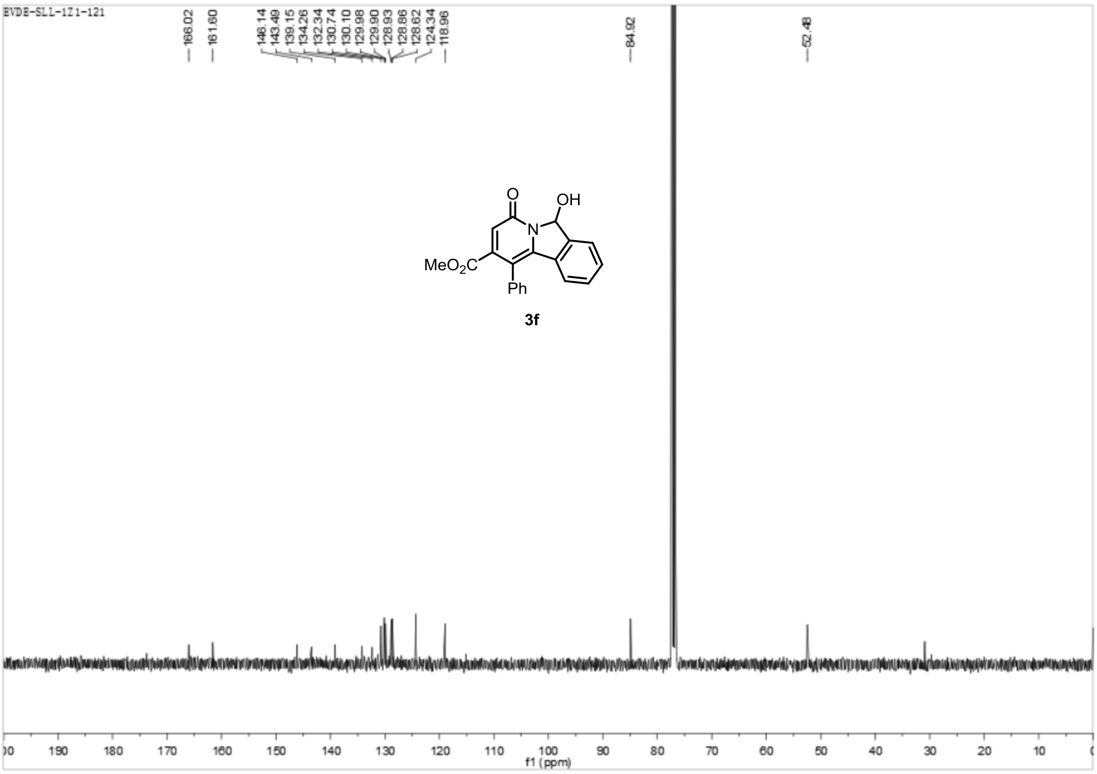
EVDE-SLL-1Z1-121
166.02 161.60
146.14 143.49 139.15 134.26 132.34 130.74 130.10 129.98 129.90 128.93 128.86 128.62 124.34 118.96
84.92
52.48
MeO2C
Ph
OH
N
O
3f
f1 (ppm)

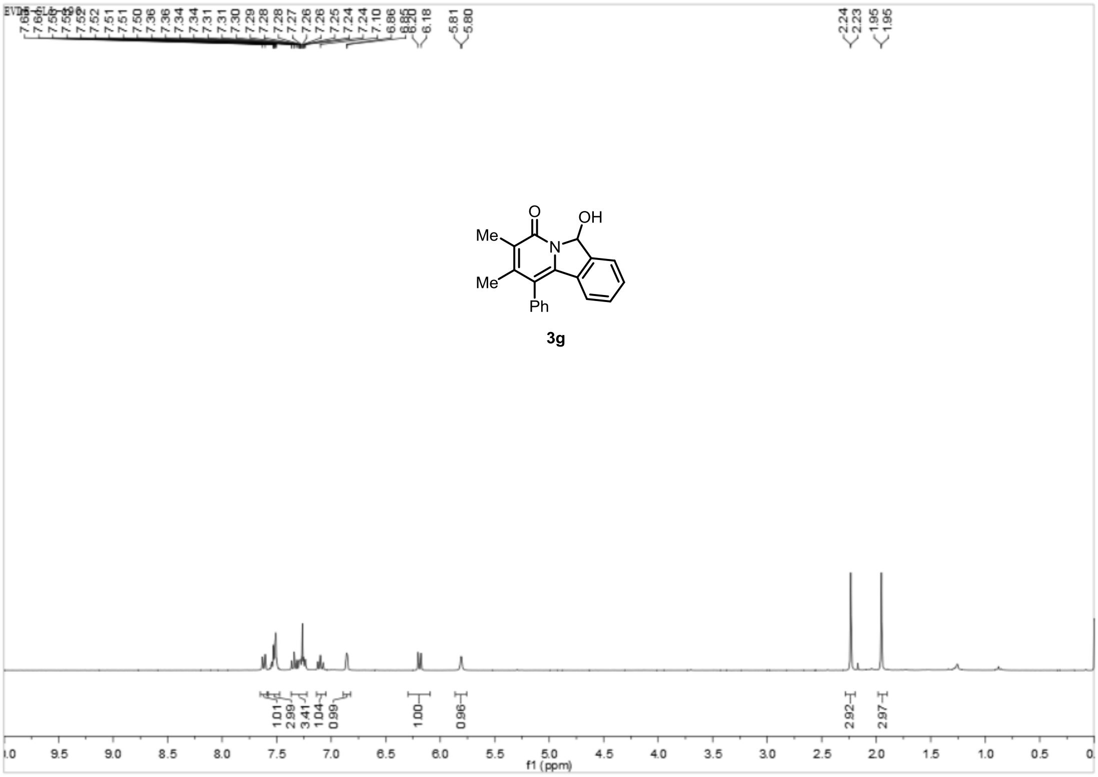
7.65
7.61
7.52
7.51
7.50
7.36
7.34
7.31
7.30
7.29
7.28
7.27
7.26
7.25
7.24
7.10
6.86
6.18
5.81
5.80
2.24
2.23
1.95
1.95
Me
Me
Ph
OH
N
O
3g
1.01
2.99
3.41
1.04
0.99
1.00
0.96
2.92
2.97
f1 (ppm)

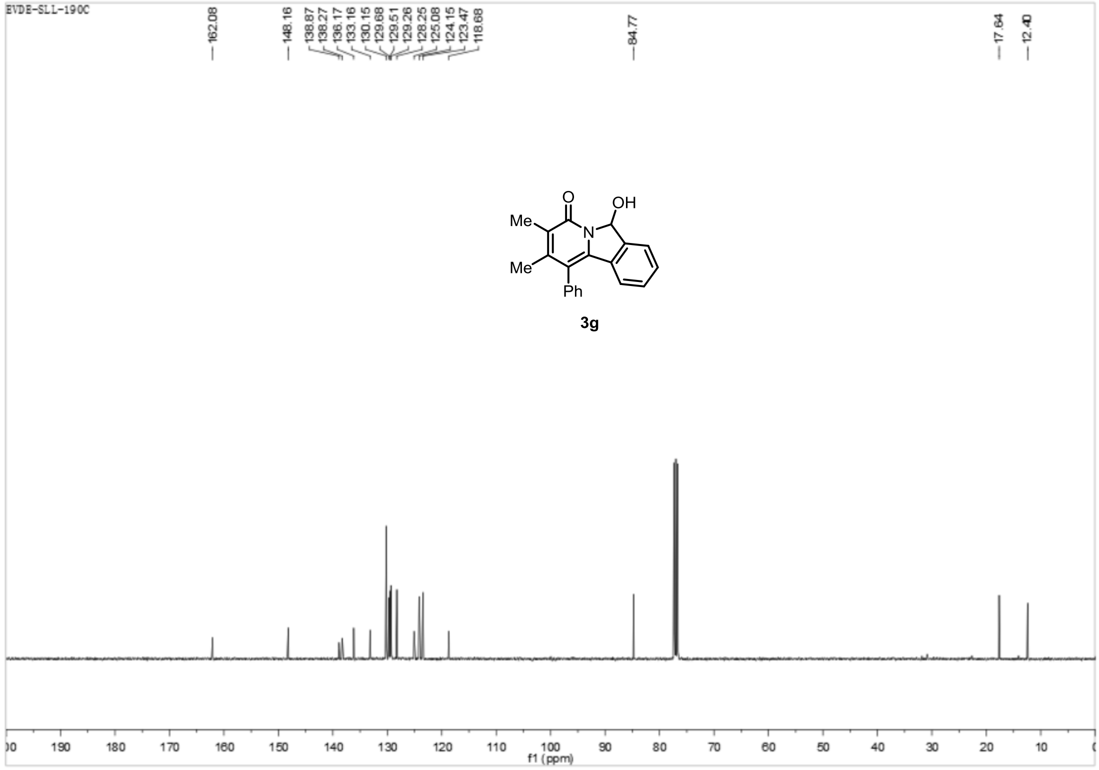
EVDE-SLL-190C
162.08
148.16
138.87
138.27
136.17
133.16
130.15
129.68
129.51
129.26
128.25
125.08
124.15
123.47
118.68
84.77
17.64
12.40
Me
Me
Ph
OH
N
O
3g
f1 (ppm)

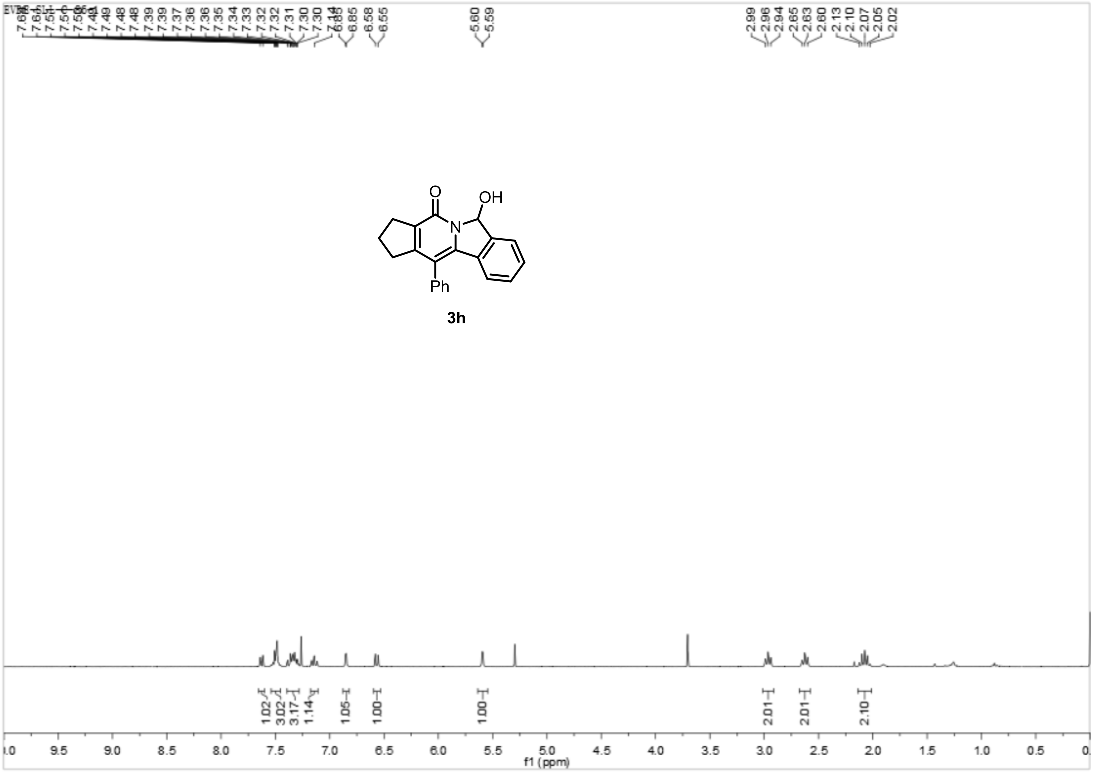
7.49 7.48 7.48 7.39 7.39 7.37 7.36 7.35 7.34 7.33 7.32 7.32 7.31 7.30 7.30 7.14 6.85 6.85 6.58 6.55
5.60 5.59
2.99 2.96 2.94 2.65 2.63 2.60 2.13 2.10 2.07 2.05 2.02
OH
O
N
Ph
3h
1.02 3.02 3.17 1.14 1.05 1.00 1.00 2.01 2.01 2.10
f1 (ppm)

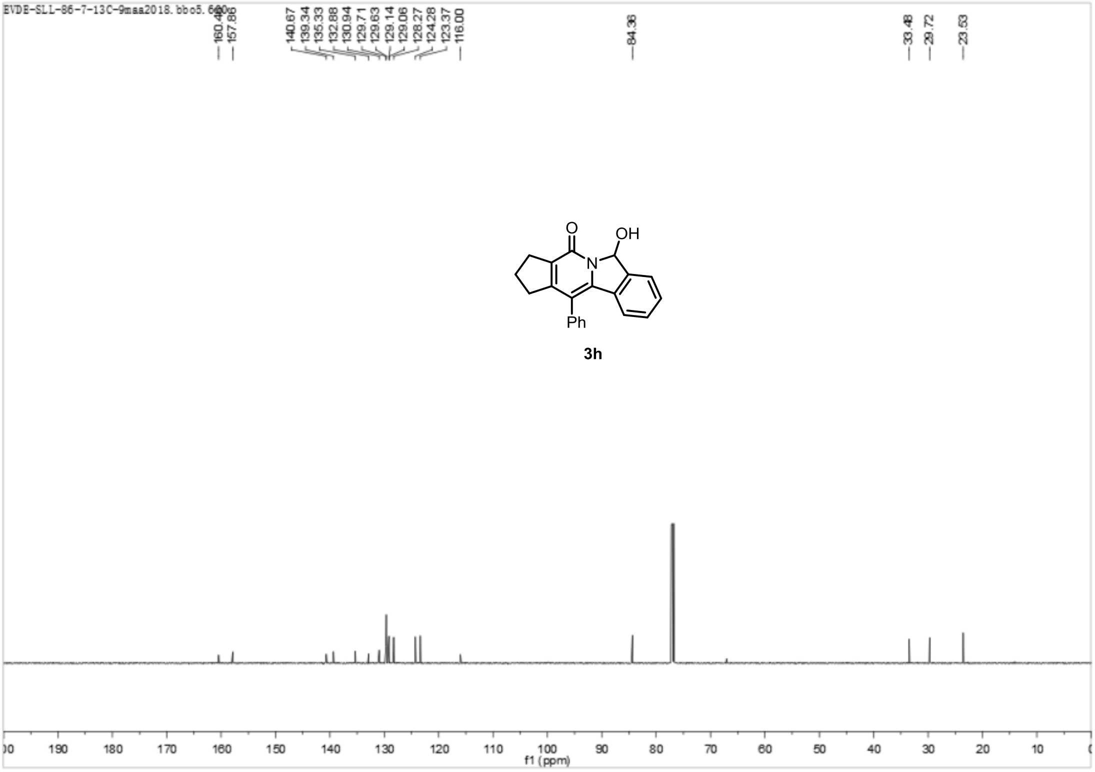
EVDE-SLL-86-7-13C-9maa2018.bbo5.600
157.86
140.67 139.34 135.33 132.88 130.94 129.71 129.63 129.14 129.06 128.27 124.28 123.37 116.00
84.36
33.48 29.72 23.53
OH
O
N
Ph
3h
f1 (ppm)

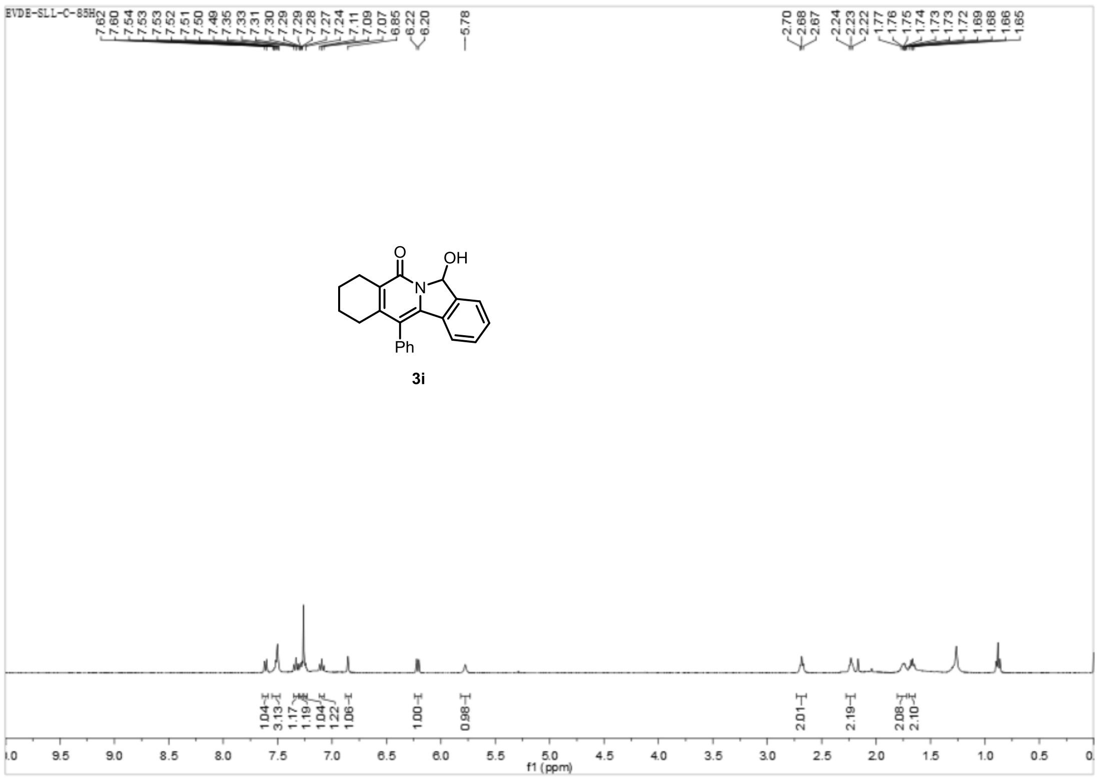

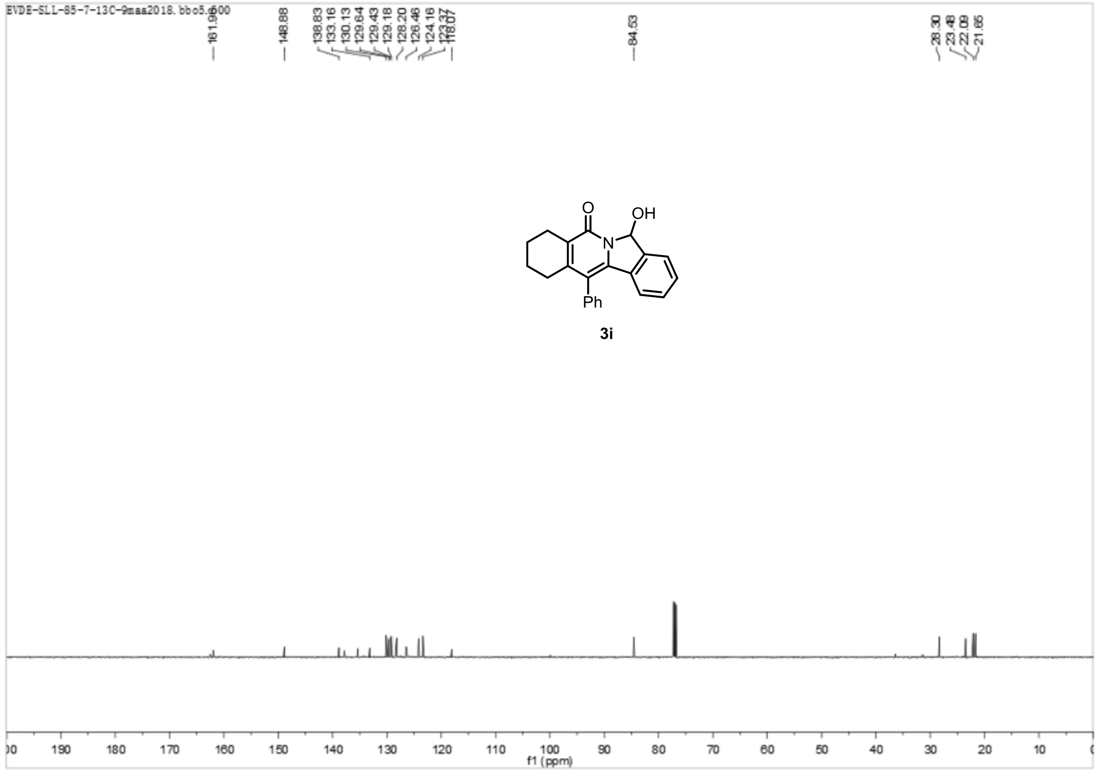

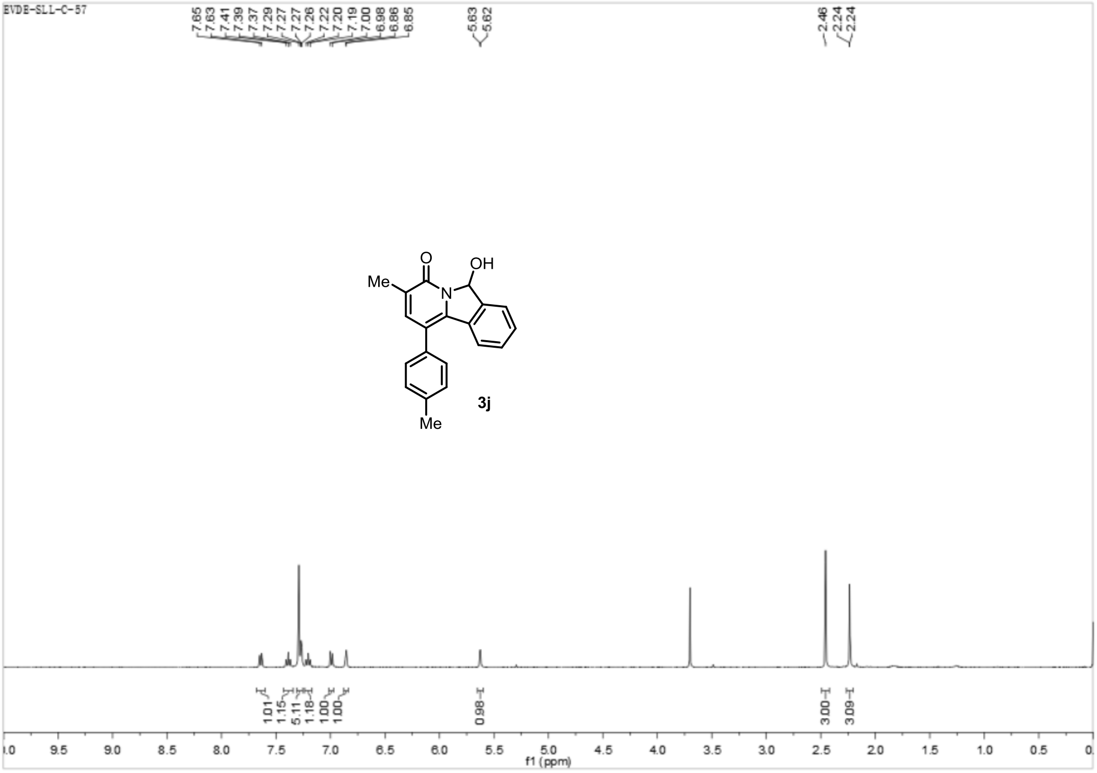
EVDE-SLL-C-57
7.65
7.63
7.41
7.39
7.37
7.29
7.27
7.27
7.26
7.22
7.20
7.19
7.00
6.98
6.86
6.85
5.63
5.62
2.46
2.24
2.24
Me
O
OH
N
Me
3j
1.01
1.15
5.11
1.18
1.00
1.00
0.98
3.00
3.09
f1 (ppm)

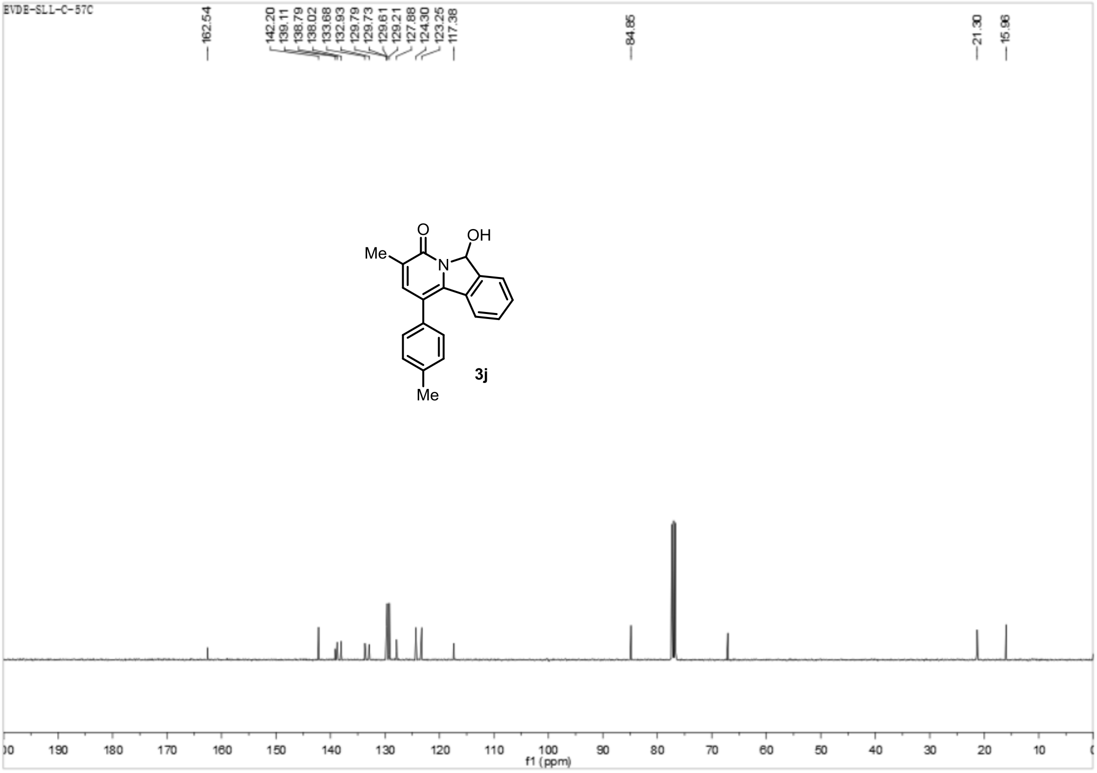
EVDE-SLL-C-57C
162.54
142.20
139.11
138.79
138.02
133.68
132.93
129.79
129.73
129.61
129.21
127.88
124.30
123.25
117.38
84.85
21.30
15.96
Me
O
OH
N
Me
3j
f1 (ppm)

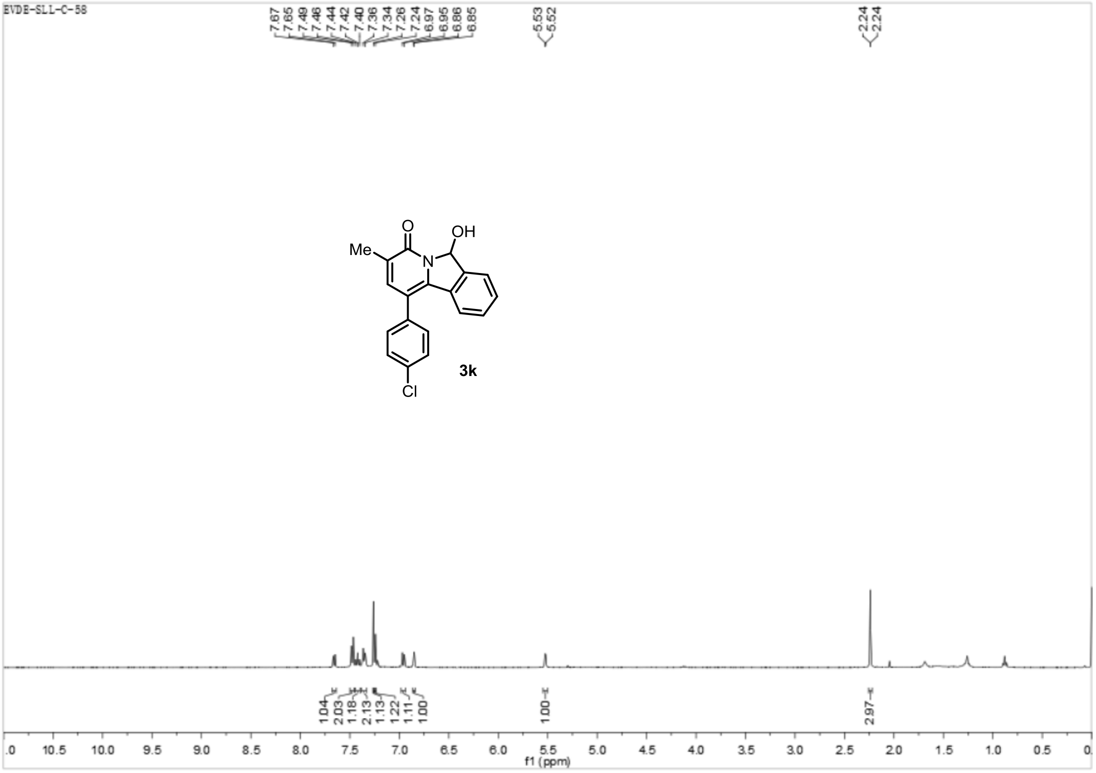
EVDE-SLL-C-58
7.67
7.65
7.49
7.46
7.44
7.42
7.40
7.36
7.34
7.26
7.24
6.97
6.95
6.86
6.85
5.53
5.52
2.24
2.24
Me
O
OH
N
Cl
3k
1.04
2.03
1.18
2.13
1.13
1.22
1.11
1.00
1.00
2.97
f1 (ppm)

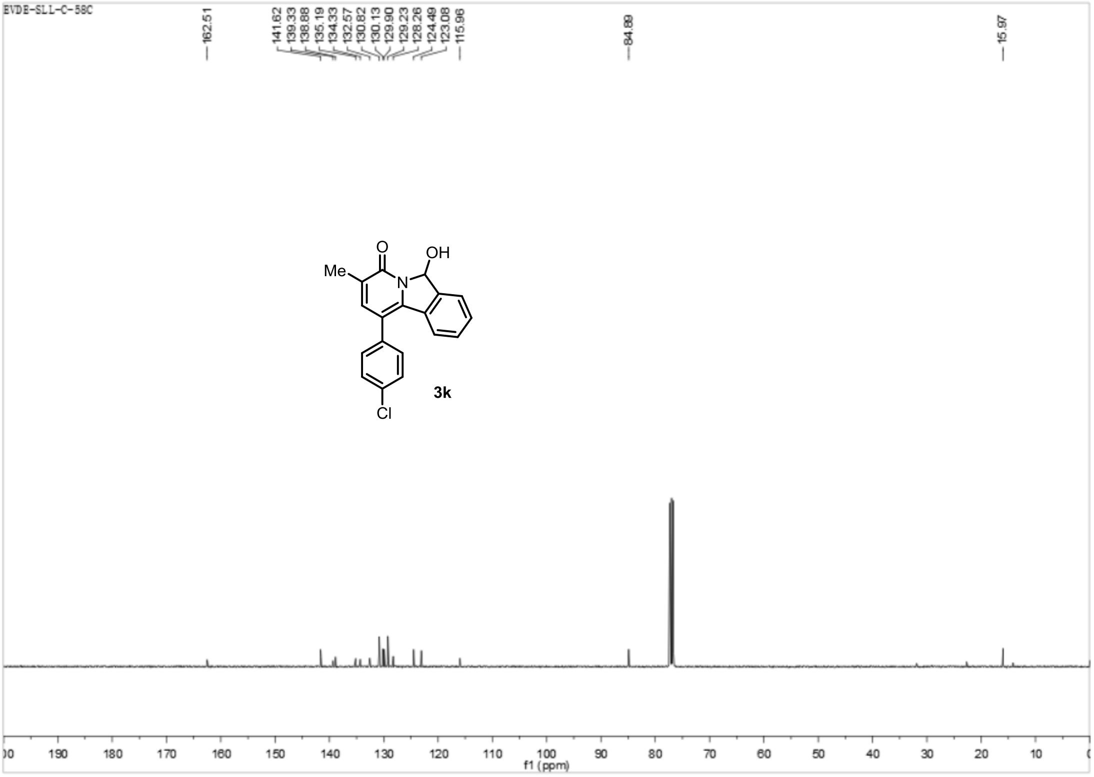
EVDE-SLL-C-58C
162.51
141.62
139.33
138.88
135.19
134.33
132.57
130.82
130.13
129.90
129.23
128.26
124.49
123.08
115.96
84.89
15.97
Me
O
OH
N
Cl
3k
f1 (ppm)

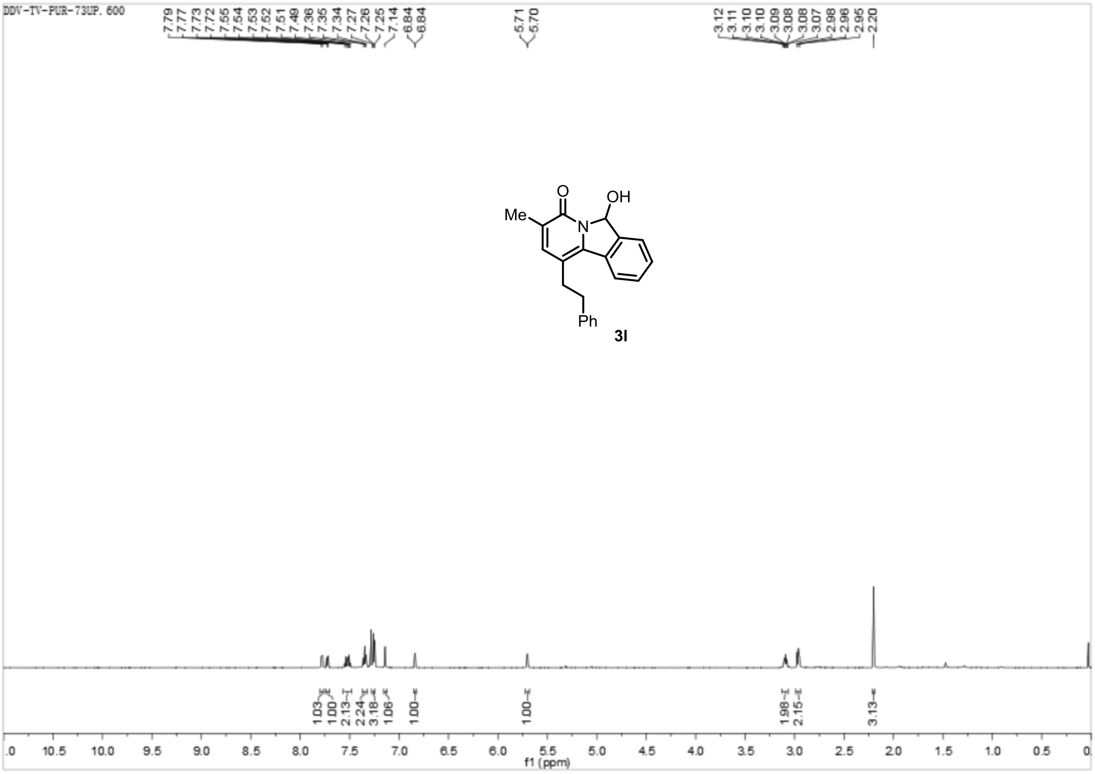
DDV-TV-PUR-73UP. 600
7.79 7.77 7.73 7.72 7.55 7.54 7.53 7.52 7.51 7.49 7.36 7.35 7.34 7.27 7.26 7.25 7.14 6.84 6.84
5.71 5.70
3.12 3.11 3.10 3.10 3.09 3.08 3.08 3.07 2.98 2.96 2.95 2.20
Me
O
N
OH
Ph
3l
1.03 1.00 2.13 2.24 3.18 1.06 1.00
1.00
1.98 2.15
3.13
f1 (ppm)

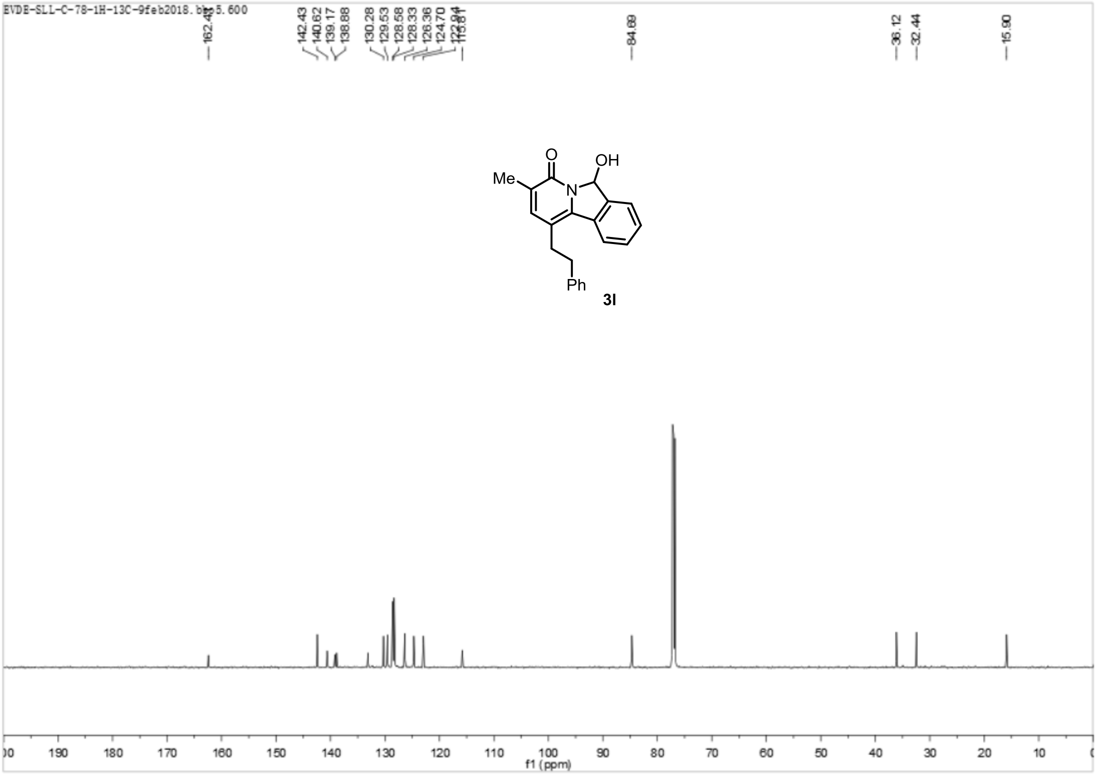
EVDE-SLL-C-78-1H-13C-9feb2018.
142.43 140.62 139.17 138.88 130.28 129.53 128.58 128.33 126.36 124.70
84.89
36.12 32.44
15.90
Me
O
N
OH
Ph
3l
f1 (ppm)

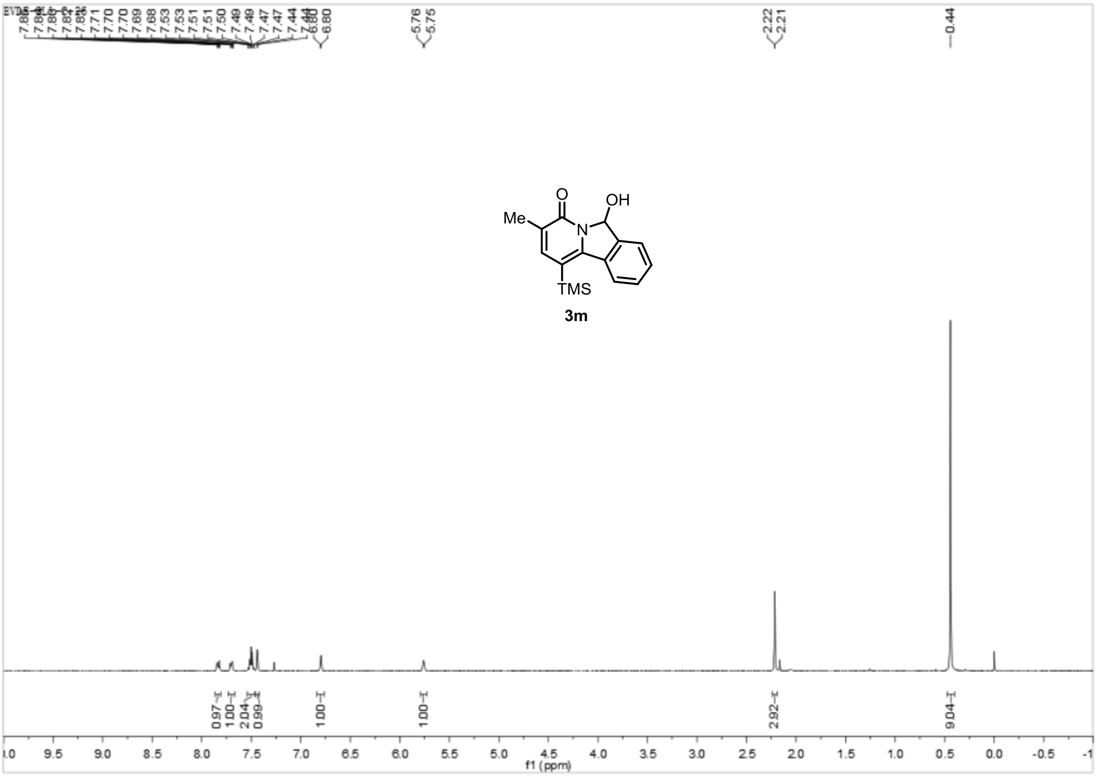
5.76
5.75
2.22
2.21
0.44
Me
O
OH
N
TMS
3m
0.97
1.00
2.04
0.99
1.00
1.00
2.92
9.04
f1 (ppm)

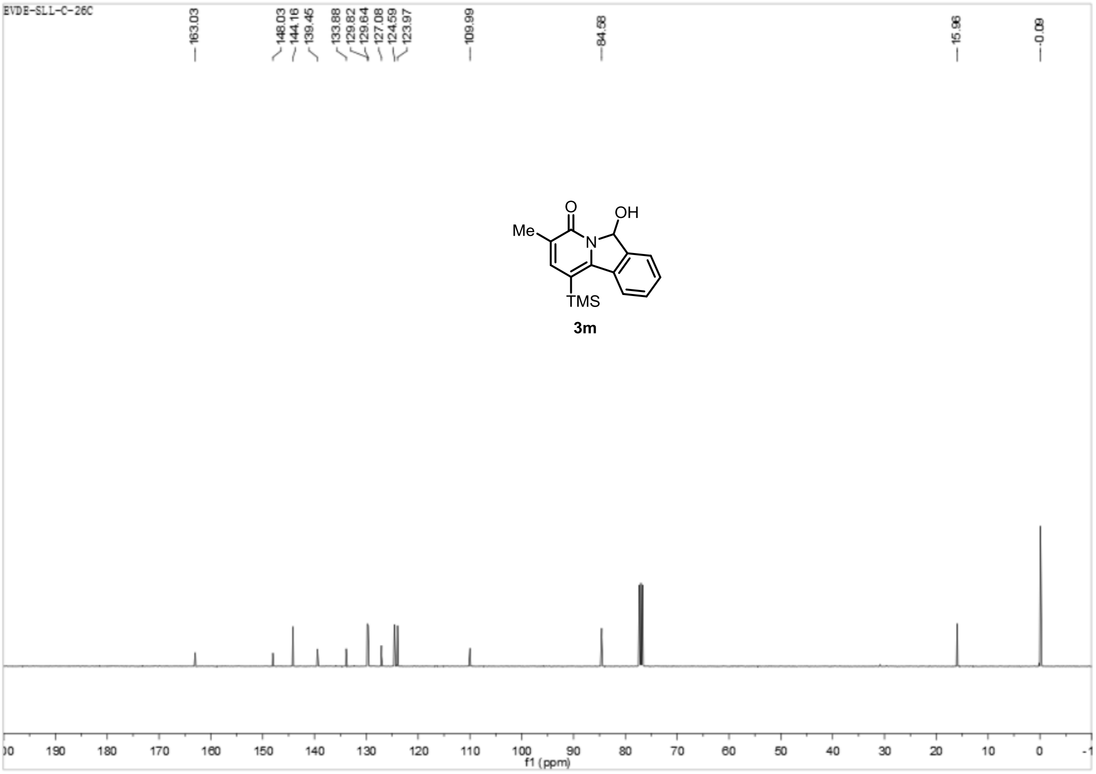
EVDE-SLL-C-26C
163.03
148.03
144.16
139.45
133.88
129.82
129.64
127.08
124.59
123.97
109.99
84.58
15.96
-0.09
Me
O
OH
N
TMS
3m
f1 (ppm)

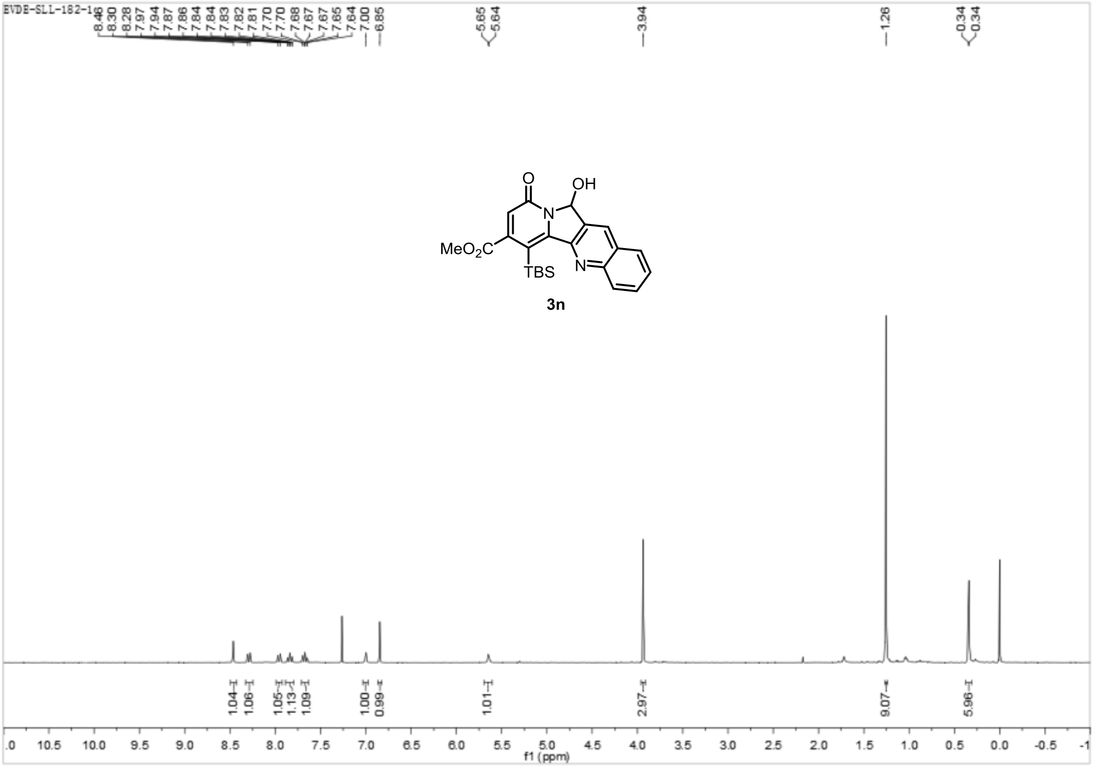

EVDE-SLL-182-1
8.46
8.30
8.28
7.97
7.94
7.87
7.86
7.84
7.84
7.83
7.82
7.81
7.70
7.68
7.67
7.65
7.64
7.00
6.85
5.65
5.64
3.94
1.26
0.34
0.34
O
OH
N
MeO2C
TBS
N
3n
1.04
1.06
1.05
1.13
1.09
1.00
0.99
1.01
2.97
9.07
5.96
f1 (ppm)

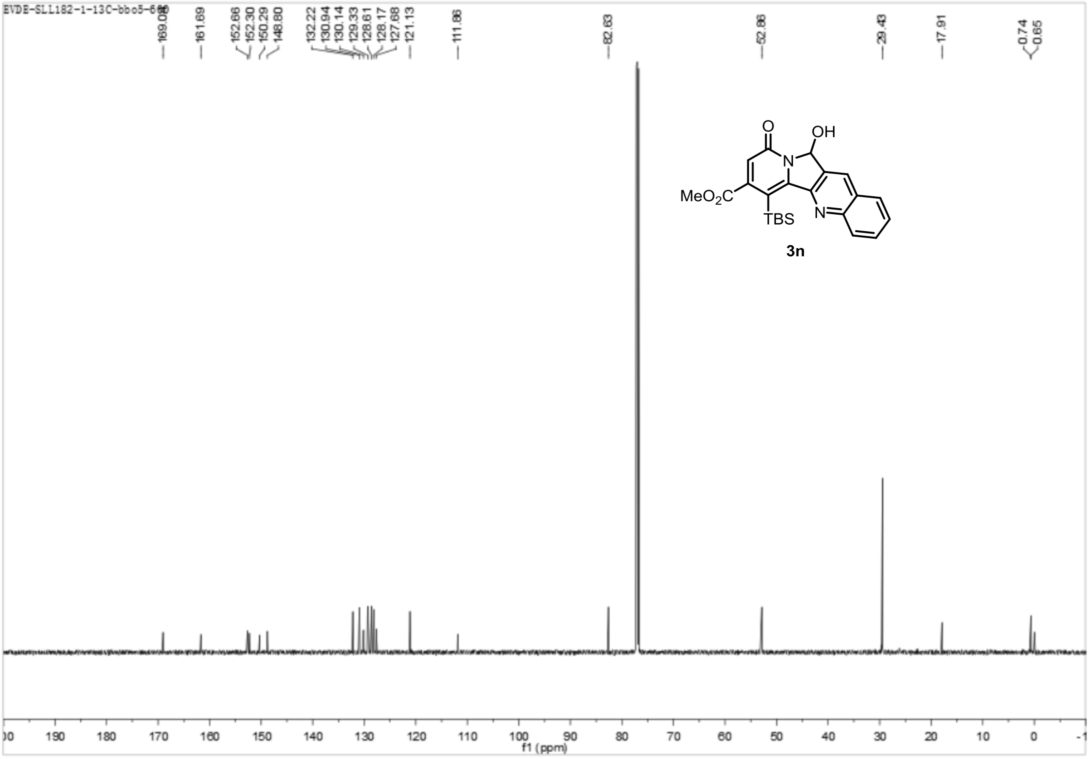

EVDE-SLL182-1-13C-bbo5-600
169.08
161.69
152.66
152.30
150.29
148.80
132.22
130.94
130.14
129.33
128.61
128.17
127.68
121.13
111.86
82.63
52.86
29.43
17.91
0.74
0.65
O
OH
N
MeO2C
TBS
N
3n
f1 (ppm)
